# Ayurveda botanicals in COVID-19 management: An *in silico* multi-target approach

**DOI:** 10.1371/journal.pone.0248479

**Published:** 2021-06-11

**Authors:** Swapnil Borse, Manali Joshi, Akash Saggam, Vedika Bhat, Safal Walia, Aniket Marathe, Sneha Sagar, Preeti Chavan-Gautam, Aboli Girme, Lal Hingorani, Girish Tillu

**Affiliations:** 1 AYUSH-Center of Excellence, Center for Complementary and Integrative Health, Interdisciplinary School of Health Sciences, Savitribai Phule Pune University, Pune, India; 2 Bioinformatics Centre, Savitribai Phule Pune University, Pune, India; 3 Serum Institute of India Pvt. Ltd., Pune, Maharashtra, India; 4 Department of Pharmaceutical Chemistry, L. J. Institute of Pharmacy, Sarkhej, Ahmedabad, India; 5 Pharmanza Herbal Pvt. Ltd., Anand, Gujarat, India; Weizmann Institute of Science, ISRAEL

## Abstract

The Coronavirus disease (COVID-19) caused by the virus SARS-CoV-2 has become a global pandemic in a very short time span. Currently, there is no specific treatment or vaccine to counter this highly contagious disease. There is an urgent need to find a specific cure for the disease and global efforts are directed at developing SARS-CoV-2 specific antivirals and immunomodulators. Ayurvedic *Rasayana* therapy has been traditionally used in India for its immunomodulatory and adaptogenic effects, and more recently has been included as therapeutic adjuvant for several maladies. Amongst several others, *Withania somnifera* (Ashwagandha), *Tinospora cordifolia* (Guduchi) and *Asparagus racemosus* (Shatavari) play an important role in *Rasayana* therapy. The objective of this study was to explore the immunomodulatory and anti SARS-CoV2 potential of phytoconstituents from Ashwagandha, Guduchi and Shatavari using network pharmacology and docking. The plant extracts were prepared as per ayurvedic procedures and a total of 31 phytoconstituents were identified using UHPLC-PDA and mass spectrometry studies. To assess the immunomodulatory potential of these phytoconstituents an *in-silico* network pharmacology model was constructed. The model predicts that the phytoconstituents possess the potential to modulate several targets in immune pathways potentially providing a protective role. To explore if these phytoconstituents also possess antiviral activity, docking was performed with the Spike protein, Main Protease and RNA dependent RNA polymerase of the virus. Interestingly, several phytoconstituents are predicted to possess good affinity for the three targets, suggesting their application for the termination of viral life cycle. Further, predictive tools indicate that there would not be adverse herb-drug pharmacokinetic-pharmacodynamic interactions with concomitantly administered drug therapy. We thus make a compelling case to evaluate the potential of these *Rasayana* botanicals as therapeutic adjuvants in the management of COVID-19 following rigorous experimental validation.

## Introduction

The, SARS-CoV-2 virus is responsible for causing the ongoing Coronavirus disease (COVID-19) pandemic [1]. Higher infectivity as compared to the SARS-CoV virus reported in 2003 and absence of a definite cure are the worrisome aspects of SARS-CoV-2 [2]. In the view of rapid spread in short period of time, the number of people infected globally is enormous (~over 2 million), and this poses a tremendous challenge to healthcare systems. COVID-19 has an age related skewed distribution of morbidity and an overall lethality although the numbers are changing as the disease is progressing [3]. The Centre for Disease Control and Prevention (CDC) reported that, COVID-19 patients with co-morbidities such as chronic lung diseases (e.g. asthma, COPD), hypertension, obesity, Type 2 Diabetes Mellitus (T2DM) are vulnerable to a higher mortality rate [4].

The SARS-CoV-2 virus primarily attacks lung alveoli for its replication. The Spike protein of the virus binds to Angiotensin Converting Enzyme-2 (ACE-2) receptors on the surface of type-II pneumocytes of alveolar lining, which are then internalised and the +ssRNA is released [5]. With the help of host ribosomal machinery and the RNA-dependent RNA polymerase (RdRp) enzyme SARS-CoV-2 synthesizes its polyproteins and multiplies its +ssRNA. The new copies of SARS-CoV-2 are released into the alveolar sac by destroying the infected pneumocytes. The inflammatory mediators released after pneumocyte damage recruit immune cells at the infected site. Macrophages release inflammatory cytokines into the blood leading to vasodilation of blood vessels increasing capillary permeability of endothelial cells. Neutrophils release reactive oxygen species (ROS) and proteases to destroy viruses which also damage normal pneumocytes and generate cellular debris in alveolar space. These inflammatory and immune responses result into alveolar consolidation leading to increased respiratory rate followed by cough. The systemic inflammatory response acts as messengers to hypothalamus to increase body temperature [6]. In some patients the cytokine response goes out of control leading to excessive collateral damage to organs with a possible progression to death [7, 8].

Presently there is no cure for the disease and the treatment is symptomatic. In some countries, patients are being treated using existing combinations of antivirals used for other viral infections [9]. Clinical evidence explaining the efficiency of these antivirals against SARS-COV-2 are limited and not defined [10]. Therefore, for developing specific therapies as well as for boosting speed and scale of clinical evaluation WHO launched Solidarity clinical trial on April 8, 2020. This includes screening of four study treatments in comparison with standard of care. Based on available experimental data, remdesivir, lopinavir/ritonavir, lopinavir/ritonavir with interferon beta-1α, and chloroquine or hydroxychloroquine are the chosen study drugs [11]. For patients with co-morbidities, it is inevitable to take daily medication along with COVID-19 managing drugs. Therefore, it is required to have safe pharmacotherapy for COVID-19 that can be co-prescribed with WHO solidarity trial drugs and commonly prescribed drugs such as anti-hypertensive, anti-asthmatic and anti-diabetic. Administering plasma of a recovered patient to the critically ill COVID-19 patients also seems promising [12]. Hydroxychloroquine which is being used in many countries for COVID-19 treatment under emergency circumstances, seems to have limited beneficial evidence [13, 14]. There is indeed a great rush to find the holy grail for COVID-19 in terms of vaccines and therapeutics against SARS-CoV-2. Several pharmaceutical companies have announced clinical trials for drug and vaccine candidates. However, it may take a long time to reach the community.

Traditional medicine systems such as Ayurveda, have a holistic approach of considering mind-body-physiology to deal with disease conditions [15]. The Ayurvedic philosophy suggests delivering “a group of phytoconstituents” that holds potential to give adaptogenic, immunomodulatory effects and also act on drug targets [16–18]. Thus, in Ayurveda, “*Rasayana* botanicals” are used for rejuvenation by boosting the immune system and alleviating disease condition [19–21]. Of several known botanicals *Asparagus racemosus* (AR) commonly known as Shatavari, *Tinospora cordifolia* (TC) known as Guduchi and *Withania somnifera* (WS) known as Ashwagandha, are known to modulate the immune system and possess antiviral activities [8, 19, 20, 22, 23].

The ideal COVID-19 therapy should show (a) antiviral properties against SARS-COV-2, (b) be safe for concomitantly administered drugs like anti-hypertensive, anti-diabetic, anti-asthmatics, and drugs those are used in respiratory tract infections (c) should modulate immune system with rejuvenation ability (mainly for cardio-respiratory and nervous system) (d) should show therapeutic adjuvant activity with drugs used in WHO Solidarity trials. In this work we explore if the botanicals WS, TC and AR can fill in the gap. Phytochemicals present in AR, TC and WS were identified using chromatographic techniques. A network pharmacology model was used to identify and depict the interactions of phytochemicals with molecular targets in the immune system to unravel their immunomodulatory role. Further, the phytoconstituents were docked to three molecular targets of SARS-CoV-2 to assess the potential for antiviral activity. Further, predictive tools were used to assess the potential of interactions between phytoconstituents and commonly prescribed drugs. The results present a compelling case that Ayurvedic *Rasayana* botanicals have the potential for immunomodulatory and antiviral activity and could be used as therapeutic adjuvant for COVID-19 management following rigorous experimentation.

## Materials and methods

### Preparation of extracts and phytochemical characterization

#### Plant material and extract preparation

The *Withania somnifera* (WS) roots used in the experiment were collected from Anand (GJ, India) and authenticated. A hydroalcoholic and water extraction was carried out for WS root powder. *Withania somnifera* hydroalcoholic extract (WSHA) and *Withania somnifera* water extract (WSW) were 10 and 12% in weight and stored at 4 to 8°C for further studies. The *Tinospora cordifolia* (TC) sample was collected from Ratnagiri (MH, India) and authenticated. The stem was dried and powdered. A hydroalcoholic and water extraction was carried out, giving 6.6% and 7% yield. *Tinospora cordifolia* hydroalcoholic extract (TCHA) and *Tinospora cordifolia* water extract (TCW) were stored at 4-8°C. The *Asparagus racemosus* (AR) roots used in the experiment were collected from Anand (GJ, India) and authenticated. The dried and pulverized AR sample (100 g) was extracted with hydroalcohol and water as *Asparagus racemosus* hydroalcoholic extract (ARHA) and *Asparagus racemosus* water extract (ARW) giving 30% and 20% yield, respectively. The voucher specimen samples were submitted, and plant authentication was done by the Botanical Survey of India Arid Zone Regional Centre Jodhpur (IN). Further, all specimens were deposited at the Department of Botany, Pharmanza Herbal Pvt Ltd, (Anand, Gujarat, IN).

#### Chemicals and reagents

The compounds used in the WS study are withanoside IV (WS1), withanoside VII (WS2), viscosalactone B (WS3), 27-hydroxywithanone (WS4), dihydrowithaferin A (WS5), withaferin A (WS6), withanoside V (WS7), 12-deoxywithastramonolide (WS8), withanolide A (WS9), withanone (WS10), and withanolide B (WS11). Compounds WS1 and WS9 were procured from USP (MD, USA), and compounds WS7, WS8, WS10 and WS11 were procured from phytocompounds (Bangalore, IN); compound WS6 was procured from Chromadex (CA, USA). Compounds WS (2−5) were isolated from WS’s roots in the in-house laboratory by previously reported methods.

The reference compounds used in the TC study are cordifolioside-A (TC1), 20-β-hydroxy ecdysone (TC2), tinosporaside (TC3), 8-hydroxy tinosporide (TC4), tinosporide (TC5), columbin (TC6). Compound TC1 and TC6 were procured from Chemfaces (Hubei, CN) and compound TC2 from Chromadex (CO, USA). The compounds TC (3–5) were isolated from TC’s stems in the in-house laboratory by previously reported methods.

The reference compound used in the AR study is shatavarin-IV (AR1) was procured from Phytocompounds (Bangalore, IN). UHPLC-PDA confirmed the minimum purity of these standards as > 90% by analyzing concentrated standards (1 mg/mL) in acetonitrile and water (1:1) isocratic elution. The procurement of acetonitrile and methanol of HPLC grade was from Rankem, IN, and MS grade water was from JT Baker, FS, IN.

Instrument Condition for UHPLC−PDA study for WS, TC, and AR analysis: The instrument used for UHPLC analysis was Shimadzu Nexera X2 (Shimadzu Tech., Kyoto, Japan), consisting of a quaternary pump (LC-30AD), autosampler (SIL-30AC), column oven (CTO-20AC), and data analyzed using the Lab Solution software (Version 6.80).

#### Chromatographic conditions for UHPLC−PDA analysis for WS compounds

The WS compounds were separated on a Phenomenex Luna 5 μm C8 (2) 100 Å column (250 mm × 4.6 mm × 5 μm). The column temperature was maintained at 35°C. The mobile phase was composed of water (A) and acetonitrile (B) with a gradient elution program with a detection wavelength of 227 nm with a run time of 50 min [24].

#### Preparation of the standard solution

The stock solution of each of the standard compounds WS (1−11) was prepared with a concentration of 100 μg/mL in methanol. Five different concentration levels of each compound were injected in triplicate to prepare the calibration curves. The calibration curves were formed in the range of between 1–150 μg/mL for compounds WS (1−4) and WS (6−11) and 1−100 μg/mL for compound WS5. All the working solutions were stored at 4°C.

#### Sample solutions

The samples were weighed and diluted with methanol (100 mg/10 mL), followed by sonication for 30 min. This sample solution was passed through a 0.22 μm filter and injected into the UHPLC system for analysis.

#### Chromatographic conditions for UHPLC−PDA analysis for TC compounds

For TC compounds, a Phenomenex Luna C18 (2) 100Ă column (4.6 X 250 mm, 5.0μm) was used for separation. The mobile phase consisted of solvent A (0.1% phosphoric acid in water) and solvent B (acetonitrile). The gradient elution at a flow rate of 1.00 mL/min with injection volume was set at 20 μL, and the detection wavelength was set at 210nm. The run time of the method was set at 45 min [25].

#### Preparation of the standard solution

The stock solution of TC (1–5) compounds (500 μg/mL) were separately prepared by dissolving 1.0 mg of accurately weighed standards in 5 mL methanol to make up the volume up to 10 mL methanol in an amber-coloured volumetric flask. The stock solution was further diluted as per the requirement for preparing a working solution (methanol: water). Five different concentration levels of each compound were injected in triplicate to prepare the calibration curves. The range of calibration curves was between 0.5–120 μg/mL for Shatavari. The working solutions were stored at 4°C.

#### Sample solutions

The sample extraction method was employed to extract the target analytes from the TC. The samples were accurately weighed, and methanol: water (70:30) (200 μg/ 10 mL) was added, followed by sonication for 30 min. This sample solution was passed through a 0.22 μm filter and injected into the UHPLC system for analysis.

#### Chromatographic conditions for UHPLC−PDA analysis for AR compounds

For AR analysis, Shimadzu C18 (2) 100Ă column (4.6 X 250 mm, 5.0 μm) was used for separation. The mobile phase consisted of solvent A (water) and solvent B (acetonitrile). The gradient elution at a flow rate of 1.00 mL/min with injection volume was set at 20 μL, and the detection wavelength was set at 210nm. The run time of the method was set at 55 min [26].

#### Preparation of the standard solution

The stock solution of shatavarin-IV RS was prepared with a concentration of 250 μg/ml in methanol. Five different concentration levels were injected in triplicate to prepare the calibration curves in the range of between 3.9–250 μg/mL for shatavarin-IV. The working solutions were stored at 4°C for further use.

#### Sample solutions

The simple and rapid method was employed to extract the target analytes from the AR. The samples were accurately weighed, and methanol (300μg/ 10 mL) was added, followed by sonication for 30 min. This sample solution was passed through a 0.22 μm filter and injected into the UHPLC system for analysis.

#### Application of UHPLC-PDA in the quantification of samples by external standard calibration

The water and hydroalcoholic extracts of WS, TC, and AR were analyzed (*n* = 3) by UHPLC-PDA method as in the above paragraphs. The quantification is done by an external standard calibration method. Experimental data analysis and parameter calculation were achieved using Office Excel 2010 (Microsoft, Redmon, WA, USA).

#### Mass spectrometric analysis of the WS, TC and AR extracts

Whereas MS was carried out at Bioanalytical Technologies Pvt. Ltd., Pune and Central Instrumentation facility (CIF), Savitribai Phule Pune University, Pune. The MS grade Methanol, Water and Formic acid were used as solvents. The MS: 4000 QTrap were used with ESI Q1 Positive mode. 10 mg of each sample was weighed in a 10 ml volumetric flask. The sample was dissolved up to the mark with methanol: water (80:20) V/v + 0.1% formic acid. The sample was filtered through 0.22-micron filter. 1ml of filtrate was transferred to 10 ml volumetric flask and volume was made-up with HPLC grade methanol and filtered through 0.22 micron before injection.

### Network pharmacology

The network pharmacology approach was followed to explore the role of *Rasayana* botanicals i.e. AR, TC, and WS in immunomodulation. Briefly, the qualitatively characterized phytochemicals (also referred as bioactives) from extracts were queried in PubChem database (https://pubchem.ncbi.nlm.nih.gov/) to obtain sdf files [27]. These files were uploaded to BindingDB database (https://www.bindingdb.org/bind/index.jsp) to retrieve putative protein targets of uploaded bioactives that had a similarity score of with ≥0.7 with co-crystalized ligands [28]. Gene names for compiled human protein targets were retrieved from UniProt Knowledgebase (http://www.uniprot.org/) [29]. Simultaneously, the KEGG pathway database (https://www.genome.jp/kegg/pathway.html) was mined to obtain proteins/genes involved in human immune pathways [30]. The KEGG database enlists twenty pathways related to immune system. The entire data was collected and analysed in Microsoft Excel to identify putative immune-associated protein targets of bioactives of AR, TC, and WS. The network of available data was constructed with eloquent representation using Cytoscape 3.7.2 (https://cytoscape.org/) software [31].

### Molecular docking and simulations

All 31 phytoconstituents, including 11 from AR, 10 from TC and 10 from WS were considered in this study. All 31 compounds were downloaded from Pubchem in sdf format including 3D coordinates [27]. Three drug targets of SARS-CoV-2 were considered namely, the Receptor Binding Domain of Spike Protein (RBD), the Main Protease (Mpro) and the RNA dependent RNA polymerase (RdRp). Docking was performed with AutoDock 4.2.6 assembled in the PyRx 0.8 Virtual Screening Tool [32, 33]. For the Main protease, the crystal structure of the protease complexed with 2-cyclohexyl-{N}-pyridin-3-yl-ethanamide (GWS) (PDB ID: 5R84 Chain A, resolution 1.83Å) was considered for structural studies. A grid box was prepared with dimensions 60×60×60Å centred on the protein around residues which interacted with co-crystallized ligand GWS representing pockets S1-S3, with grid spacing of 0.375Å. For the RNA-dependent RNA polymerase, the PDB ID: 6M71 Chain A, resolution 2.90 Å, was considered for structural studies [34]. A grid box of dimensions 100×100×100Å with grid spacing of 0.375Å was centred around the RNA binding site. The cryoEM structure of Receptor Binding Domain of Spike protein with ACE2-B0AT1 complex (PDB ID: 6M17 Chain E, resolution 2.90 Å) was considered for structural studies [35]. A grid box was prepared with dimensions 90×90×90Å and spacing 0.375Å and was centred on the protein residues: Lys417, Tyr453, Gln474, Phe486, Gln498, Thr500 and Asn501. Prior to docking, all compounds were subjected to energy minimization using the inbuilt Open Babel module in Pyrx [36]. 50 docking runs were performed for each phytochemical with each drug target, with a population size of 150 and 250000 energy evaluations. All the other algorithm parameters were kept default. The final pose of the phytoconstituents was selected based on its docking score. The grid dimensions for each protein system are given in S2 Table in S1 File.

Simulations of the protein-ligand complexes were performed using GROMACS version 2019.6 [37]. Parameters for the ligand were obtained from cgenff using Ligand Reader & Modeler module of CHARMM-GUI [38] while CHARMM36 [39] parameter set was used for protein. Briefly, the complexes were solvated using explicit water and ions were added to neutralize the systems. Systems were minimized using Steepest Descent algorithm followed by equilibration using NVT (constant number of molecules, Volume and Temperature) and NPT (constant number of molecules, Pressure and Temperature) ensemble for 100 ps each. Berendsen method was used for temperature coupling. Pressure coupling was carried out by Parrinello-Rahman method. Periodic Boundary Conditions were applied, and long-range interactions were treated using Particle Mesh Ewald (PME) method. All simulations were performed for 30 ns. Analysis was performed using standard GROMACS and VMD tools.

### Predicting herb-drug interactions

Considering the COVID-19 complex pathogenesis there is concomitant administration of multiple drugs with herbs which may lead to risk of herb-drug interactions (HDIs) [40]. Therefore, the probable pharmacokinetic-pharmacodynamic herb drug interactions (HDIs) were explored based on data generated from Swiss-ADME (http://www.swissadme.ch/) [41] and available literature.

## Results

### A total of 31 major phytoconstituents are identified from *Withania somnifera*, *Tinospora cordifolia* and *Asparagus racemosus*

**Fig 1:**
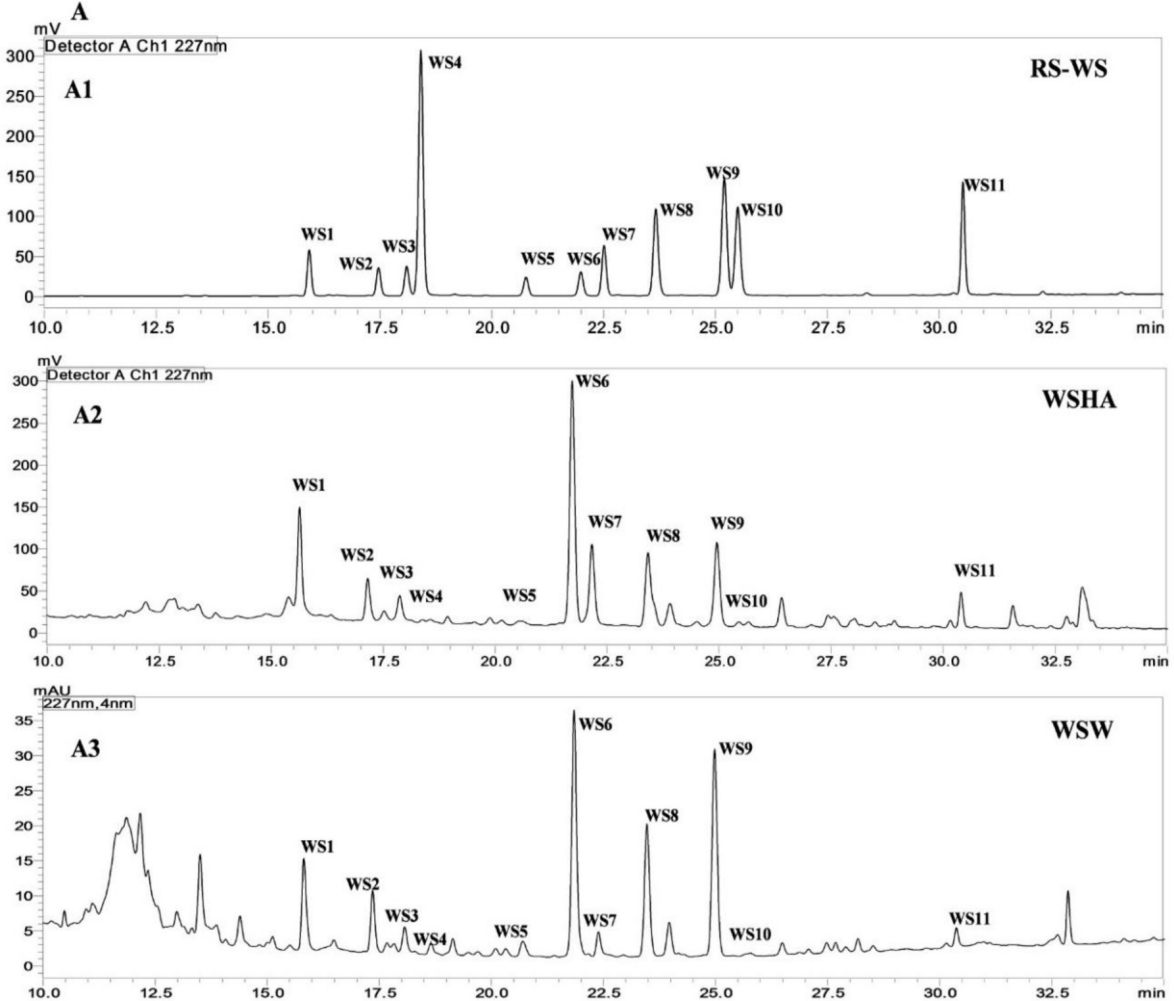
UHPLC−PDA chromatogram of reference compounds (RS) and samples with detection for (**A**) *Withania somnifera* at 227 nm comprising compounds **WS (1−11)** in the (**A1**) reference compounds (**A2**) WSHA (**A3**) WSW; where withanoside IV (**WS1**), withanoside VII (**WS2**), viscosalactone B (**WS3**), 27-hydroxywithanone (**WS4**), dihydrowithaferin A (**WS5**), withaferin A (**WS6**), withanoside V (**WS7**), 12-deoxywithastramonolide (**WS8**), withanolide A (**WS9**), withanone (**WS10**), and withanolide B (**WS11**).

**Fig 2:**
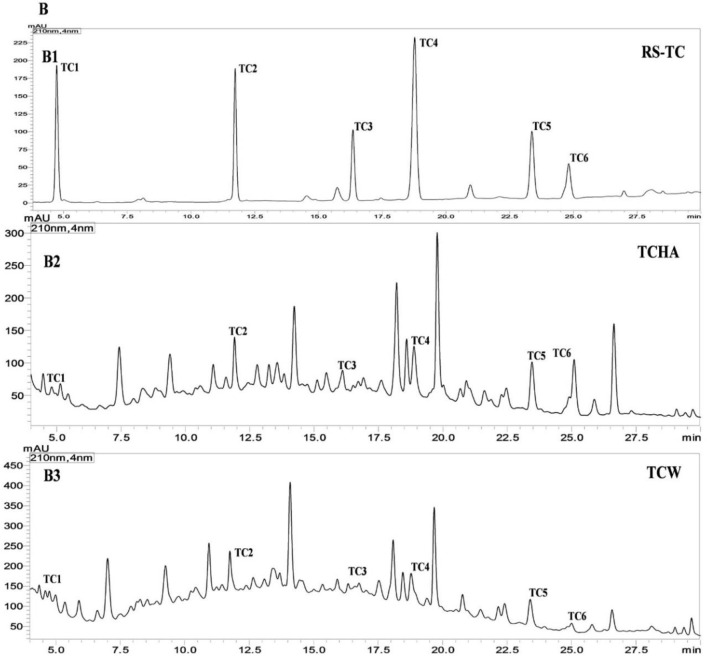
UHPLC−PDA chromatogram of reference compounds (RS) and samples with detection for (**B**) *Tinospora cordifolia* at 210 nm for **TC (1–6)** in the (**B1**) reference compounds (**B2**) TCHA (**B3**) TCW where cordifolioside-A (**TC1**), 20-β-hydroxy ecdysone (**TC2**), tinosporaside (**TC3**), 8-hydroxy tinosporide (**TC4**), tinosporide (**TC5**), columbin (**TC6**).

**Fig 3:**
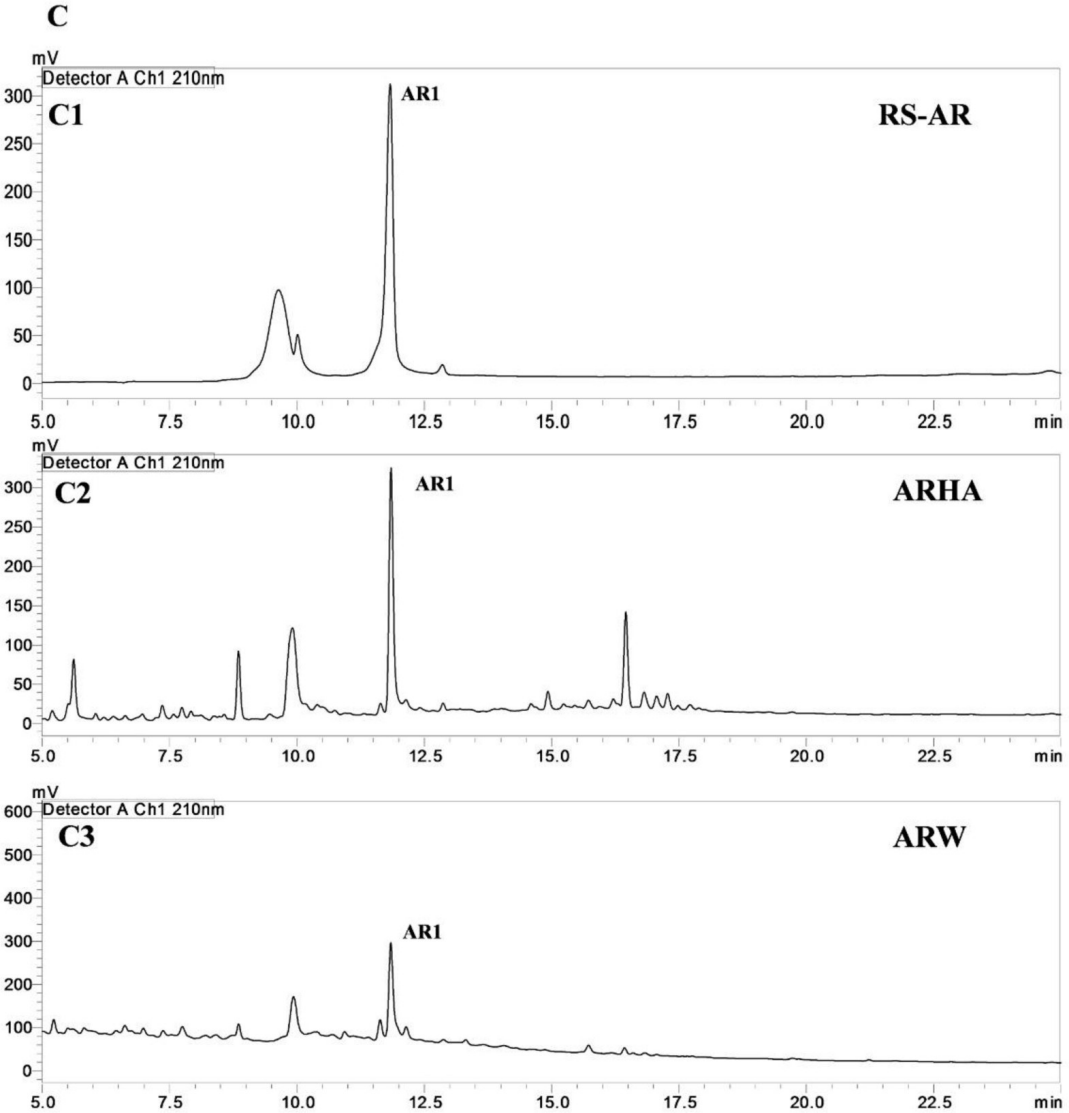
UHPLC−PDA chromatogram of reference compounds (RS) and samples with detection for (**C**) *Asparagus racemosus* at 210 nm for **AR-1**in the (**C1**) reference compound **AR1**(**C2**) ARHA (**C**)3 ARW; where shatavarin-IV (**AR1**).

**Fig 4:**
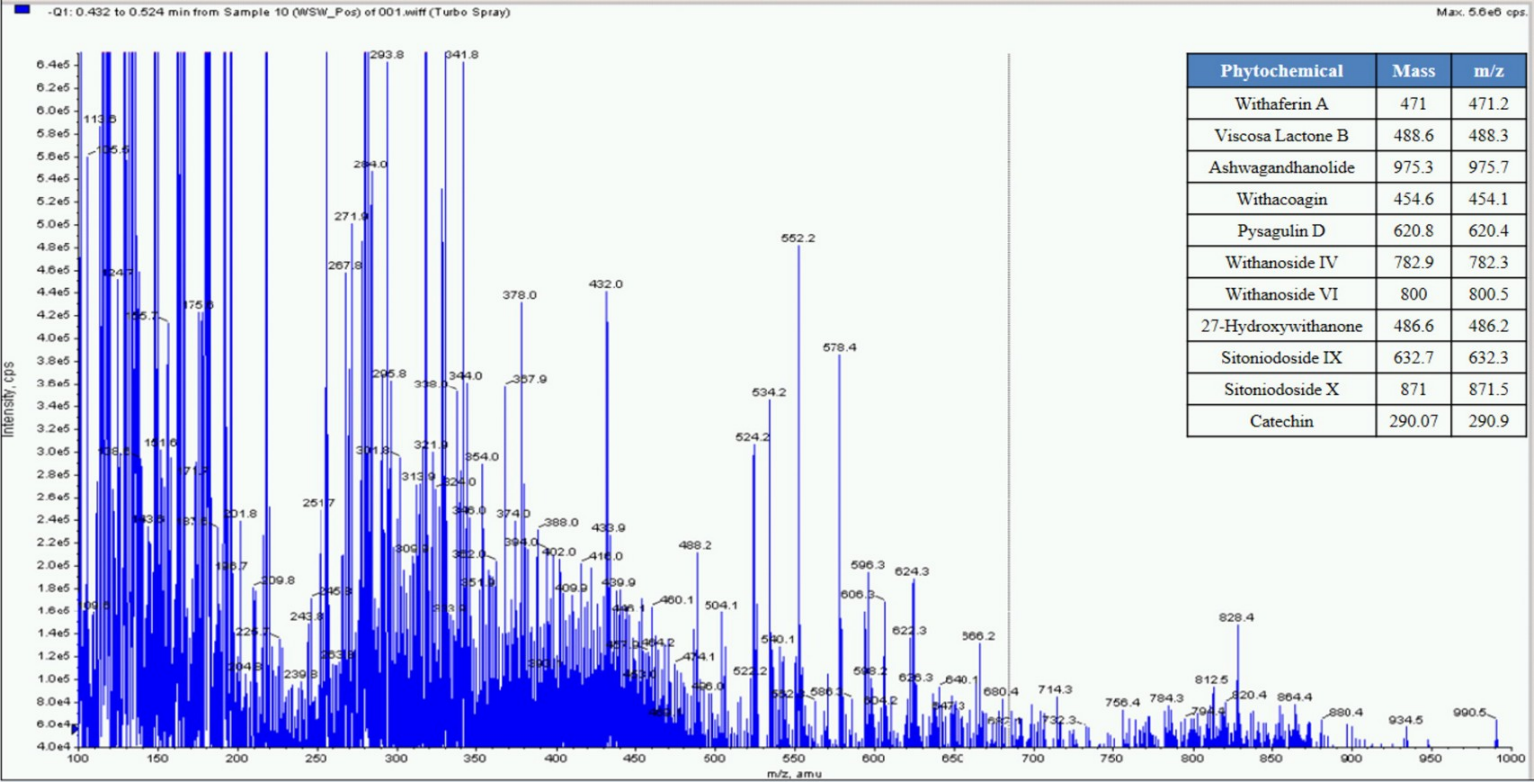
Mass spectrometer fingerprinting of *Withania somnifera* water extract (WSW).

**Fig 5:**
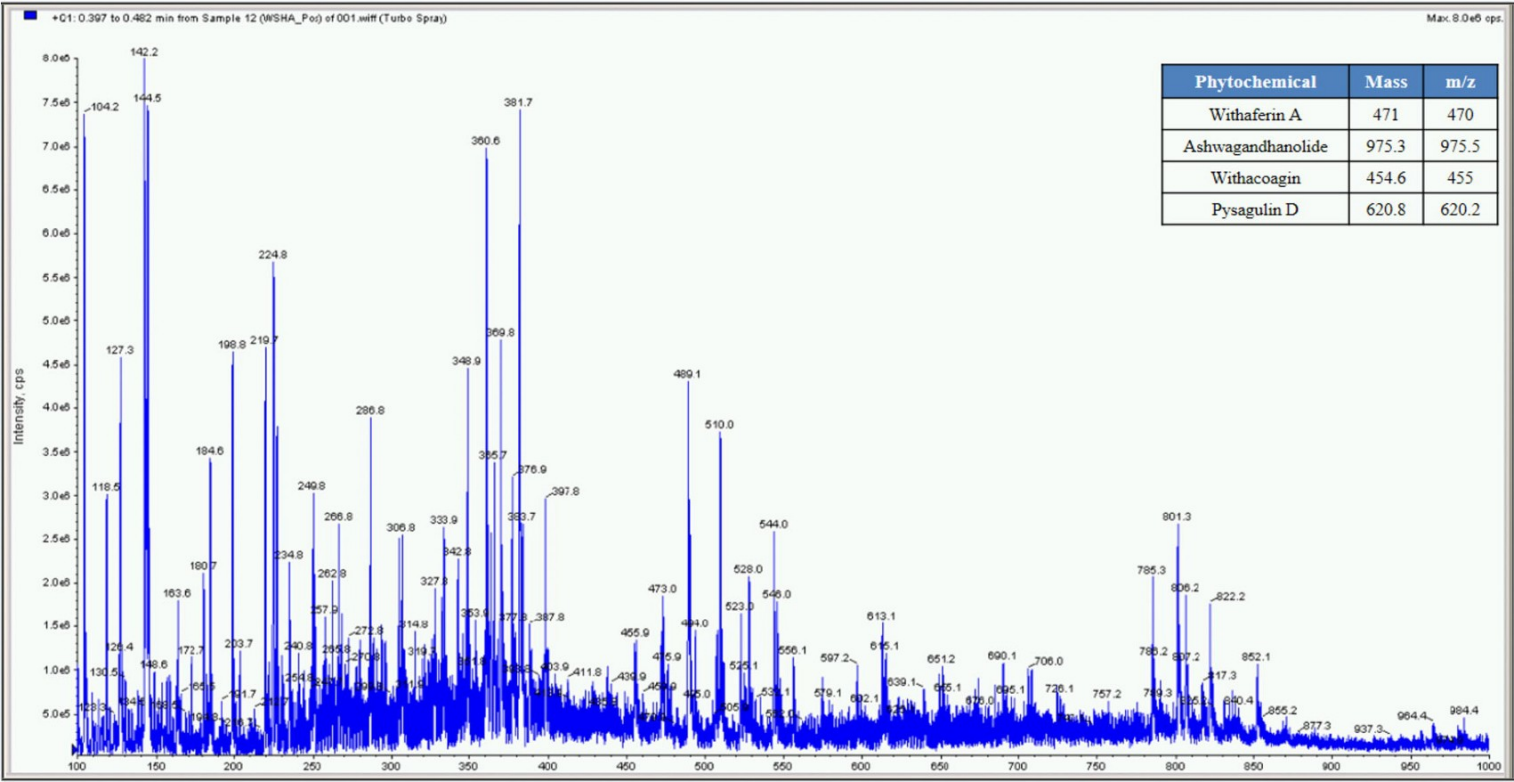
Mass spectrometer fingerprinting of *Withania somnifera* hydro-alcoholic extract (WSHA).

**Fig 6:**
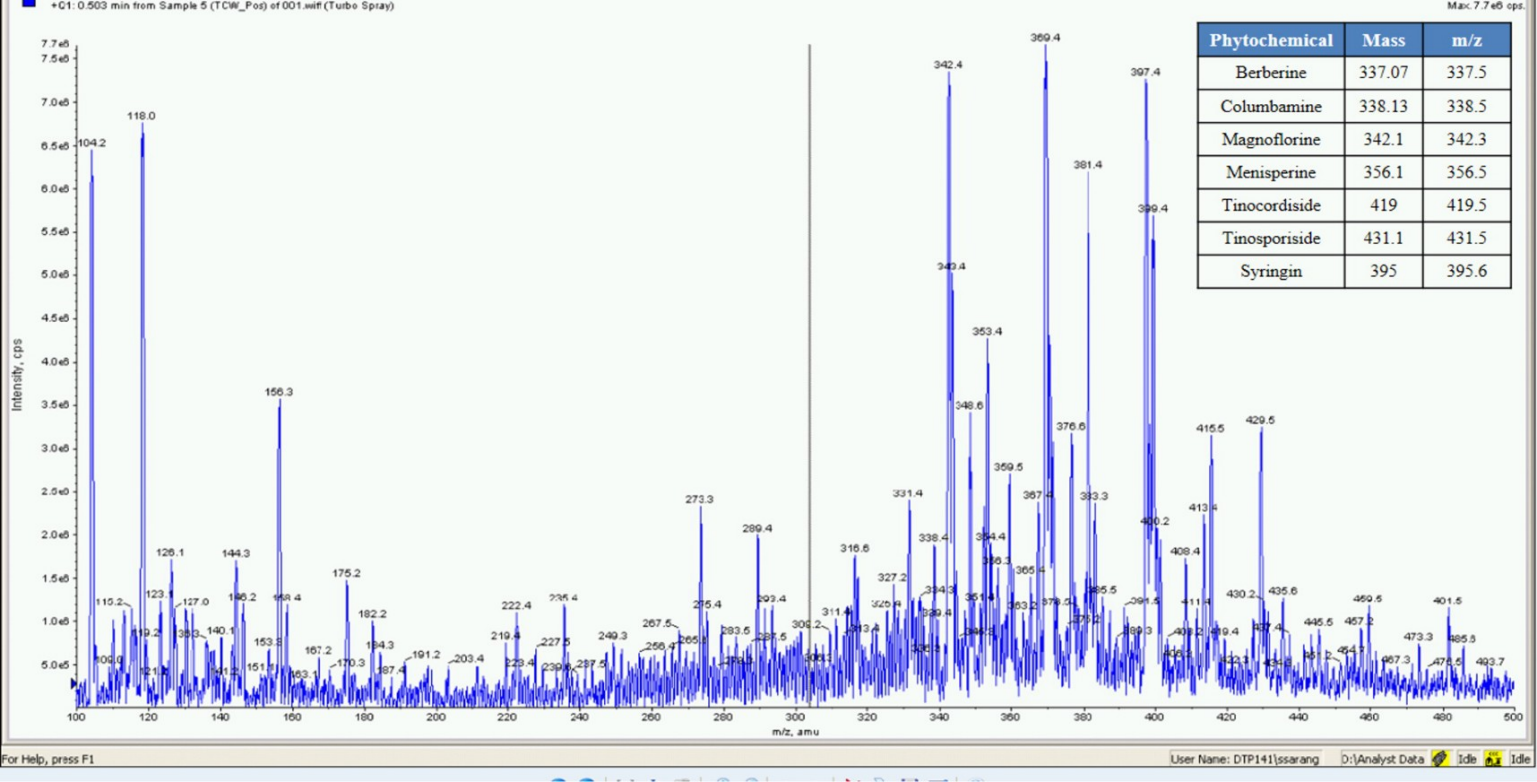
Mass spectrometer fingerprinting of *Tinospora cordifolia* water extract (TCW).

**Fig 7:**
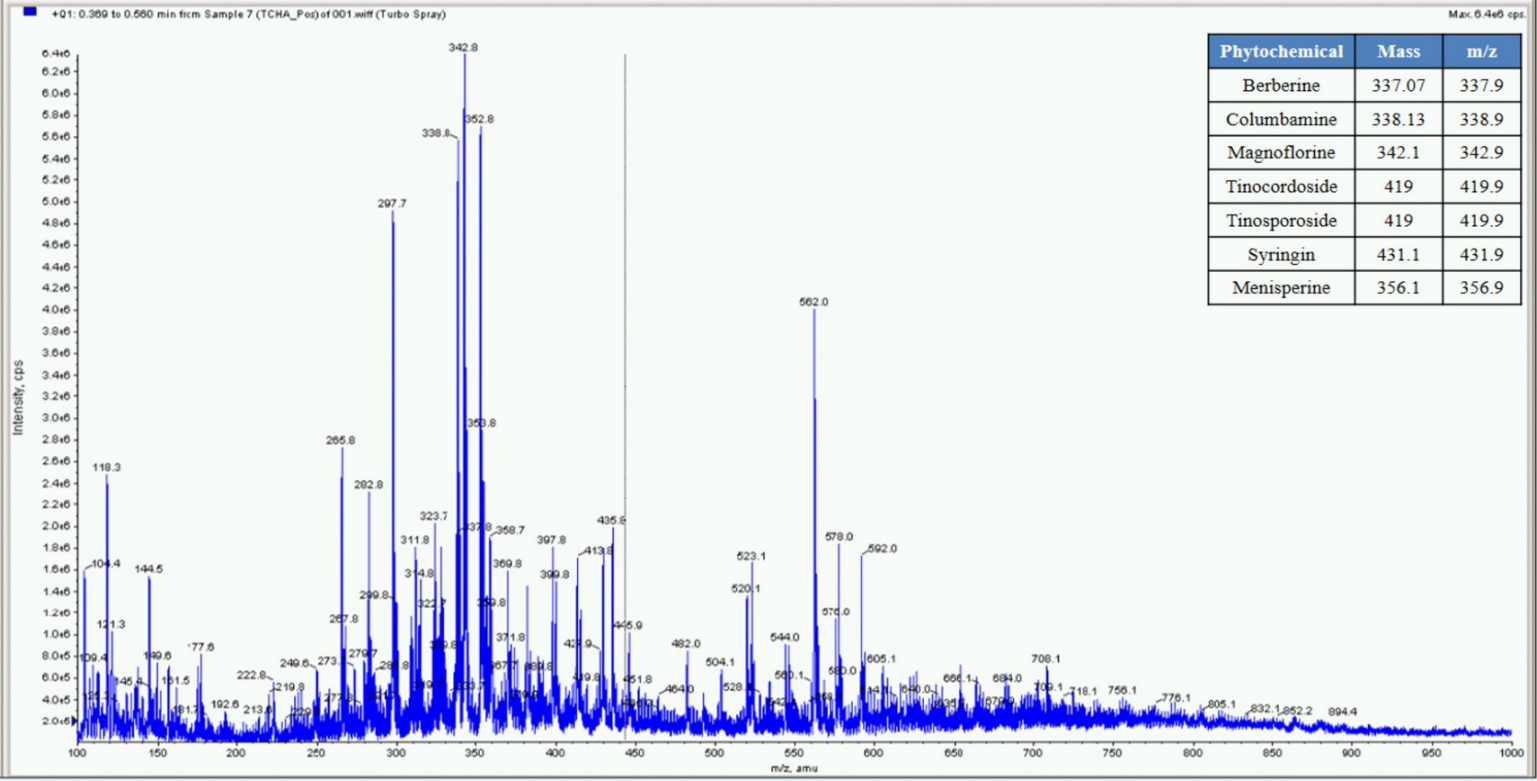
Mass spectrometer fingerprinting of *Tinospora cordifolia* hydro-alcoholic extract (TCHA).

**Fig 8:**
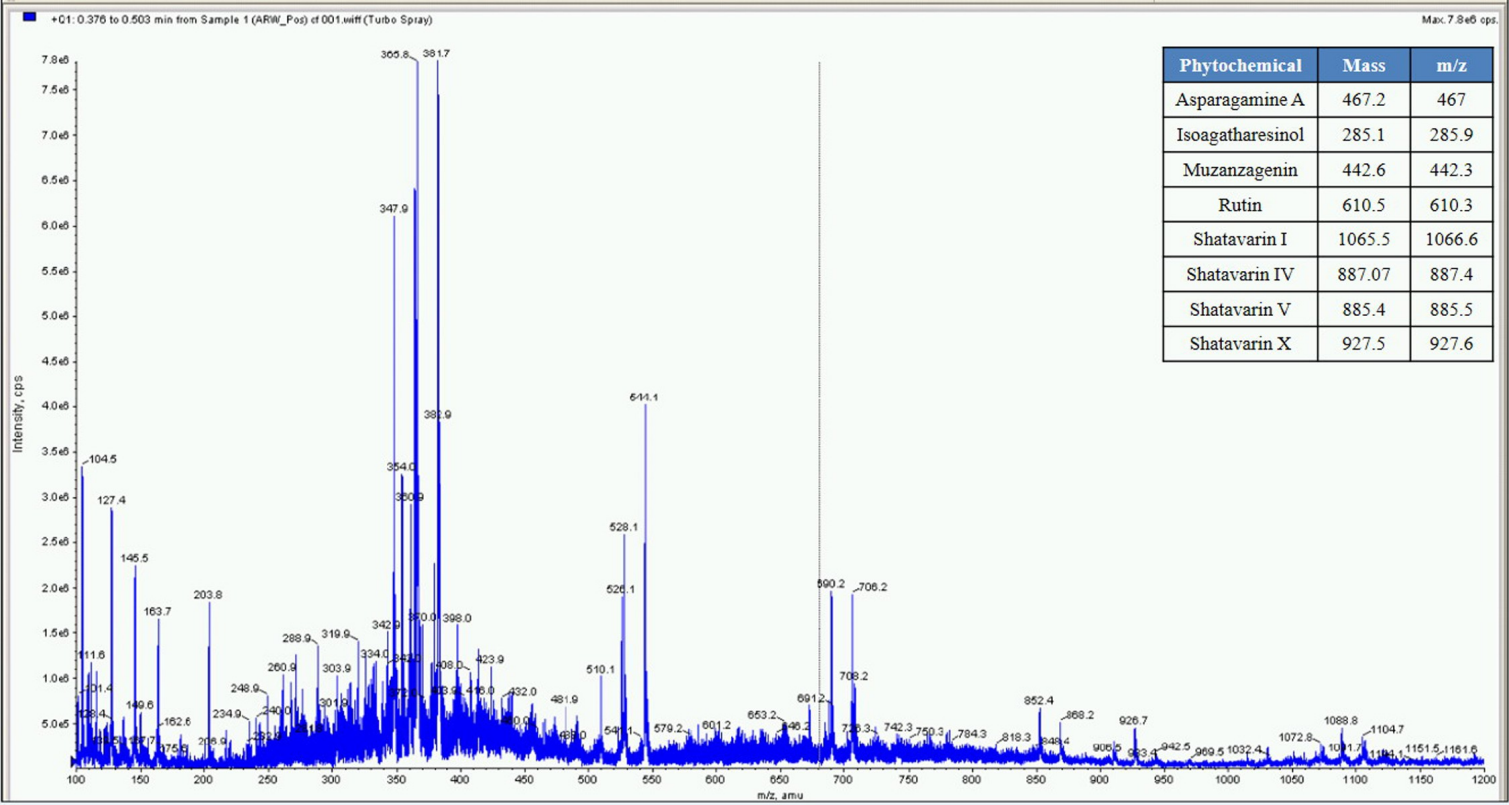
Mass spectrometer fingerprint of *Asparagus racemosus* water extract (ARW).

**Fig 9:**
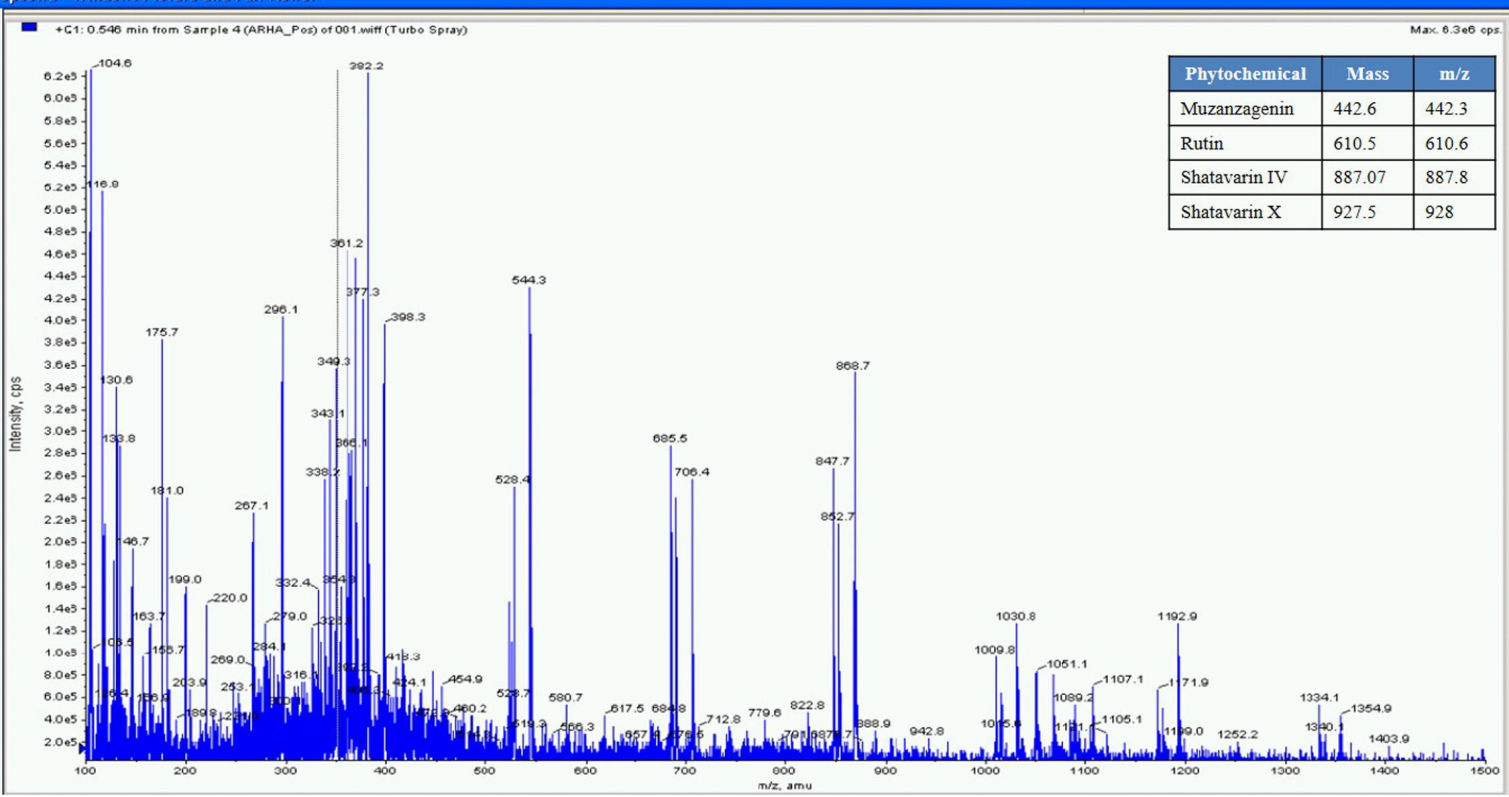
Mass spectrometer fingerprint of Asparagus racemosus hydro-alcoholic extract (ARHA).

The percent yield of hydro alcoholic extract of WS, TC, and AR were found to be 10%, 6.6%, and 30% in weight respectively. The percent yield of water extract of WS, TC, and AR were 12%, 7% and 20% respectively. The detailed phytochemical characterization and standardization was done as described in methods section and data has been provided in Figs 1–9. As per the current norms for phytochemical characterization we have quantified at least 4 phytoconstituents in all the test extracts, except *Asparagus racemosus* with the help of HPLC. Whereas MS were used for qualitative presence of phytoconstituents of the respective plant extracts. The extracts were also analysed for physiochemical properties, presence of impurities and microbial load as per pharmacopeial standards. The certificate of analyses showed that presence of impurities (heavy metals) and microbial load were within acceptable limit as per pharmacopeial standards.

UHPLC−PDA quantification WS, TC, and AR samples: The fingerprinting and quantitative analysis of a standardized WS, TC, and AR extracts is a robust quality control (QC) tool. The quantitative data represents the validated analysis of bioactive samples in this research, the six bioactive extracts. Many QC laboratories are equipped with UHPLC or HPLC instruments, which facilitate routine analysis. The quantification was done against linearity and calibration curve from UHPLC-PDA for WS, TC, and AR compounds by external standard method [42]. The WSHA and WSW samples confirmed the presence of eleven specific withanolides and withanosides in it. TCHA and TCW were quantified for six specific bioactive TC markers, which were found to present different concentrations. The ARHA and ARW were found to contain shatavarin-IV as major phytochemical in samples (**Table 1**).

**Table 1:** Quantification (*n* = 3) results (%) in WS, TC and AR hydroalcoholic and water extract.

**Sr.no.**	**Analytes**	**WSHA**	**WSW**
**1**	Withanoside-IV	0.4202	0.0999
**2**	Withanoside-VII	0.3180	0.0881
**3**	Viscosalactone-B	0.0348	0.0154
**4**	27-Hydroxywithanone	0.0002	0.0003
**5**	Dihydrowithaferin A	0.0042	0.0192
**6**	Withaferin-A	0.4117	0.1096
**7**	Withanoside- V	0.4511	0.0288
**8**	12- Deoxywithastramonolide	0.0716	0.0600
**9**	Withanolide-A	0.0974	0.0889
**10**	Withanone	0.0000	0.0002
**11**	Withanolide-B	0.0534	0.0093
**Sr.no.**	**Analytes**	**TCHA**	**TCW**
**1**	Cordifolioside A	0.0026	0.017
**2**	20-B-hydroxy ecdysone	0.6160	0.386
**3**	Tinosporaside	0.0043	0.028
**4**	8 Hydroxy tinosporide	0.655	0.121
**5**	Tinosporide	0.7817	0.066
**6**	Columbin	0.133	0.002
**Sr.no.**	**Analytes**	**ARHA**	**ARW**
**1**	Shatavarin-IV	0.0950	0.0596

**Note**: The text extracts are *Asparagus racemosus* water extract (ARW), *Asparagus racemosus* hydroalcoholic extract (ARHA), *Tinospora cordifolia* water extract (TCW), *Tinospora cordifolia* hydroalcoholic extract (TCHA), *Withania somnifera* water extract (WSW), *Withania somnifera* hydroalcoholic extract (WSHA).

Whereas MS based qualitative analysis helped to predict other additional phytoconstituents that may be present in these extracts. Briefly, a total of 31 phytoconstituents were identified from these plant extracts using HPLC and MS. The AR extract showed the presence of Asparagamine-A, Asparanin-A, Isoagatharesinol, Muzanzagenin, Rutin, Shatavarin-I, Shatavarin-IV, Shatavarin-IX, Shatavarin-VI, Shatavarin-VII, and Shatavarin-X. The TC extract showed the presence of 20-Hydroxy Ecdysone, Berberine, Columbamine, Columbin, Magnoflorine, Menisperine, Syringin, Tinocordiside, Tinosporaside and Tinosporide. WS extract showed the presence of 12-Deoxywithastramonolide, 27-Hydroxywithanone, Ashwagandhanolide, Withacoagin, Withaferin, Withanolide-A, Withanolide-B, Withanone, Withanoside-IV, Withanoside-V. The structures of 31 phytoconstituents are given as S3 Table in S1 File. These phytoconstituents were used for further studies.

### Network pharmacology reveals multiple associations of phytoconstituents with immune pathways

**Fig 10:**
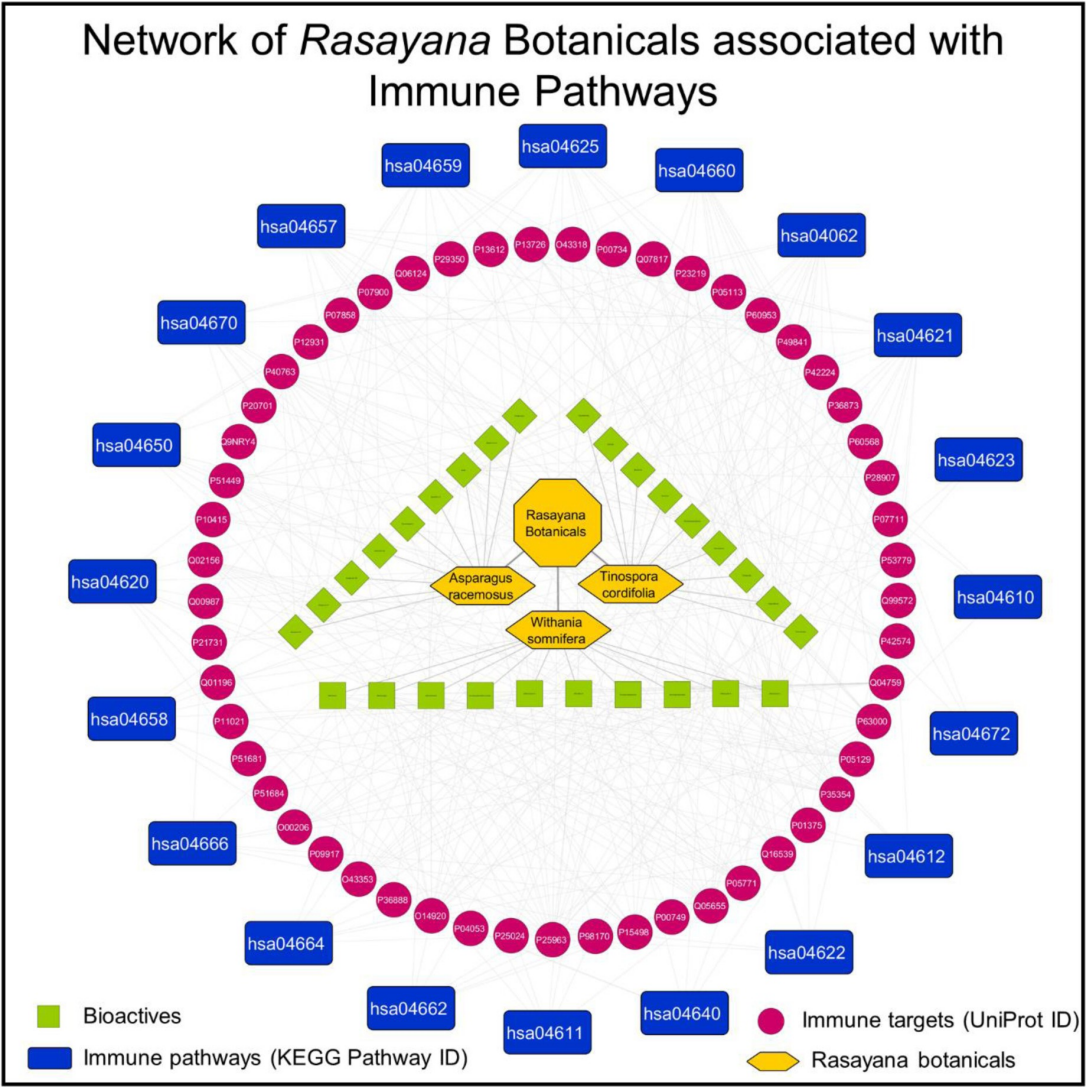
Network of Rasayana botanicals associated with immune pathways. Figure depicts potential mechanism of Rasayana botanicals to modulate several immune pathways through bioactive-target associations.

**Fig 11:**
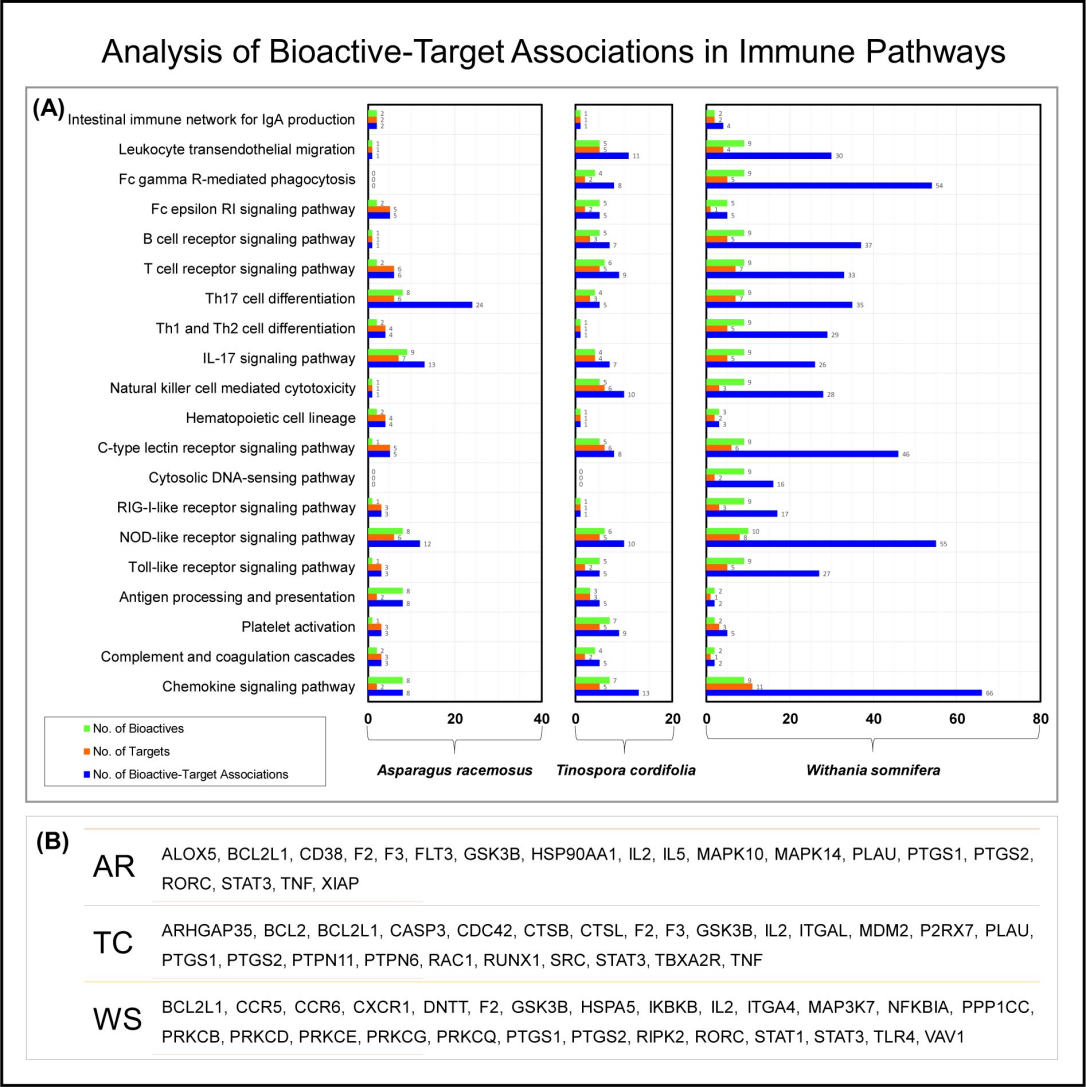
Analysis of bioactive-target associations in immune pathways. (A) The figure depicts the involvement of AR, TC, and WS in pathways of human immune system. The represented association is number of connecting combinations between bioactives and their targets in a particular pathway. (B) The table lists all immune targets of AR, TC, and WS.

*Rasayana* botanicals have immunomodulatory potential and help in rejuvenation of body homeodynamics [43, 44]. The bioactives i.e. Shatavarins of AR, Tinosporides of TC, and Withanolides of WS are known to play crucial role in immunomodulation [8, 20, 45]. According to traditional medicine or Ayurveda principles, the synergistic effect of bioactive combination in an extract strengthens physiological immunity superior than a single molecule [46]. To assess the immunomodulatory potential of the experimentally identified 31 phytoconstituents from AR, TC and WS a network pharmacology approach was followed [47]. As a first step, potential protein targets of bioactives were identified using the BindingDB server. The targets identified were mapped to known immune pathways that were extracted from the KEGG database and plotted using Cytoscape. The retrieved data showed total 29 bioactives from *Rasayana* botanicals associated with 306 unique human protein targets. Of these, 53 protein targets of 28 bioactives were found to be involved in 20 immune pathways referred as immune targets (Fig 10). The detailed mapping of phytoconstituents from each plant with targets and the number of associations reveals that bioactives from *Withania somnifera* show the largest number of associations (Fig 11A). The distribution of immune targets amongst *Rasayana* botanicals is shown Fig 11.

More specifically, we analysed the data to evaluate involvement of *Rasayana* botanicals in immune pathways by combinations of bioactives and targets in a particular immune pathway (Fig 11). The analysis showed involvement of AR in Th17 cell differentiation with 24 associations between 8 bioactives and 6 targets. AR was also found to be involved in IL-17 signalling with 13 associations between 9 bioactives and 7 targets. Data analysis showed association of TC with chemokine signalling with 13 associations between 7 bio-actives and 5 targets. TC was also found associated with few other pathways such as NOD-like receptor signalling, leukocyte trans-endothelial migration, and NK cell mediated cytotoxicity by moderate bioactive-target associations. The analysis also retrieved multimodal involvement of WS in immunomodulation through various pathways. It was found to be associated with chemokine signalling with 66 associations between 9 Withanolides and 11 immune targets. WS was also found to be associated with FC-gamma R-mediated phagocytosis and receptor signalling (NOD-like and c-type lectin) by 54, 55, and 46 bioactive-target associations. Furthermore, WS was also found to be associated with T cell differentiation, NK cell cytotoxicity and signalling pathways of IL-17, TCR and BCR etc. The multidimensional correlation with immune pathways underlines the importance of WS in managing the immunopathology of COVID-19 by improving T cell, B cell and NK cell function and hence anti-viral immunity.

AR, TC, and WS showed association with 18, 19, and 20 immune pathways through involvement of 19, 25, and, 27 immune targets respectively (Fig 11). The 7 common targets associated with all three *Rasayana* botanicals are Bcl-2-like protein 1 (*BCL2L1*), glycogen synthase kinase-3 beta (*GSK3B*), Interleukin-2 (*IL2*), prostaglandin G/H synthase 1 and 2 (*PTGS1* and *PTGS2*), prothrombin (*F2*), and signal transducer and activator of transcription 3 (*STAT3*). These immune targets are found to be involved in 11 different immune pathways including chemokine and specific receptor signalling (NOD-like, C-type lectin, BCR, and TCR), immune cell differentiation (Th1, Th2, and Th17), platelet activation and coagulation cascade, and intestinal IgA production. These results highlight the ability of Rasayana botanicals to modulate multiple immune pathways and have implications for immunomodulatory potential of these botanicals.

### Phytoconstituents show potential of antiviral activity

To identify if any of the 31 phytoconstituents might possess the potential for antiviral activity against SARS-CoV-2, they were docked to three important drug targets of the virus, the Spike protein, the Main Protease and the RNA dependent RNA polymerase. Molecular docking was performed using the protocol as described in the methods section.

### Several phytoconstituents are predicted to possess good affinity for the Main Protease (M^pro^)

**Fig 12:**
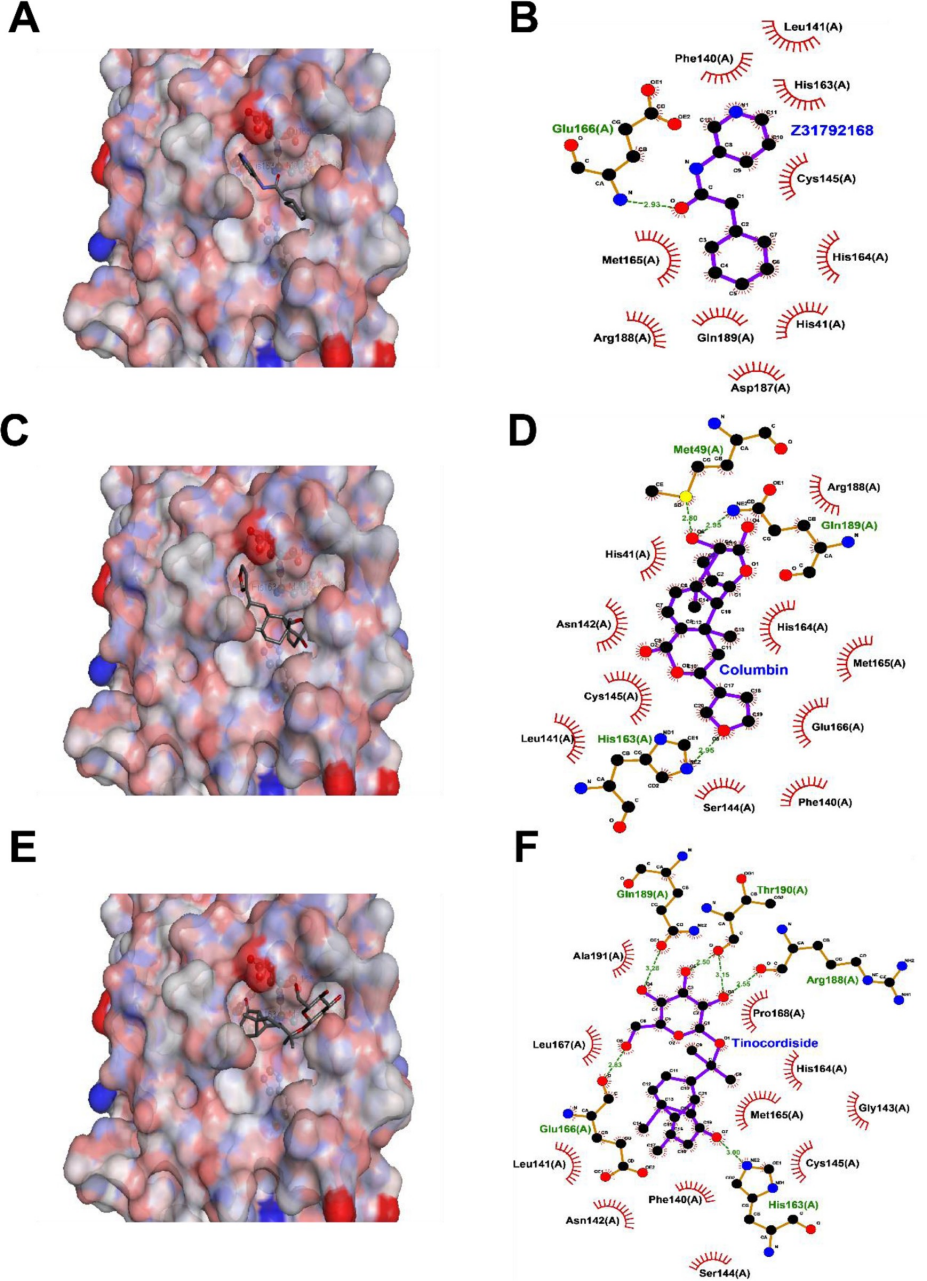
Interaction of (A and B) cyclohexyl-N-(3-pyridyl)acetamide (Z31792168), (C and D) Columbin and (E and F) Tinocordiside with M^pro^: In the left panel the protein is shown in a surface representation colored by atom while the ligand is shown in stick representation. The right panel is a 2D interaction plot of the receptor with the inhibitors.

There were 84 crystal structures of the Main Protease (M^pro^) in the PDB at the time of writing this manuscript. The largest share of the deposition is a series of M^pro^ crystal structures obtained by fragment screening. This indicates that the binding site of M^pro^ is druggable. The M^pro^ is known to be functional as a homodimer and has a heart-like shape. The protein has one active site per monomer. The active site contains the catalytic cysteine-Cys145, that performs the proteolysis reaction. The structure, PDB ID: 5R84, solved at a resolution of 1.83Å was chosen for this study. In this structure, the protease is co-crystallized with the fragment cyclohexyl-N-(3-pyridyl) acetamide (Z31792168) which is seen to bind in the S1-S3 pocket of the protease (Fig 12A). The S1 pocket is characterized with the presence of His163, Glu166, S2 with Cys145 and S3 is known as the aromatic wheel and includes Phe181 and His41. Thus, in our studies we placed the docking grid over the S1-S3 pocket. Initially, the co-crystallized ligand was separated and docked to check if Autodock was able to reproduce the binding mode. The docked pose showed an RMSD of 0.46 Å with the crystal structure pose and a docking score of -6.2 kcal/mol (Fig 12A and 12B). A detailed study of interactions revealed that the carbonyl group of Z31792168shows a H-bond with Glu166 in the S1 pocket. The cyclohexyl moiety of the ligand has hydrophobic interactions with Met165, His41, His164, Arg188 and Gln189 residue in the S3 pocket. The pyridine ring scaffold shows hydrophobic interactions with Phe140 and His163 in the S1 pocket. All these interactions are in line with the crystallographic pose.

Thereafter, 31 phytoconstituents were docked to the binding site. The results are compiled in Table 2. It is a frequent observation that compounds with a docking score better than -6 kcal/mol have a higher probability of being active *in vitro* and *in vivo* and hence use this score as a cutoff [48]. In our study we observed that, 18 out of 31 phytoconstituents have a docking score better than -6 kcal/mol. The best docking score -9.9 kcal/mol was observed for Ashwagandhanolide. Many other phytoconstituents like Withacoagin, Withaferin and Withanone are observed to have docking scores close to the -9 kcal/mol. However, a point to be noted is that some of these phytoconstituents are large molecules and hence the docking scores may appear to be inflated due to size and the proportionately larger number of interactions. Ligand efficiency is a measure of the activity corrected for the ligand size [49]. A calculation of the ligand efficiency indicates that the co-crystallized ligand, Z31792168, has a ligand efficiency of -0.4. Among the phytoconstituents, the highest ligand efficiency, -0.3, is observed for Columbin. Several other phytoconstituents have comparative ligand efficiency namely, Tinocordiside, Magnoflorine and Isoagatharesinol. Columbin and Tinocordiside not only have docking scores close to -8 kcal/mol but also have high ligand efficiencies. Similarly, Withacoagin, Withaferin, Withanolide-A, B and Withanone have good docking scores and ligand efficiencies. Interestingly, all compounds also follow Lipinski Rules which helps to distinguish between drug like and non-drug like molecules. A detailed study of interactions of M^pro^ with Columbin (Fig 12C and 12D) revealed that the tetrahydrofuran part of the ligand forms hydrogen bonds with His163 in the S1 pocket, while the hydroxyl and carbonyl groups form hydrogen bonds with Gln89 and electrostatic interactions with Met49. Further the ligand makes vdW interactions with several residues in the S2 and S3 pockets in a manner like Z31792168. A short simulation of this docked pose reveals that the ligand repositions but is stable in the S1-S3 binding pocket (S1 and S2 Figs in S1 File). The detailed interactions of Tinocordiside (Fig 12E and 12F) reveal that the hydroxyl groups of the β-glucose part of the ligand positioned in the S3 pocket of the protein form hydrogen bonds with the backbone of Arg188, Thr190, Glu166 and the sidechain of Gln189. The carbonyl group on the other side of the ligand forms hydrogen bonds with His 163 from the S1 pocket. Additionally, hydrophobic interactions are observed with amino acids Phe140, Asn142, His164, Met165 and Pro186.

**Table 2:** Docking results of the phytoconstituents from AR, TC and WS.

Ligand	Heavy atoms	Main Protease		Spike Glycoprotein (RBD)		RNA dependent RNA polymerase	
		Docking Score	Ligand efficiency	Docking Score	Ligand efficiency	Docking Score	Ligand efficiency
Asparagamine-A	28	-7.1	-0.25	-5.4	-0.19	-6.3	-0.22
Asparanin-A	52	-6.3	-0.12	-5.8	-0.11	-7.5	-0.14
Isoagatharesinol	21	-5.8	-0.28	-4.6	-0.22	-5.0	-0.24
Muzanzagenin	32	-7.6	-0.24	-7.1	-0.22	-9.3	-0.29
Rutin	43	-5.3	-0.12	-3.3	-0.08	-3.6	-0.08
Shatavarin-I	74	-1.1	-0.01	-1.7	-0.02	-3.3	-0.04
Shatavarin-IV	62	-2.7	-0.04	-6	-0.1	-6.3	-0.10
Shatavarin-IX	63	-0.6	-0.01	-5.4	-0.09	-6.7	-0.11
Shatavarin-VI	62	-4.9	-0.08	-4.6	-0.07	-7.5	-0.12
Shatavarin-VII	62	-6.6	-0.11	-5.7	-0.09	-6.3	-0.10
Shatavarin-X	66	-0.4	-0.01	-4	-0.06	-5.2	-0.08
20-HydroxyEcdysone	34	-6.4	-0.19	-4.8	-0.14	-5.7	-0.17
Berberine	25	-6.3	-0.25	-5	-0.2	-5.7	-0.23
Columbamine	25	-5.9	-0.23	-4.6	-0.18	-5.4	-0.22
Columbin	26	-7.9	-0.30	-5.8	-0.22	-6.8	-0.26
Magnoflorine	25	-7.0	-0.28	-5.2	-0.21	-6.6	-0.27
Menisperine	26	-6.9	-0.27	-5.2	-0.2	-6.2	-0.24
Syringin	26	-5.3	-0.20	-3	-0.11	-4.2	-0.16
Tinocordiside	28	-8.1	-0.29	-5.1	-0.18	-6.6	-0.24
Tinosporaside	35	-7.9	-0.23	-5.7	-0.16	-6.6	-0.19
Tinosporide	27	-4.3	-0.16	-4.6	-0.17	-4.4	-0.16
12-Deoxywithastramonolide	34	-8.1	-0.24	-6.9	-0.2	-7.9	-0.23
27-Hydroxywithanone	35	-8.6	-0.25	-7.6	-0.22	-7.6	-0.22
Ashwagandhanolide	69	-9.9	-0.14	-10	-0.14	-10.2	-0.15
Withacoagin	33	-8.8	-0.27	-7.6	-0.23	-8.8	-0.27
Withaferin	34	-8.8	-0.26	-6.9	-0.2	-8.5	-0.25
WithanolideA	34	-8.5	-0.25	-6.7	-0.2	-8.2	-0.24
WithanolideB	33	-8.3	-0.25	-7.4	-0.22	-8.9	-0.27
Withanone	34	-8.8	-0.26	-7.1	-0.21	-8.9	-0.26
WithanosideIV	55	-5.6	-0.10	-4.6	-0.08	-5.8	-0.11
WithanosideV	54	-6.1	-0.11	-5.1	-0.1	-6.0	-0.11

### Several phytoconstituents are also predicted to possess good affinity for the RNA dependent RNA polymerase (RdRp)

For the RdRp of SARS-CoV-2 the PDB ID: 6M71 was chosen for this study [34]. This is a cryoEM structure solved at a resolution of 2.9 Å. Residues 367 to 920 of the structure form the RdRp domain. The authors report that a structural comparison of theSARS-CoV-2RdRp with that of Poliovirus (PDB ID: 3OL6) and HCV indicates that the polymerase domain adopts a conserved structural architecture. The residues Arg553, Lys545 and Arg555 form the NTP entry channel while residues Asp760 and Asp761 coordinate divalent cations that stabilize the phosphate group (the cations are absent from PDB ID: 6M71 as it is an apo structure). We placed our grid over the entire RNA binding site based on the structure of Poliovirus RdRp that is co-crystallized with RNA.

**Fig 13:**
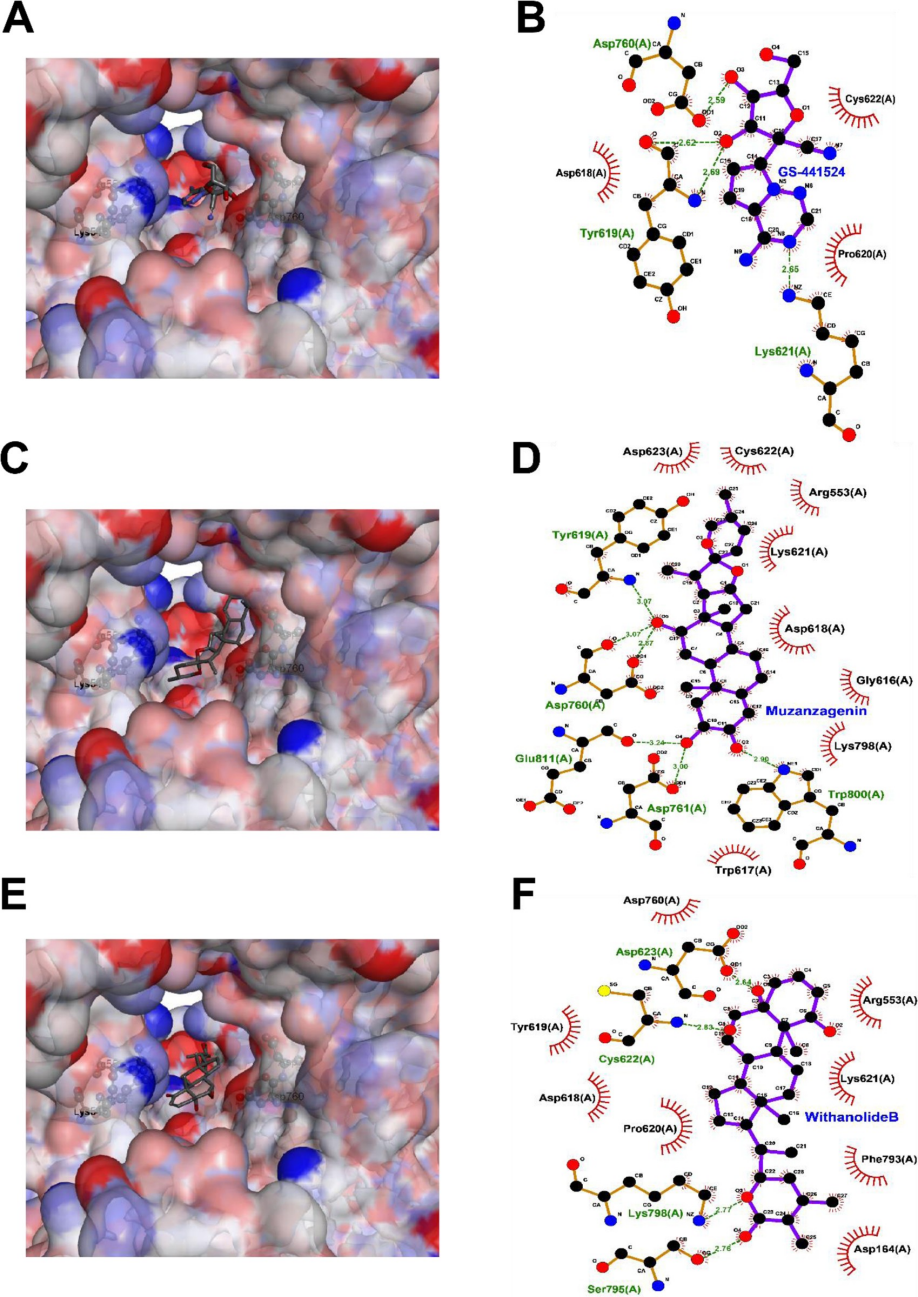
Interaction of (A and B) Remdesivir (C and D), Muzanzagenin and (E and F) Withanolide-B with RdRp. In the left panel the protein is shown in a surface representation colored by atom while the ligand is shown in stick representation. The right panel is a 2D interaction plot of the receptor with the inhibitors.

Remdesivir is an ATP analog and is being evaluated as a potential treatment for SARS-CoV-2 as an inhibitor of RdRp based on results obtained for MERS and SARS-CoV [50]. Remdesivir is a prodrug whose active metabolite is GS-441524 (PubChem CID: 44468216). GS-441524 was docked to the RNA binding site. The docked results (Fig 13A and 13B) indicate that the active metabolite forms hydrogen bonds with Asp760 which is an important active site residue and with Lys621 and Tyr619. The position of the active metabolite is that of the NTP entry channel. The docking score for the metabolite is -4.28 and the ligand efficiency is -0.2. Next, 31 phytoconstituents were docked to the binding site. The results are compiled in Table 2. Withanolide-B, Withacoagin, Withanone, Ashwagandhanolide and Muzanzagenin are predicted to possess docking scores ranging from ~ -9 to 10 kcal/mol. A total of twenty-one phytoconstituents have a docking score better than -6 kcal/mol. Calculation of ligand efficiency reveals that the top binders include Muzanzagenin, Withaolide-B, Withacoagin and Magnoflorine. There are sixteen compounds with ligand efficiency better than the active metabolite. However, it is to be noted that the GS-441524 is a chain terminating nucleotide analog, while these compounds would be pure blockers. The detailed interactions of Muzanzagenin with RdRp (Fig 13C and 13D) reveal two hydrogen bonds with the critical residues Asp760 and Asp761 apart from Trp800 and non-polar interactions over the NTP entry channel with residues Arg553 Lys621, Arg623. The binding pose and interactions of Withanolide-B (Fig 13E and 13F) also show interactions with the residues in NTP entry channel and the cation coordinating residues. In particular, hydrogen bonds are observed with side chains of residues Asp623, Ser795 and Ser795 and backbone of Cys622 while non-polar interactions are observed with Phe140, Leu141, Leu167, Ala191. A short simulation of this docked pose reveals that there is a slight repositioning of the ligand in the binding pocket (S1 and S3 Figs in S1 File).

### Phytoconstituents from WS are predicted to possess good affinity for the Spike protein

The entry of coronaviruses into cells is owing to the interaction between the clove shaped trimeric Spike viral protein with the human ACE2 receptors on the cell surface. Since this is the first and crucial point of contact between the viral protein with the human receptor this is a sought-after target for design of vaccine and therapeutics [51]. There are several structures of the SARS-CoV2 Spike protein with the human ACE2 protein. We chose the structure PDB ID: 6M17 for our docking studies [35]. This is a Single Particle cryoEM structure solved at a resolution of 2.9 Å. This structure represents the Receptor Binding Domain (RBD) of Spike protein with full length human ACE2. The Spike protein RBD interacts with the Peptidase Domain (PD) of the ACE2 in a 1:1 ratio. The interactions between the RBD and PD are mediated by residues Gln24, Lys417, Tyr453, Gln474, Phe486, Gln498, Thr500 and Asn501 of RBD. These residues are divided into three clusters, two clusters at each end and one in the center of the RBD. We placed the docking grid around all these residues to find molecules that could disrupt the contacts between RBD and PD.

**Fig 14:**
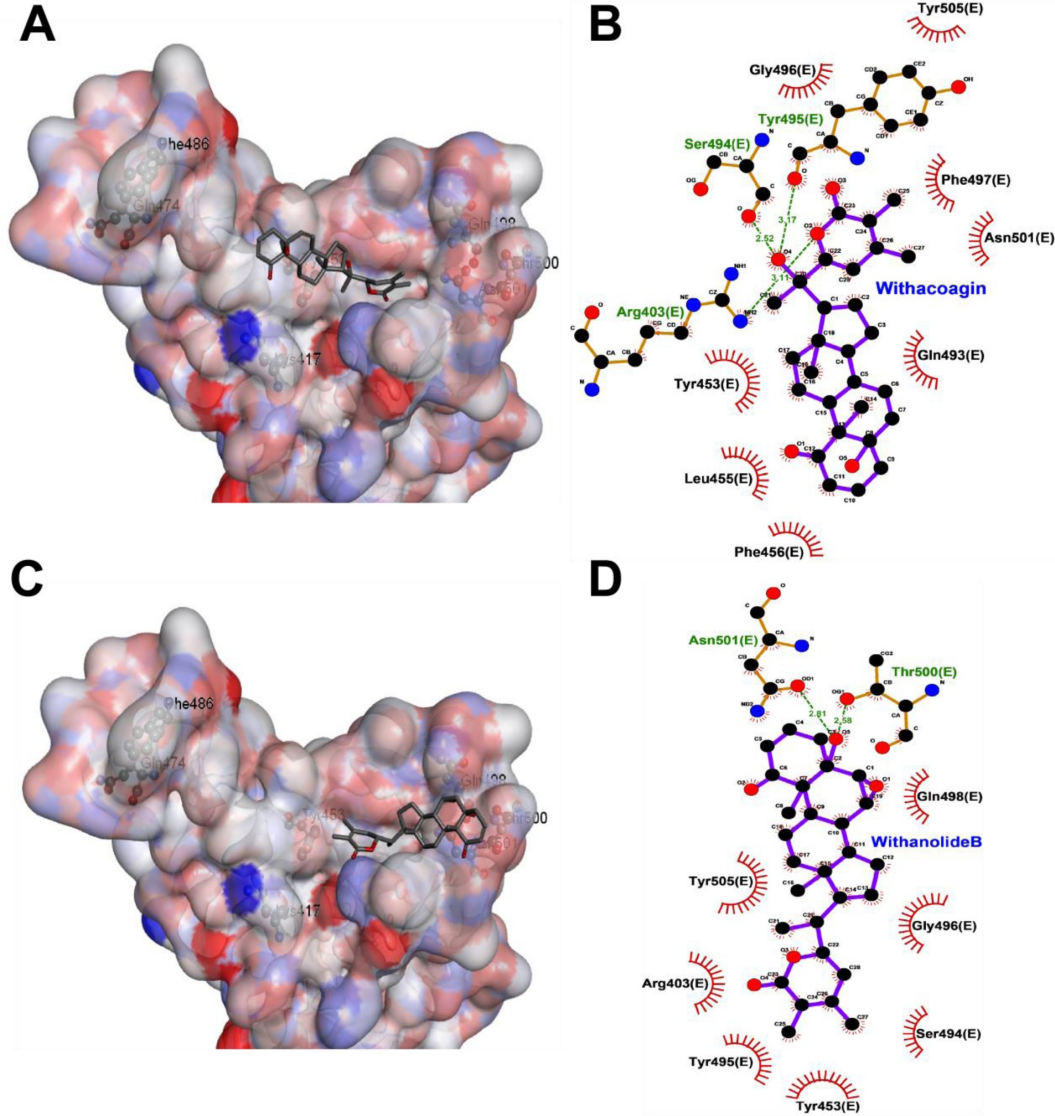
Interaction of (A and B) Withacoagin and (C and D) Withanolide-B with Spike protein: In the left panel the protein is shown in a surface representation colored by atom while the ligand is shown in stick representation. The right panel is a 2D interaction plot of the receptor with the inhibitors.

The docking results with 31 phytoconstituents reveal that the compounds bind in various areas of the interface region of RBD. The top scoring compound is Ashwagandhanolide with a docking score of -10 kcal/mol. Ten phytoconstituents have a docking score better than -6 kcal/mol. Withacoagin, 27-Hydroxywithanone and Withanolide-B are predicted to have docking scores of -7.6, -7.6 and -7.4 kcal/mol respectively. The ranking based on ligand efficiency places Withacoagin at the top with a value of -0.23. Several compounds including Withanolide-B have a ligand efficiency of -0.22. Detailed analysis of the top docked pose of Withacoagin (Fig 14A and 14B) indicates that it binds over middle and terminal cluster of residues that interact with PD. The ligands form hydrogen bonds with Ser494, Tyr495 and Arg403 and vdW interactions with Phe497, Asn501, Gln493, Tyr453, Leu455 and Phe456. A short simulation of this docked pose reveals that the ligand repositions in the binding pocket (S1 and S4 Figs in S1 File). Withanolide-B only interacts with the terminal cluster of residues and shows hydrogen bonds with Thr500 and Asn501 and non-polar interactions with Tyr453, Ser494, Tyr495, Arg403, Tyr505, Gly496 and Gln498 (Fig 14C and 14D).

### Predicting herb-drug interactions

The Swiss-ADME, cheminformatics platform was used to predict pharmacokinetics and drug likeliness potential of the 31 phytoconstituents. Bioavailability RADAR analysis showed, 9 phytoconstituents of TC are orally bioavailable (S5 Fig in S1 File). Out of 11 phytochemicals of AR only Muzanzagenin and Asparagamine-A were found to be orally bioavailable. For WS, except Ashwagandhanolide, Withanoside-V and Withanoside-IV all other analyzed phytochemicals were found to be orally bioavailable. The assessment of drug likeness indicates that Muzanzagenin and Asparagamine-A from AR, all analyzed phytochemicals of TC except 20-β-Hydroxy Ecdysone and Tinosporaside and all analyzed phytochemicals of WS except Ashwagandhanolide, Withanoside-V and Withanoside-IV have drug-like properties (Table 3). Further, apart from Syringin, Asparagamine-A and Isoagatharesinol all the phytoconstituents are predicted to be a substrate of P-gp transporter (Table 3). Muzanzagenin, Asparagamine-A from AR and Columbamine, Berberine, Magnoflorine, Menisperine from TC showed ability to penetrate the blood brain barrier (BBB). Log Kp (Kp in cm/s) is skin permeation coefficient that assesses ability of test molecule to permeate through skin. Large negative value indicates lesser skin permeability. This is directly correlated with molecular size and lipophilicity of compounds [52]. Log Kp values shown by all analyzed phytochemicals indicate lower skin permeability. An important aspect of developing therapeutics or their adjuvants is whether they are metabolized by Cytochromes thus reducing their efficacy or potentially interfering with concomitant therapy. Predictions showed that none of the phytochemicals of AR except, Isoagatharesinol have the potential to inhibit any of the major CYP isoforms (Table 3). Few TC phytochemicals may act as inhibitor of CYP1A2, CYP2D6 and CYP3A4 whereas few WS phytochemical may inhibit CYP2C9. These properties need to be considered during phytopharmaceutical development. However, these predictions may not be hold true for extracts as they contain multiple phytoconstituents because of which several properties might change.

**Table 3:** *In silico* pharmacokinetic analysis of phytoconstituents from AR, TC, and WS.

Herbs	Phytoconstituents	Predicted oral bioavailability^§^	Predicted Drug-likeness^¥^	Pharmacokinetics				
				CYP^£^	P-gp substrate	BBB permeability	GI absorption	Log Kp (cm/s)
Asparagus racemosus	Muzanzagenin	Orally bioavailable	Y	N	Y	Y	High	-6.94
	Asparanin A	Not orally bioavailable because polarity, solubility and size axes lie outside pink region	N	N	Y	N	Low	-8.48
	Asparagamine A	Orally bioavailable	Y	N	N	Y	High	-6.95
	Isoagatharesinol	Not orally bioavailable because saturation parameter does not lie within pink area	Y	CYP2D6, CYP3A4 only	N	N	High	-6.43
	Rutin	Not orally bioavailable because of higher molecular weight and polarity	N	N	Y	N	Low	-10.26
	Shatavarin I	Not orally bioavailable because of higher polarity, molecular weight and flexibility	N	N	Y	N	Low	-13.47
	Shatavarin IV	Not orally bioavailable because of higher molecular weight and polarity	N	N	Y	N	Low	-10.53
	Shatavarin VI	Not orally bioavailable because of higher molecular weight and polarity	N	N	Y	N	Low	-10.53
	Shatavarin VII	Not orally bioavailable because of higher molecular weight and polarity	N	N	Y	N	Low	-10.97
	Shatavarin IX	Not orally bioavailable because of higher molecular weight and polarity	N	N	Y	N	Low	-10.99
	Shatavarin X	Not orally bioavailable because of higher polarity, molecular weight and flexibility	N	N	Y	N	Low	-11.23
*Tinosporacordifolia*	Berberine	Orally bioavailable	Y	CYP1A2, CYP2D6, CYP3A4 only	Y	Y	High	-5.78
	Columbamine	Orally bioavailable	Y		Y	Y	High	-5.94
	Menisperine	Orally bioavailable	Y		Y	Y	High	-6.30
	Magnoflorine	Orally bioavailable	Y	CYP1A2, CYP3A4 only	Y	Y	High	-6.44
	Tinopsoraside	Not orally bioavailable because of higher molecular weight and polarity	N	N	Y	N	Low	-9.13
	Tinosporide	Orally bioavailable	Y	CYP2D6 only	Y	N	High	-7.64
	Tinocordiside	Orally bioavailable	Y	N	Y	N	High	-8.56
	Syringin	Orally bioavailable	Y	N	N	N	Low	-9.50
	Columbin	Orally bioavailable	Y	N	Y	N	High	-6.95
	20-β-Hydroxy Ecdysone	Orally bioavailable	N	N	Y	N	High	-8.91
*Withaniasomnifera*	Withacoagin	Orally bioavailable	Y	CYP2C9 only	Y	N	High	-6.29
	Withanolide B	Orally bioavailable	Y		Y	N	High	-5.76
	Withastramonolide-12-Deoxy	Orally bioavailable	Y		Y	N	High	-6.35
	Withanoside IV	Not orally bioavailable because of higher molecular weight and polarity	N	N	Y	N	Low	-10.37
	Withanolide A	Orally bioavailable	Y	N	Y	N	High	-6.86
	Withanone	Orally bioavailable	Y	N	Y	N	High	-7.01
	Withaferin A	Orally bioavailable	Y	N	Y	N	High	-6.45
	27-hydroxy Withanone	Orally bioavailable	Y	N	Y	N	High	-7.60
	Withanoside V	Not orally bioavailable because of higher molecular weight and polarity	N	N	Y	N	Low	-9.79
	Ashwagandhanolide	Not orally bioavailable as four axes namely of lipophilicity, size, polarity and solubility lies outside pink area	N	N	Y	N	Low	-6.95

**Note:** CYP–Cytochrome P450, are the Phase-I drug metabolising enzymes responsible for >95% xenobiotic’s metabolism; P-gp–P-glycoprotein is key efflux drug transporter; BBB–blood brain barrier plays a crucial role in prevention of drug transport into the brain; whereas, Kp explains skin permeability coefficient. Kp is linearly correlated with lipophilicity and molecular size. The more negative the log

Kp (with Kp in cm/s), the less skin permeant is the molecule.

§ Predicted oral bioavailability from RADAR graph (Supplementary file: S1 Appendix)

^£^ Predicted CYP Inhibitionfor CYP1A2, CYP2C9, CYP2C19, CYP2D6and CYP3A4

^**¥**^Predicted Drug-likeness (Results of Lipinski, Ghose, Veber, Egan and Muegge rules)

COVID-19 pathogenesis mainly involves respiratory system and afterwards it leads to multiple organ failure based on patient related factors: sex, age, disease, individualization (PRF: SADI). As discussed earlier, diabetic, obese, asthmatic, geriatric, hypertensive population is more prone for COVID-19 [53]. These associated comorbidities of COVID-19 are being continually treated along with anti-SARS-COV-2 therapy. Here, we propose the use of *Rasayana* botanicals for COVID-19 prophylaxis (both pre and post COVID-19) as well as anti-SARS COV-2 activity. This can lead to the chances of HDIs, which may be harmful/beneficial/fatal [40]. Therefore, with the help of Swiss-ADME data (Table 3) and available published literature (S2 Table in S1 File) the probable pharmacokinetic HDI were explored. This showed that, whole plant extracts of these botanicals (especially those that are prepared according to the Ayurvedic procedure) do not inhibit main CYP isoforms such as, CYP1A2, CYP, CYP2D6 and CYP3A4. Various extracts of AR, TC and WS have IC_50_ values >100 μg/mL for these CYP isoforms (S2 Table in S1 File). This indicates that at higher concentrations (generally more than therapeutic dose), these extracts may produce pharmacokinetic HDI *in vivo* [54]. Based on the above results, these phytoconstituents or *Rasayana* botanicals may produce beneficial pharmacokinetic-pharmacodynamic interactions *in vivo*, with the anti-viral and disease-modifying drugs that are currently being prescribed for COVID-19.

## Discussion

**Fig 15:**
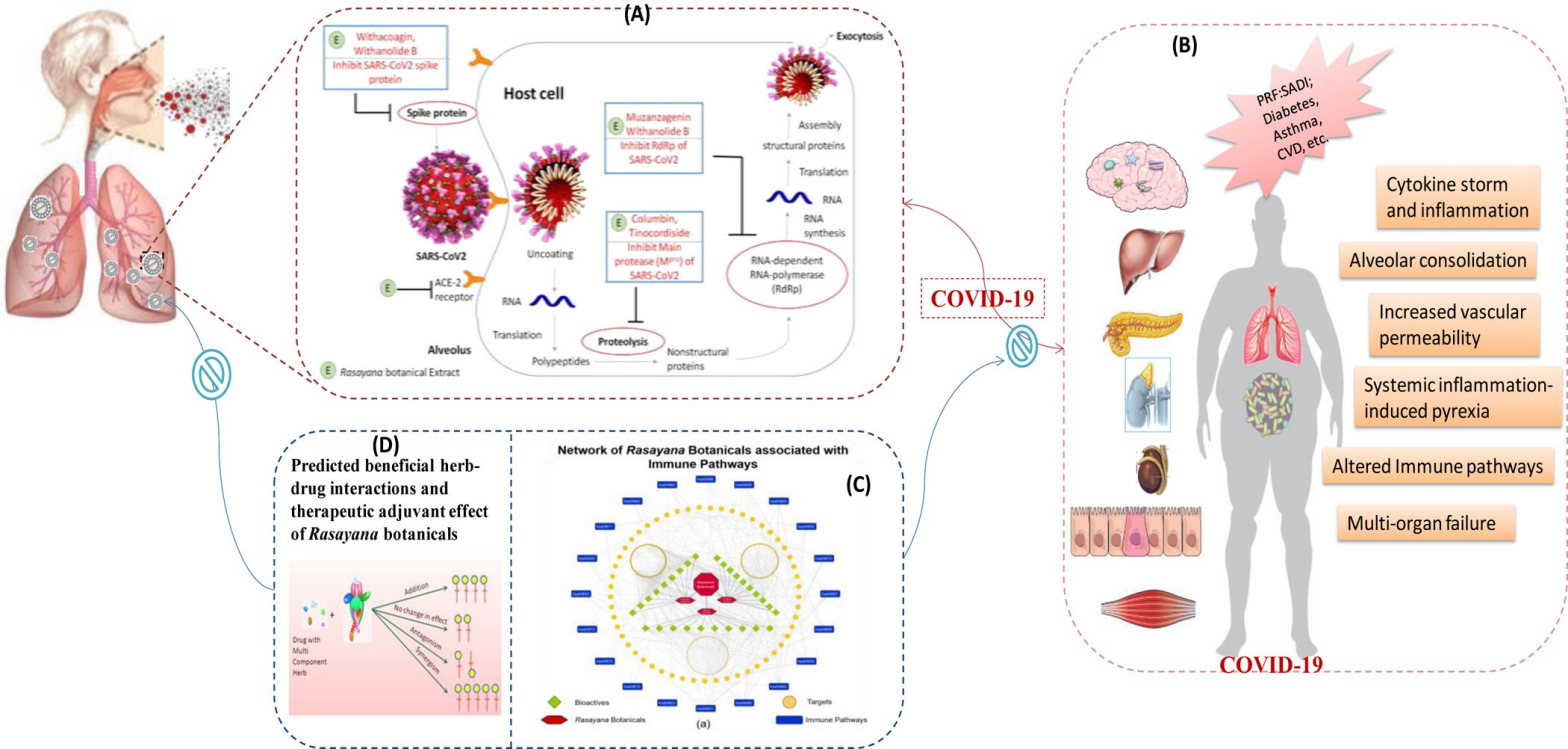
Predicted role of Ayurvedic Rasayana botanicals in the management of COVID-19. Once the SARS-CoV-2 virus passes the respiratory tract, it enters the lung cells with the help of its Spike protein coupled with ACE-2 receptors (these are present almost all over the body enabling its spread to multiple organs and their failure in later stages of the disease). Rasayana botanical extract constituents (E), Withacoagin and Withanolide B may inhibit COVID-19 entry by disrupting interactions between viral spike protein and host ACE-2 receptor. Rasayana botanicals may inhibit viral replication through inhibition of coronavirus main protease (M^pro^) and RNA-dependent RNA-polymerase (RdRp) Part A: The M^pro^ is can be inhibited by Columbin and Tinocordiside. RdRp can be inhibited by Muzanzagenin and Withanolide-B thereby inhibiting RNA synthesis of SARS-CoV-2. This may lead to its life cycle arrest. Part-(B): The patient related predisposition factors such as sex, age, diseases, individualization (PRF:SADI) and COVID-19 pathophysiology involved in the multiple organ failure and death. Part (C): the multi-targeted immunomodulatory and adaptogenic potential of Rasayana botanicals as predicted by network pharmacology approach. Part-(D) shows higher probability for the beneficial pharmacokinetic- pharmacodynamic herb-drug interactions 37 if co-prescribed with WHO Solidarity trial drugs and drugs for COVID-19 associated comorbidities. It is interesting to note that above mentioned phytoconstituents are predicted to have good docking score, ligand efficiency, oral bioavailability, and drug likeliness. This makes them potential molecules for rapid drug discovery and development based on multi-targeted and reverse pharmacology approach for COVID-19 management. However, these in silico predictions need in vitro–in vivo validation.

The understanding of pathophysiology of COVID-19 is emerging with increasing prevalence over the globe [55]. A wide variation in the patient population ranging from asymptomatic, to mild or moderate cases and severe cases (some showing relapse) is reported. Severe infections of SARS-CoV-2 lead to mortality due to severe acute respiratory syndrome accompanied with hypoxia followed by organ failure [6]. In general, we need to have drugs that are best prophylactic (pre and post COVID-19), immunomodulatory and adaptogenic in nature along with anti-SARS-CoV-2 action. Using *in silico* approaches, the present study shows that the selected *Rasayana* botanicals may have these all the actions and may be effective for management of COVID-19 (Fig 15). In the following sections we discuss in detail how these *Rasayana* botanicals and their phytoconstituents can be potential therapeutic adjuvants for the effective management of COVID-19.

### Immune-bolstering activity

SARS-CoV-2 attacks pulmonary pneumocytes in alveolar sac for viral replication [7]. Typically, the immune response to viruses involve cytotoxic T-cells. The viral-infected host cells are recognised through binding of T-cell receptors (TCR) to MHC-I molecules. In case of viruses that escape from TCR recognition, physiological immune surveillance employs NK cells to eliminate virus infected cells. Cytotoxicity triggered by cytokines and other cytotoxic mediators induce infected cells to undergo apoptosis [56]. Our analysis showed that, Withanolides of WS may augment TCR signalling pathway by modulating NF-kappa-β regulators and theta type protein kinase C (PK-C). Withanolides may also involve in NK cell mediated cytotoxicity by modulating beta and gamma type PK-C. Rutin of AR and 20-hydroxyecdysone of TC may also help in destroying infected cells by regulating cytotoxicity mediator TNF-α. Magnoflorine and Menisperine of TC could modulate apoptosis through caspase proteins. They may involve in mediating innate immunity by regulating proteolytic cascade of complement system through coagulation factors. Shatavarins of AR were predicted to modulate antigen processing and presentation through heat shock proteins. Withanolides may interact with integrin protein and interleukins to intensify non-inflammatory intestinal IgA secretion to neutralize virus. our It has also been reported that Withanone and Withaferin A may disrupt interactions between viral S-Protein and host receptor angiotensin converting enzyme (ACE) by binding to it [22]. Our docking studies also reveal that Withacoagin, 27-Hydroxywithanone and Withanolide-B have the potential to perform the same activity.

### Cytokine storm and inflammation

If the body’s defence system or prophylactic treatment fails to control the viral entry and its clearance, the body activates strong inflammatory response leading to cytokine storm. Briefly, the internalization of SARS CoV-2 eventually leads to secretion of large quantities of proinflammatory cytokines [57]. This leads to engagement of immune cells at the infected site. The present data indicates potential of WS to interfere with chemokine signalling through beta and delta type PK-C and chemokine receptors. Similarly, IL-17 plays crucial role in acute and chronic inflammatory responses. The IL-17 signalling can be mitigated predominantly through heat shock protein by AR, glycogen synthase by TC, and prostaglandin synthase by WS. AR derived saponins and TC extract showed anti-inflammatory property by modulating pro-inflammatory cytokines along with other inflammatory modulators [58, 59]. WS extract also modulated cytokine expressions by inhibiting MAPK/NF‑κB pathway [60]. This pathogenic reaction may become more complicated in co-morbidities like diabetes, asthma, hypertension, and obesity where inflammasome is already present [61]. This further activates this inflammatory cascade and COVID-19 progression. It has been reported that the *Rasayana* botanicals containing phytoconstituents like Withaferin-A have an ability to treat the inflammasome [19, 62–64].

### Increased vascular permeability

The macrophage-mediated inflammatory cytokines cause contraction of endothelial cells of blood vessels which leads to increase in vascular permeability [65]. This increases migration of immune cells to occupy alveolar space. The proteins involved in trans-endothelial leukocyte migration are predicted to be modulate by Withanolides through our analysis. Integrin alpha-L (ITGAL) is one of the factor involved in trans-endothelial migration of leukocytes associated with COVID-19 pathogenesis [66]. Present data mining suggested ITGAL as putative target of 20-hydroxyecdysonefrom TC. Withaferin-A may inhibit and down-regulate vascular permeability factor i.e. VEGF [67, 68]and protects vascular barrier integrity inhibiting hyperpermeability induced by high-mobility group box 1 protein [69]. WS aqueous extract may inhibit histamine mediated endothelial contraction to avoid venular intercellular gaps [70, 71].

### Alveolar consolidation

The engagement of immune cells and extent of cytokine secretion consolidate alveolar space. Production of surfactants by pneumocytes intensifies severity leading to dyspnoea or shortness of breath [72]. Withaferin A reduced accumulation of M1 type macrophages characterized by IL-6 and TNF-α [73]. The *Rasayana* botanicals affect physiological inflammatory response by downregulating proinflammatory mediators. This is accompanied with preventing immune cell accumulation at infected site i.e. alveolar space [74]. Therefore, *Rasayana* botanicals may evade alveolar consolidation keeping pneumocytes engaged in O_2_ transport as usual.

### Systemic inflammation-induced pyrexia

In COVID 19, the cytokine storm (CS) results in excessive and uncontrolled release of pro-inflammatory mediators and cytokines. Clinically, it commonly presents as systemic inflammation and these inflammatory mediators act as a messengers to hypothalamus to increase body temperature through prostaglandin secretion [75]. Few studies have suggested that Withaferin-A may circumvent pyrexia by downregulating COX-2 expression with simultaneous decrease in prostaglandin production [76, 77]. The newer understanding of SARS CoV2 pathogenesis is coming out gradually. Recent computational evidence has suggested, haemoglobin disassociation andiron imbalance by SARS-CoV-2 infection. This was supported by clinical observations of reduction in haemoglobin and increased erythrocyte sedimentation rate. This induces hypoxia leading to organ failure [78]. A systematic *in vivo* study of TC extract showed significant increase in levels of haemoglobin and erythrocyte count to maintain systemic iron homeostasis [79]. WS showed prophylactic effect in pulmonary hypertension characterised by hypoxia and inflammation [80]. The connection of hypoxia and inflammatory signals is well established throughHIF1α gene [81]. Asparagus polysaccharides potentially inhibited HIF1α signalling which might ameliorate fatal systemic inflammation [82]. TC also exhibited anti-inflammatory effects in systemic models significantly [83]. Our data analysis also suggests probable action of AR, TC, and, WS in hematopoietic cell lineage through multiple protein targets involving integrin alpha-4 and CD38. It has now been clearly established that the adverse clinical outcomes in COVID-19 patients is associated with elevated IL-6 levels [84]. A case report using Tocilizumab(an anti-IL6 receptor antibody) defined an approach of mitigating risk of disease progression to target IL-6 [85, 86]. WS aqueous extract attenuatedIL-6 in arthritis model to reduce inflammatory response. Active components of WS (Withanone and Withaferin-A) and AR (Shatavarin-IV) also inhibited production of IL-6 in *in vitro* system. It has also been reported that aqueous extract of TC protects against inflammation associated anaemia by modulating hepcidin expression, IL-6 and other pro-inflammatory cytokine cascade [79]. There is a clinical evidence showing immunocompromised patients are at high risk of developing fatal respiratory syndrome [87, 88]. In view of this, immunomodulating drugs are being considered beneficial for COVID-19 management [89]. The Ayurveda-based *Rasayana* botanicals are well known for their immunomodulatory activities [90]. The adaptogenic and regenerative properties of *Rasayana* botanicals help to maintain physiological homeostasis [91]. The effects of improving antibody titre, modulating systemic Th1/Th2 immunity underline the potential of AR as an immunoadjuvant [92]. TC and its phytoconstituents are capable immunomodulators through their multimodal actions [23, 93]. The significant immune-boosting potential of WS in several model systems makes it one of the best possible therapeutic adjuvants from traditional medicine [20, 94, 95].

### Predicted herb-drug interactions

COVID-19 is a viral pandemic disease with no specific cure available so far. Few drugs and drug combinations are still under investigation for their efficacy in managing this fatal infection [96, 97]. Current treatment involves combination of previously available antiviral agents [98]. COVID-19 patients having co-morbidities like T2DM, hypertension, asthma, obesity, etc. are also being treated with these drugs along with their ongoing prescriptions [4]. Along with modern therapeutic agents, patients are also being treated with drugs from traditional medicinal system like Ayurveda [40, 99]. Thus, while using such diverse treatment regime, there is need for designing and executing detailed DDI (drug-drug interactions) and HDI (herb-drug interaction) studies for proposing safe, efficacious and beneficial combinations. DDI and HDI can be predicted by PK-PD pathways (with special focus on drug metabolising enzymes and transporters) of particular drug. Cytochrome P450 system (CYP’s) are major enzymes involved in catalysing biotransformation of administered drugs. CYP1A2, CYP3A4,CYP2C9 and CYP2D6 are involved in metabolism of around 80–90% of drugs [100]. Pharmacokinetic profiles of plant extracts considered in this study are not well established [101]. Data obtained from predictive web-based tools to evaluate the herb-drug PK-PD interactions analysis shows some phytochemicals may or may not inhibit main drug metabolizing enzymes (Table 3). This inference is also being supported by published *in vitro* studies on human liver microsomes using whole plant extracts of AR, TC, and WS (S2 Table in S1 File). IC_50_ values for all the extracts are > 100 μg/mL which seems to be a higher concentration than used *in vivo* [54, 102] (S4 Table in S1 File). Thus, we speculate that *in vitro* studies on human liver microsomes using whole plant extracts of AR, TC, and WS may not show any inhibition of these main CYP isoforms. However, there is a need to explore *in vitro—in vivo* HDI for rationalizing their use in pharmacotherapeutic management of COVID-19 [103]. We also recommend that HDI studies should be planned to focus on drugs used in the management COVID-19 and its associated comorbidities. The S5 Table in S1 File includes pharmacokinetic data of WHO Solidarity trial drugs and prescribed drugs (representative) for COVID-19 associated comorbidities (e.g. hypertension, asthma and T2DM). This data shows that, there is overlap for drug metabolizing enzymes especially among the allopathic drugs, indicating possible risky DDIs. Therefore, careful clinical validation is required. For instance, WHO Solidarity trial drugs (Remdesivir; Lopinavir/Ritonavir; Lopinavir/Ritonavir with Interferon beta-1a; and Chloroquine or Hydroxychloroquine) are being metabolized by CYP1A2, CYP3A4, CYP2C9, and/or CYP2D6 (S4 Table in S1 File). The commonly prescribed drugs for hypertension (Propranolol, Metoprolol, Telmisartan, Losartan) and T2DM (Glimepiride, and Pioglitazone) shares the same drug metabolizing enzymes (CYP1A2, CYP3A4, CYP2C9, and CYP2D6). This raises the concern for careful pharmacotherapeutic management. On the other hand, it can be predicted that there are very less chances of pharmacokinetic mediated HDI with the studied *Rasayana* botanicals as no such overlap has been found in our *in-silico* studies as well as published *in vitro data*.

In this study we explored the potential of Ayurveda based Rasayana drugs in COVID 19 management using *in silico* approaches and propose a library of phytoconstituents with the potential to be developed as phytopharmaceuticals. Appropriate pharmaceutics developability assessment needs to be done if any of these phytoconstituents are to be considered for phytopharmaceutical development. The *in-silico* pharmacokinetic data also shows that, Muzanzagenin is the main drug-like molecule from AR and it has also showed docking score better than -6.0 kcal/mol against all three protein targets of SARS-CoV-2. Majority of the key phytoconstituents show good oral bioavailability. All phytochemicals of TC were found to be drug-like substances. In case of WS phytochemicals, except Ashwagandhanolide, Withanoside V and Withanoside IV all were found to be drug-like molecules. Though, Ashwagandhanolide has shown highest docking score for all 3 SARS-CoV-2 protein targets its drug-likeness is an issue of concern and needs further detail investigations. On the other hand, the network pharmacology and docking data showed that there are very high chances of beneficial pharmacodynamic HDI for the effective pharmacotherapeutic management of COVID-19. At the same time, we cannot forget that the drug concentration plays the important role in both *in-vitro* and *in vivo* studies, therefore effective and safe clinical extrapolation based on experimentally validated data are warranted.

In conclusion, this study provides potential phytoconstituents for clinical application of Rasayana botanicals in prophylaxis because of their potential in inhibiting the replication of SARS-CoV-2. These botanicals can also be used as adjunct or mainstream treatment for COVID-19 when the disease is manifested with its symptom. The activities on immune mechanisms provide a sound logic for use of these botanicals in treatment. Some of the phytoconstituents have a possible role in arresting disease progression and preventing organ failure by reducing inflammatory responses. The adjunct use of these botanicals poses another important question of herb drug interactions which requires detailed experimental and clinical evidence.

## Supporting information

S1 File (DOCX)

S1 Data (XLSX)
