# Supplementary material for: Ayurveda botanicals in COVID-19 management: An in silico multi-target approach
Source: PLoS One. 2021 Jun 11;16(6):e0248479. doi: 10.1371/journal.pone.0248479 (PMC8195371; doi:10.1371/journal.pone.0248479)
Supplement: S1 File — (DOCX) [file pone.0248479.s001.docx]

**S1 File.**

**S1 Table: Solubility details of test materials in different solvents**

| **Sr. No.** | **Solvent** | **Type of Extract** | **Inference** |
| --- | --- | --- | --- |
|  | Distilled water | ARW | Freely soluble |
|  | Distilled water | ARHA | Freely soluble |
|  | Distilled water | TCW | Very Slightly Soluble |
|  | Distilled water | TCHA | Very slightly soluble |
|  | Distilled water | WSW | Practically insoluble |
|  | Distilled water | WSHA | Practically insoluble |
|  | 70% ethanol | ARW | Freely soluble |
|  | 70% ethanol | ARHA | Freely soluble |
|  | 70% ethanol | TCW | Practically insoluble |
|  | 70% ethanol | TCHA | Very slightly soluble |
|  | 70% ethanol | WSW | Practically insoluble |
|  | 70% ethanol | WSHA | Very slightly soluble |

The table shows solubility of test extracts in different solvents (water, and 70% ethanol). The extracts are *Asparagus racemosus* waterextract (ARW), *Asparagus racemosus* hydroalcoholic extract (ARHA), *Tinospora cordifolia* waterextract (TCW)*, Tinospora cordifolia* hydroalcoholic extract (TCHA), *Withania somnifera* waterextract (WSW), *Withania somnifera* hydroalcoholicextract (WSHA).

**S2 Table: Centre coordinates of grids used for molecular docking**

|  | X-coordinate | Y-coordinate | Z-coordinate |
| --- | --- | --- | --- |
| Main Protease | 12.6655460652 | -1.53598778844 | 20.4843243045 |
| Spike Glycoprotein | 179.698237649 | 116.061225717 | 245.517112269 |
| RNA-dependent RNA polymerase | 112.252878786 | 117.486606725 | 133.028454188 |

**S3 Table: Structures of the phytoconstituents from AR, TC and WS**

| **Sr. No** | **Compound** | **Structure** |
| --- | --- | --- |
| 1 | Asparagamine A | 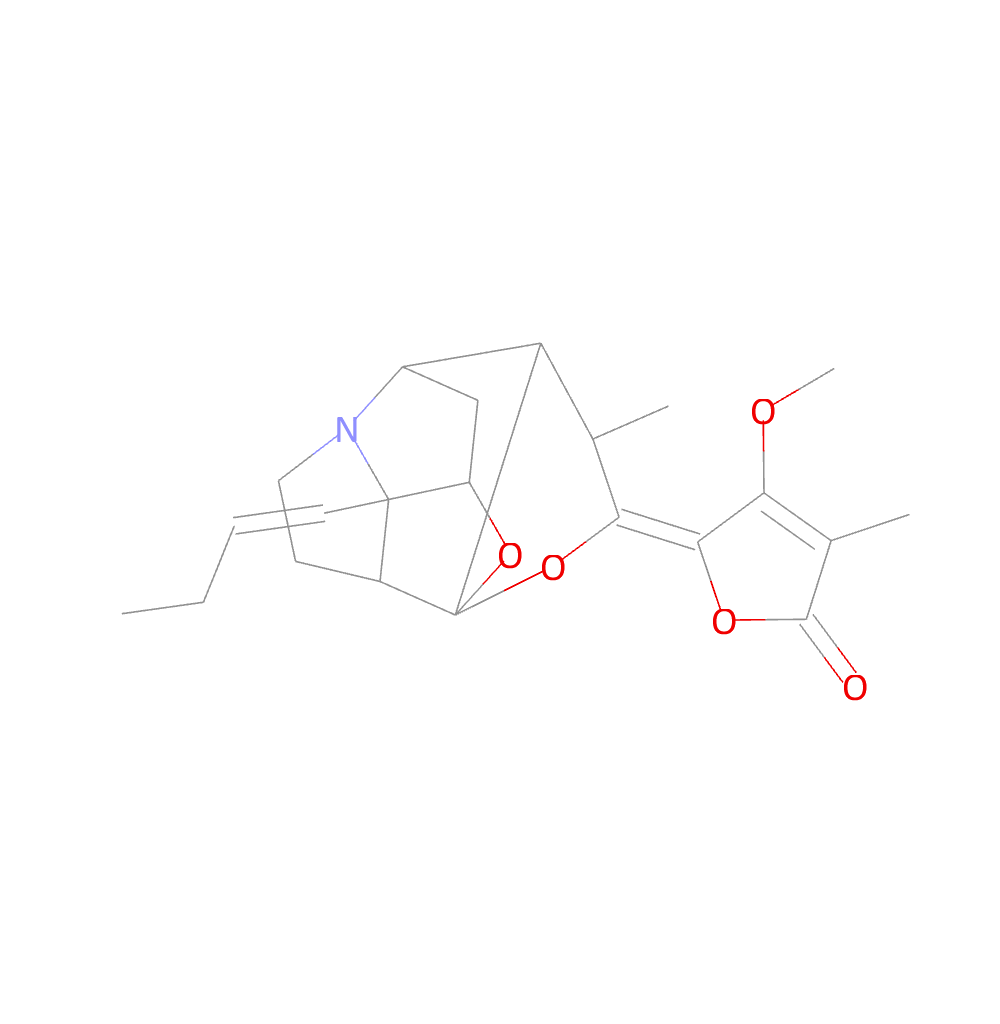 |
| 2 | Asparanin A | 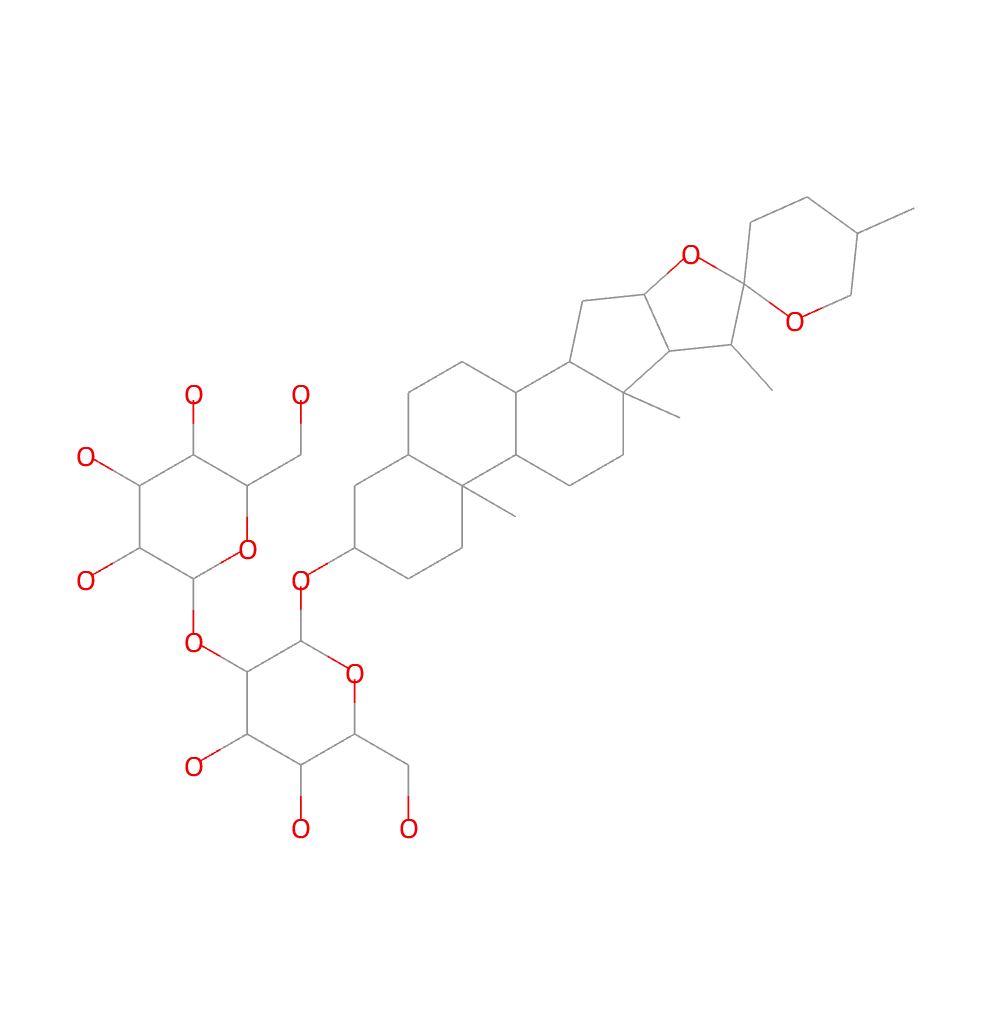 |
| 3 | Isoagatharesinol | 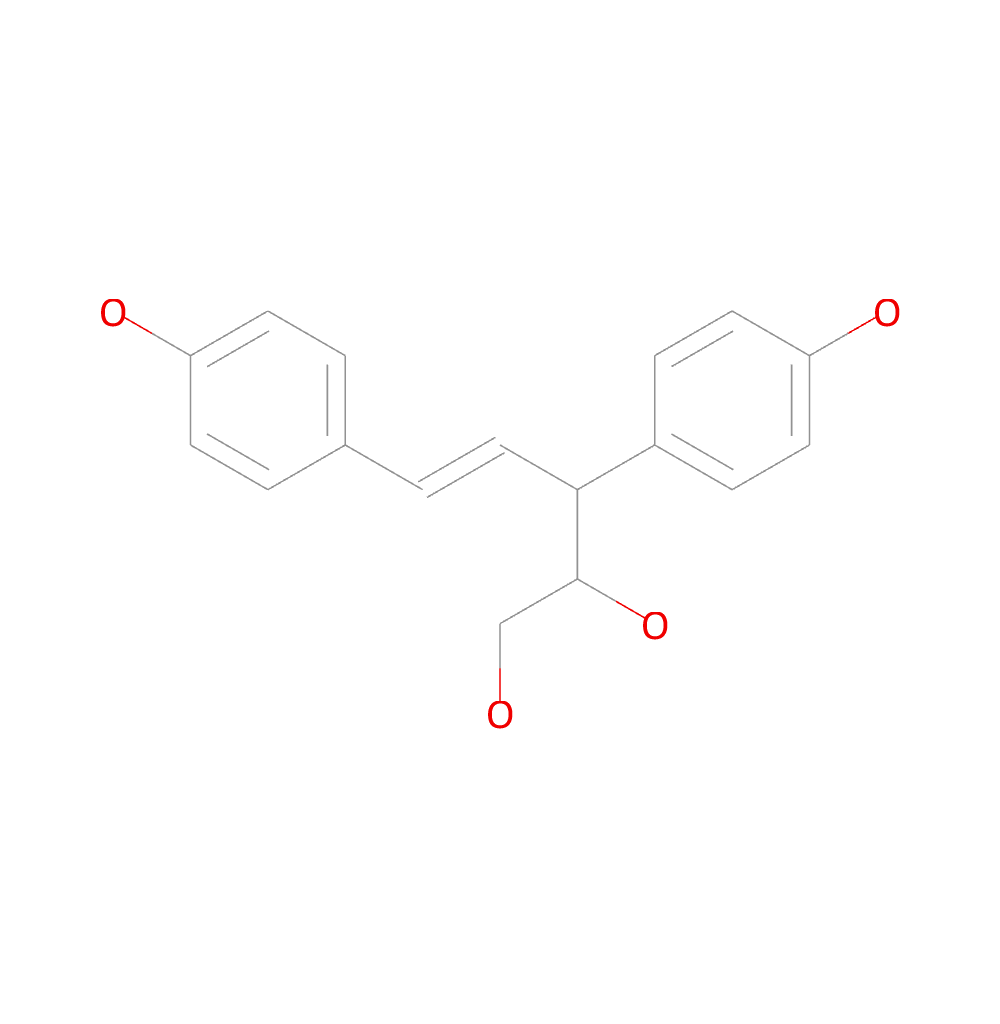 |
| 4 | Muzanzagenin | 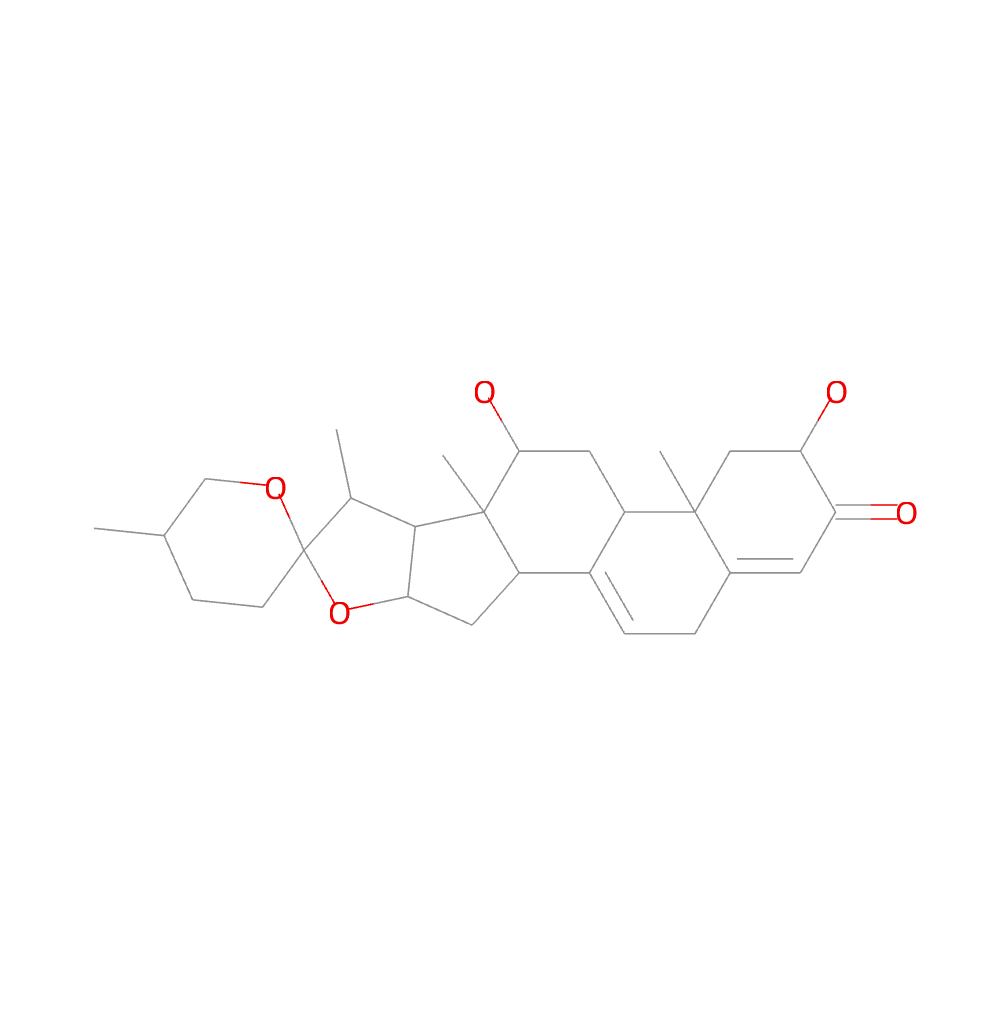 |
| 5 | Rutin | 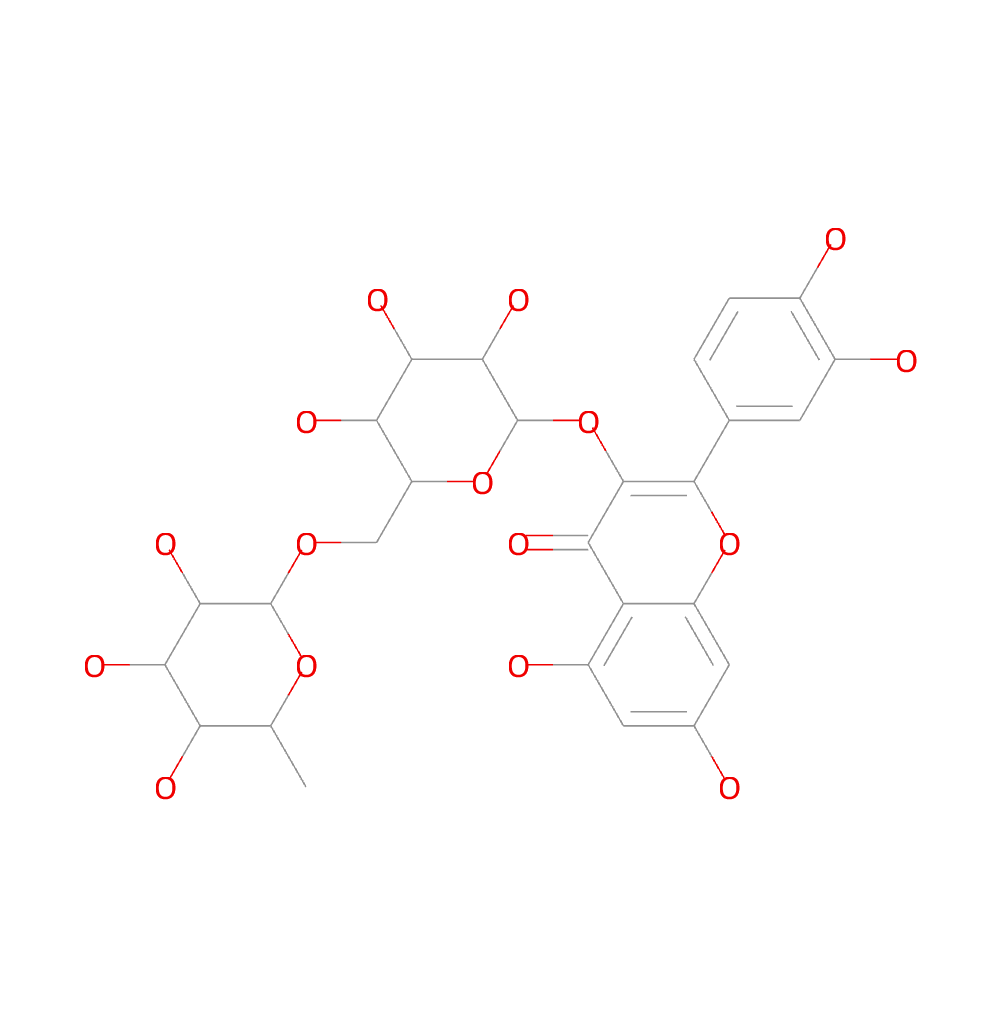 |
| 6 | ShatavarinI | 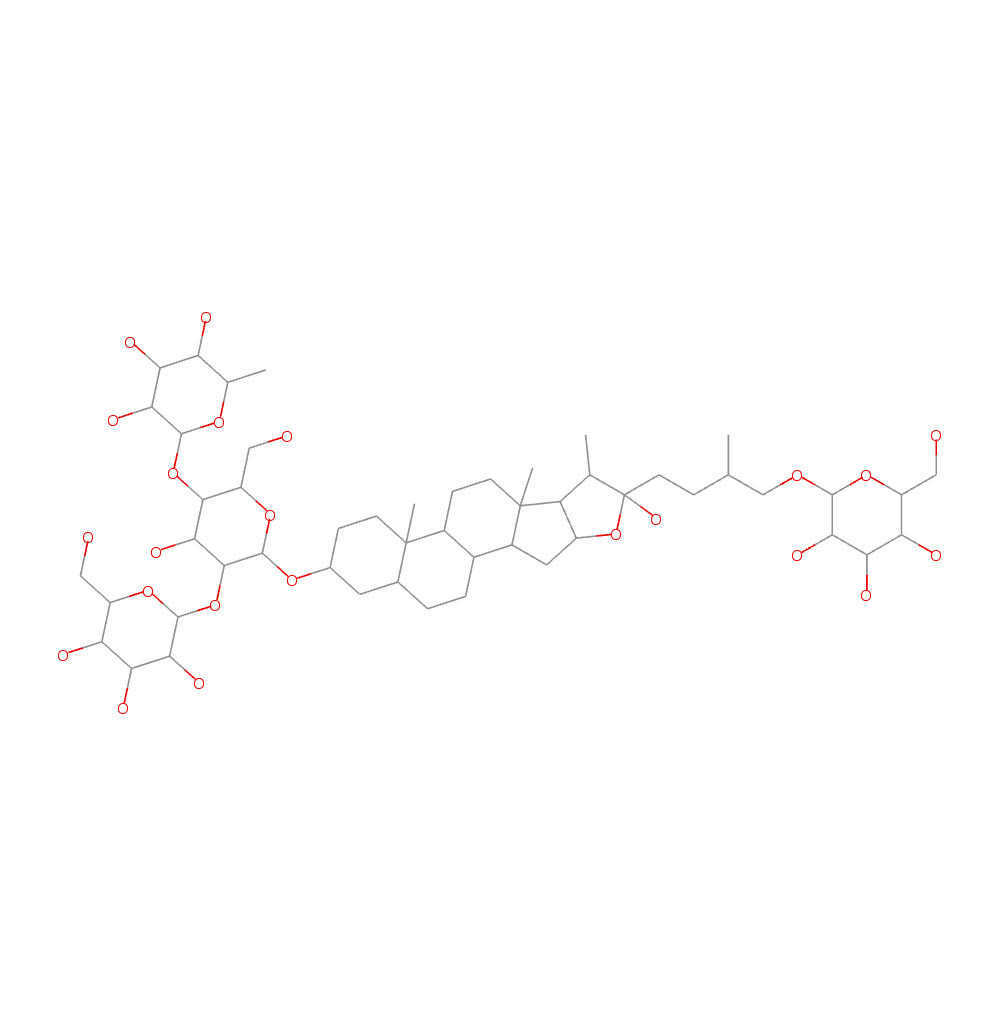 |
| 7 | ShatavarinIV | 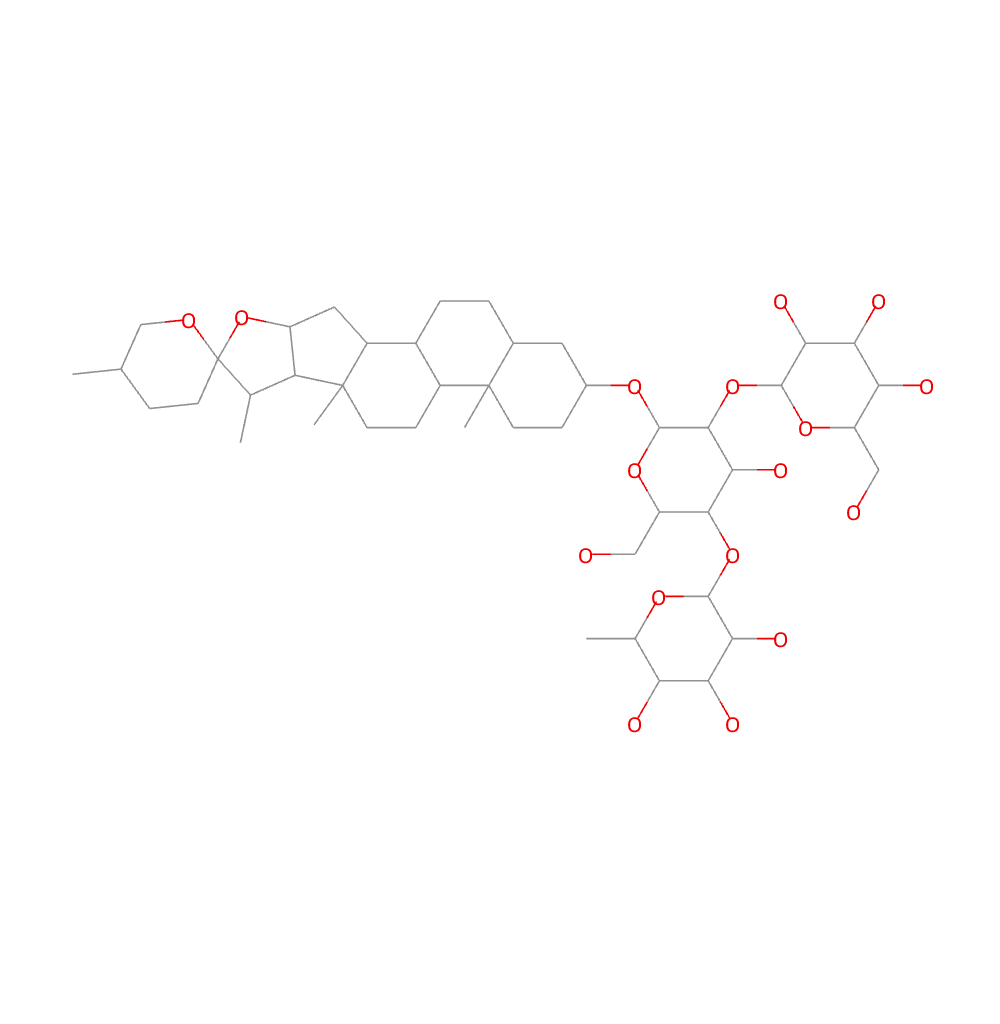 |
| 8 | ShatavarinIX | 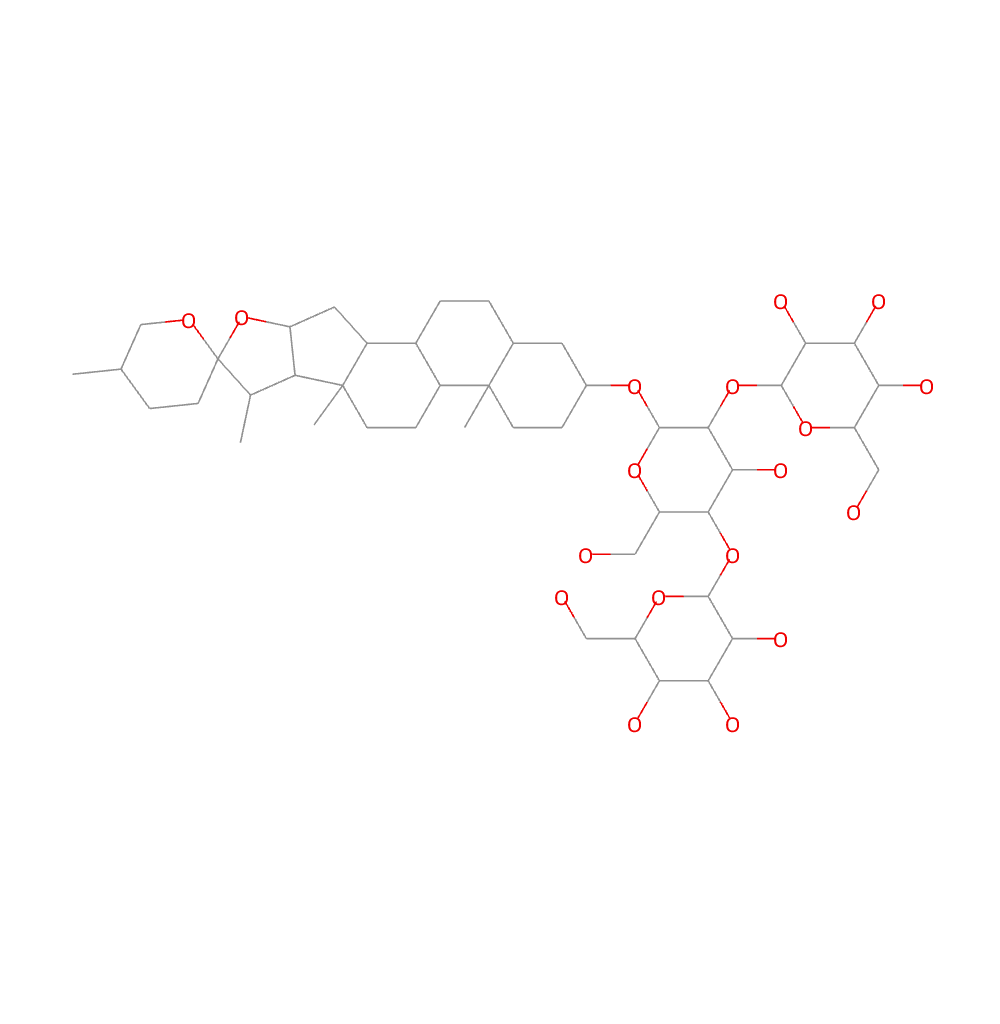 |
| 9 | ShatavarinVI | 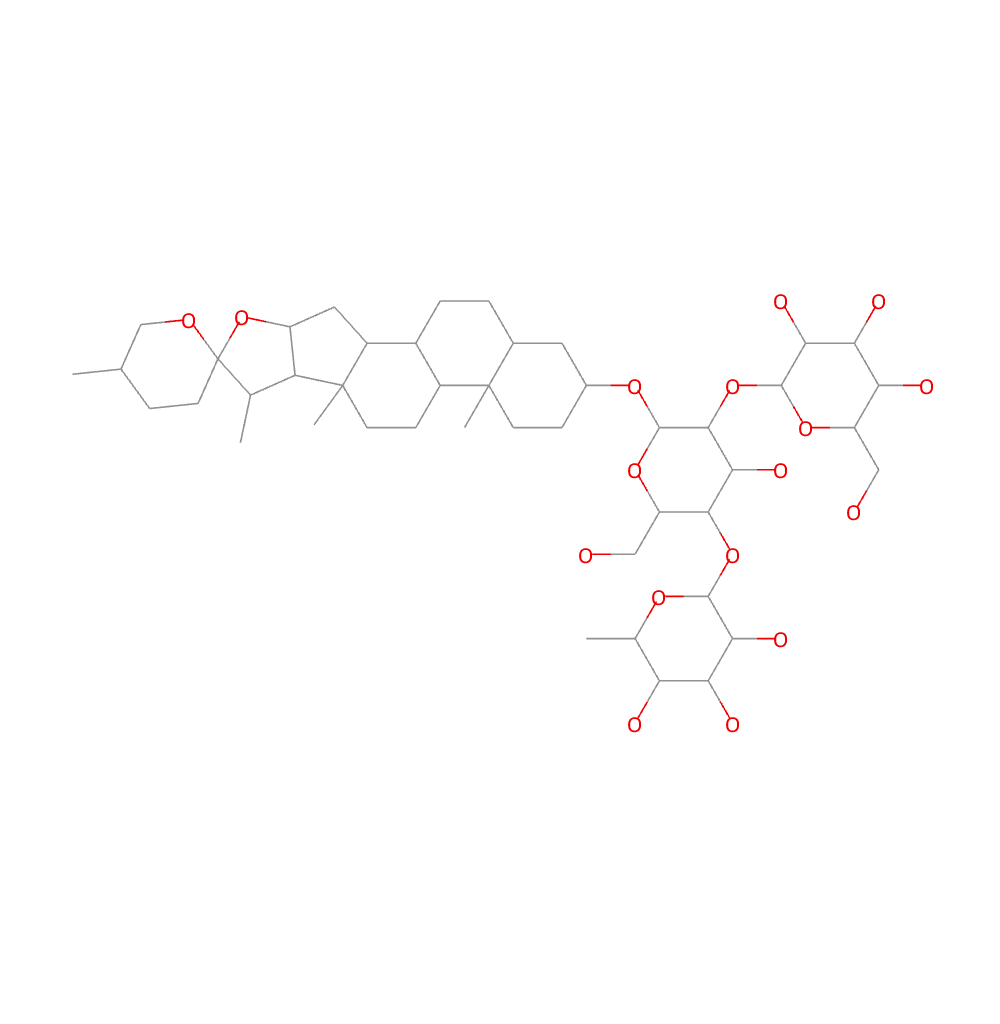 |
| 10 | ShatavarinVII | 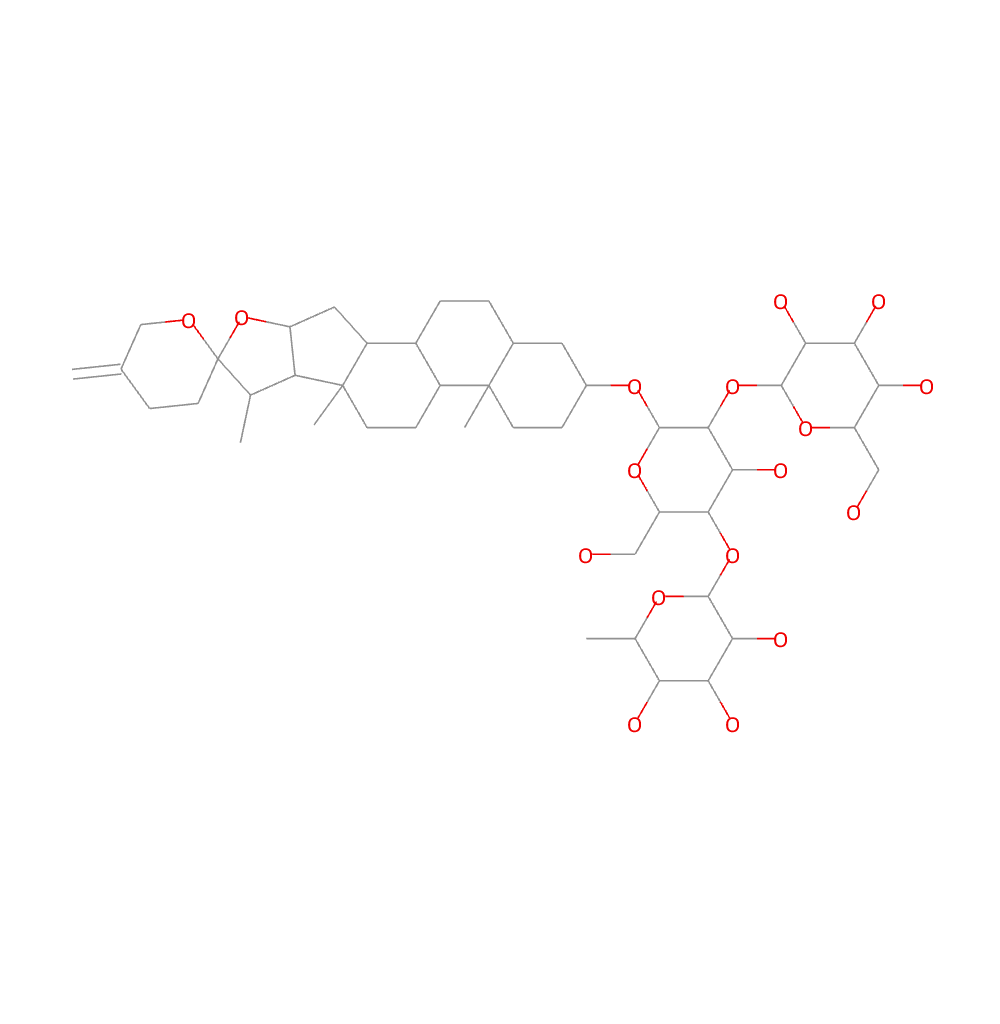 |
| 11 | ShatavarinX | 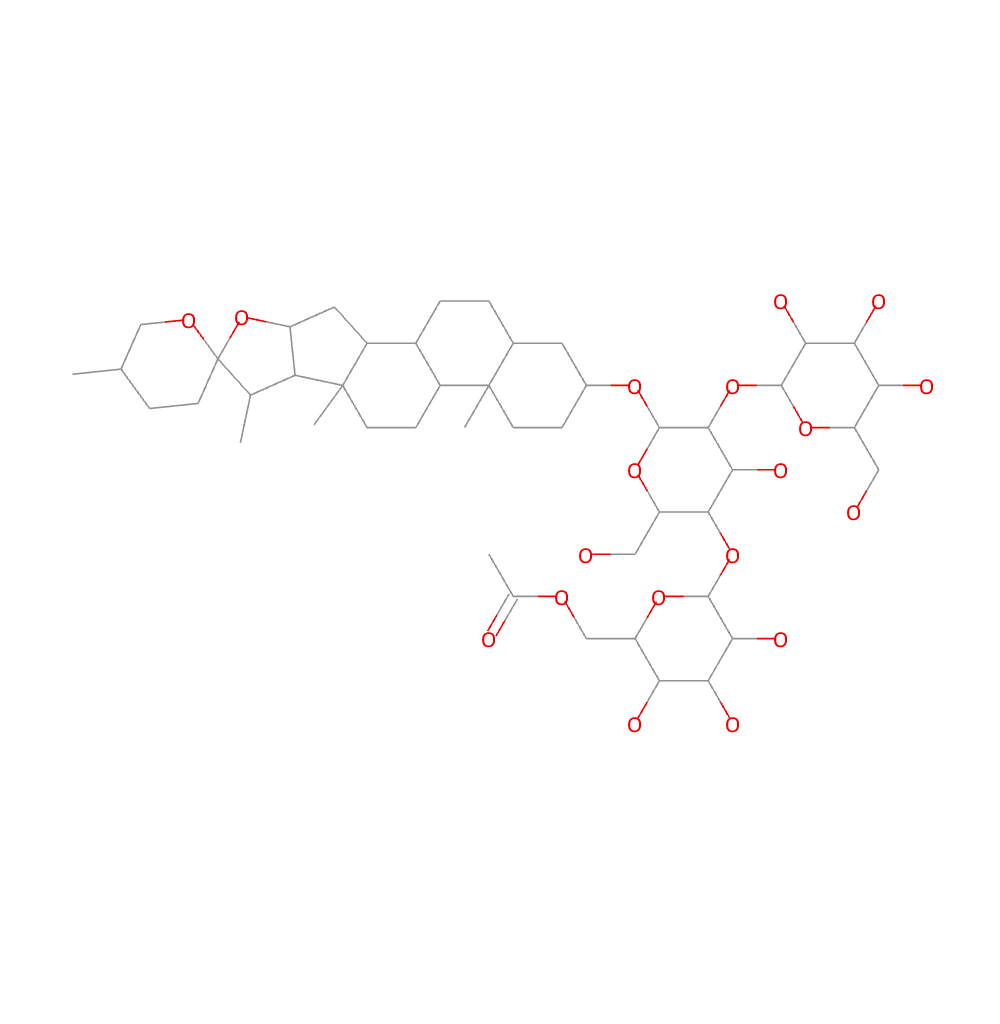 |
| 12 | 20-Hydroxy Ecdysone | 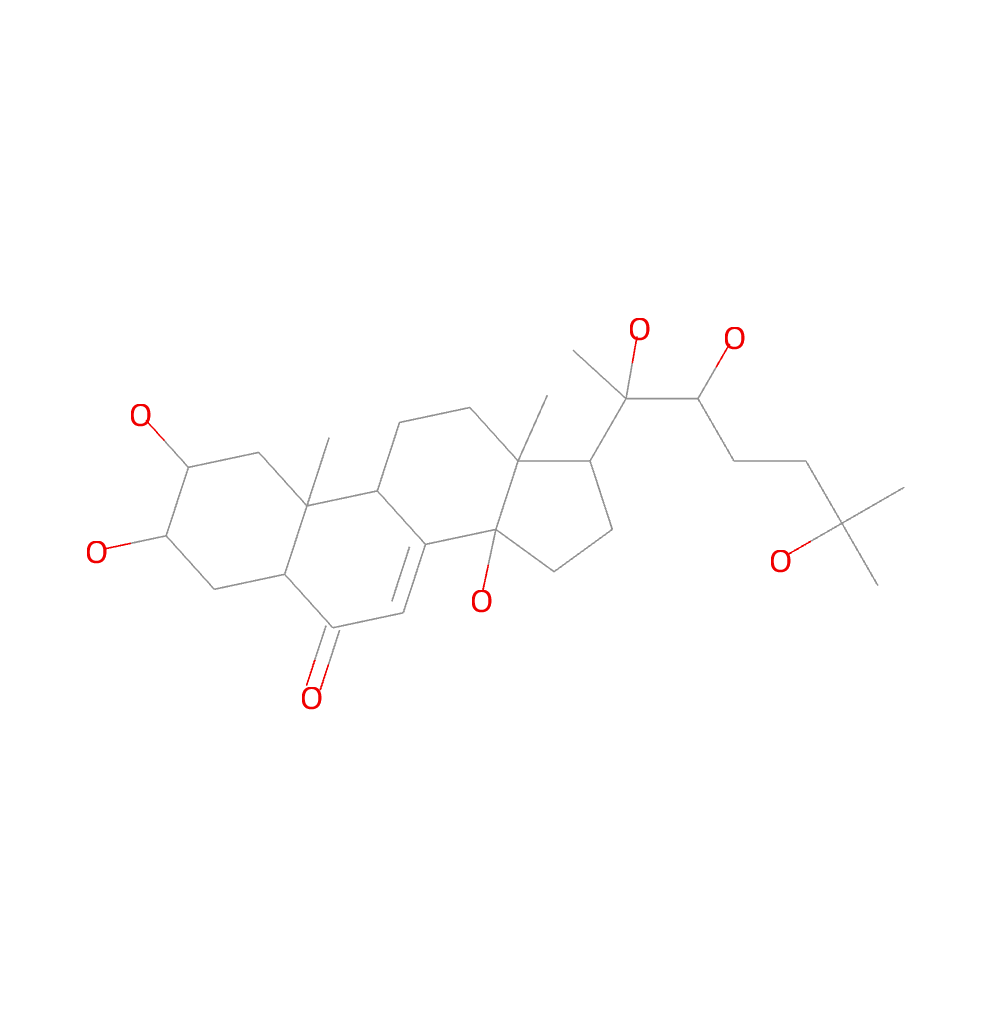 |
| 13 | Berberine | 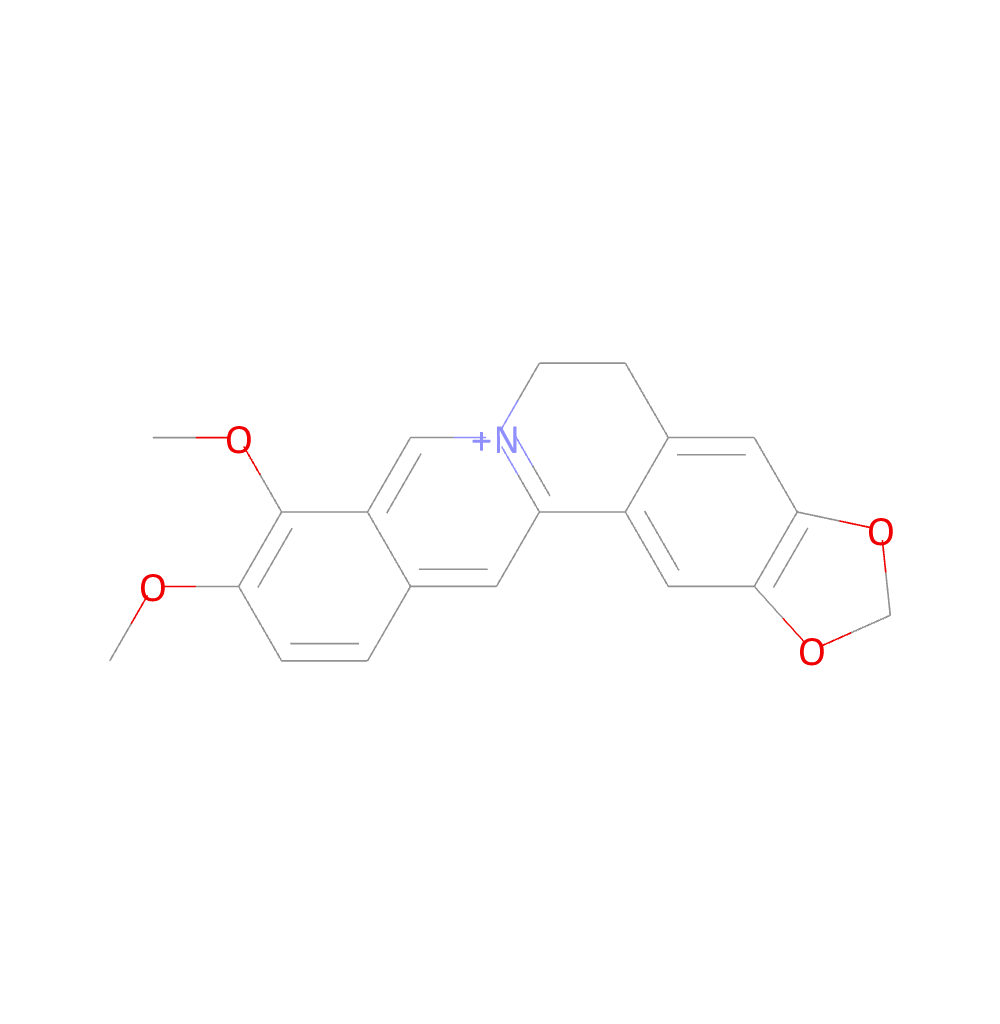 |
| 14 | Columbamine | 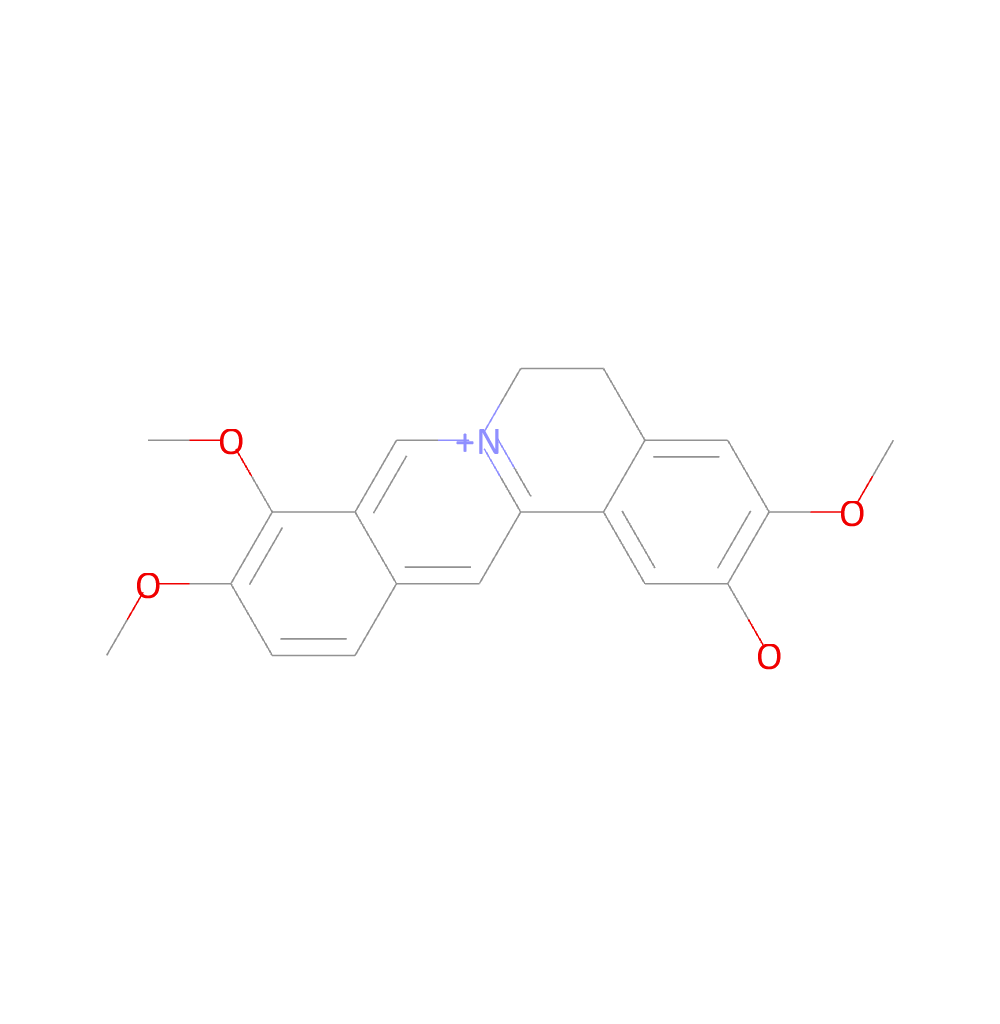 |
| 15 | Columbin | 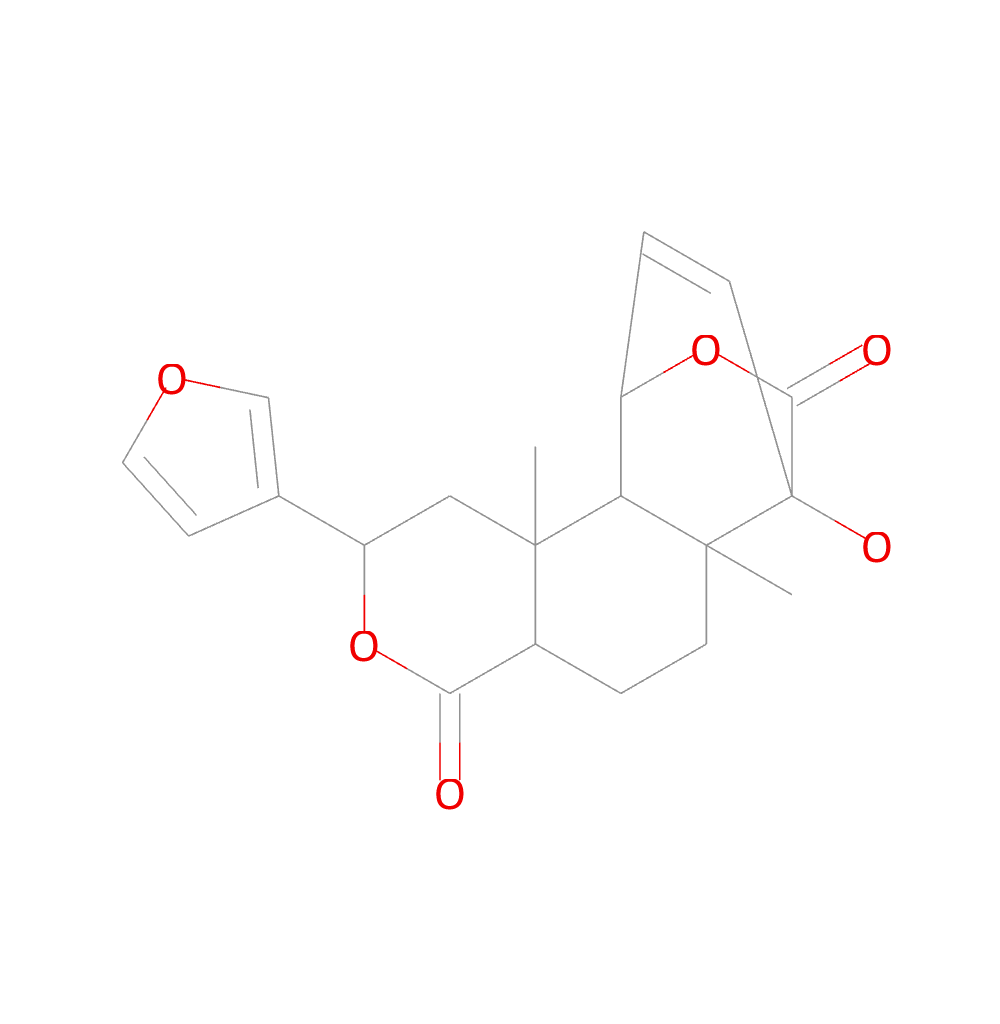 |
| 16 | Magnoflorine | 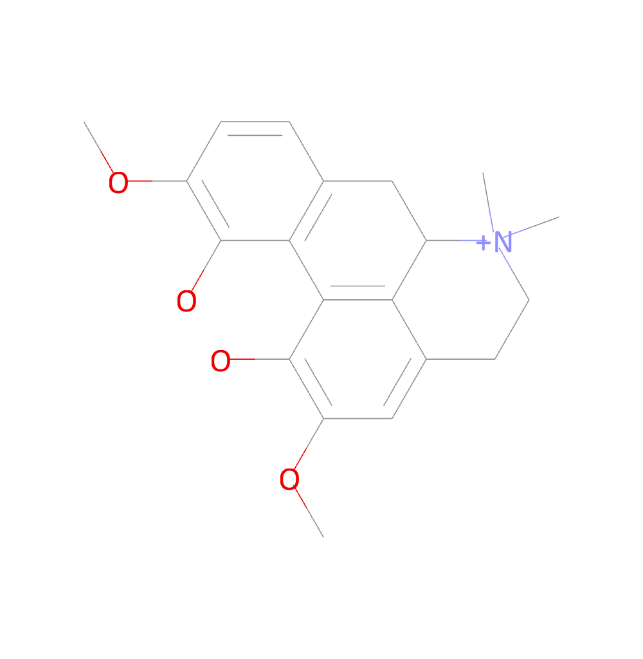 |
| 17 | Menisperine | 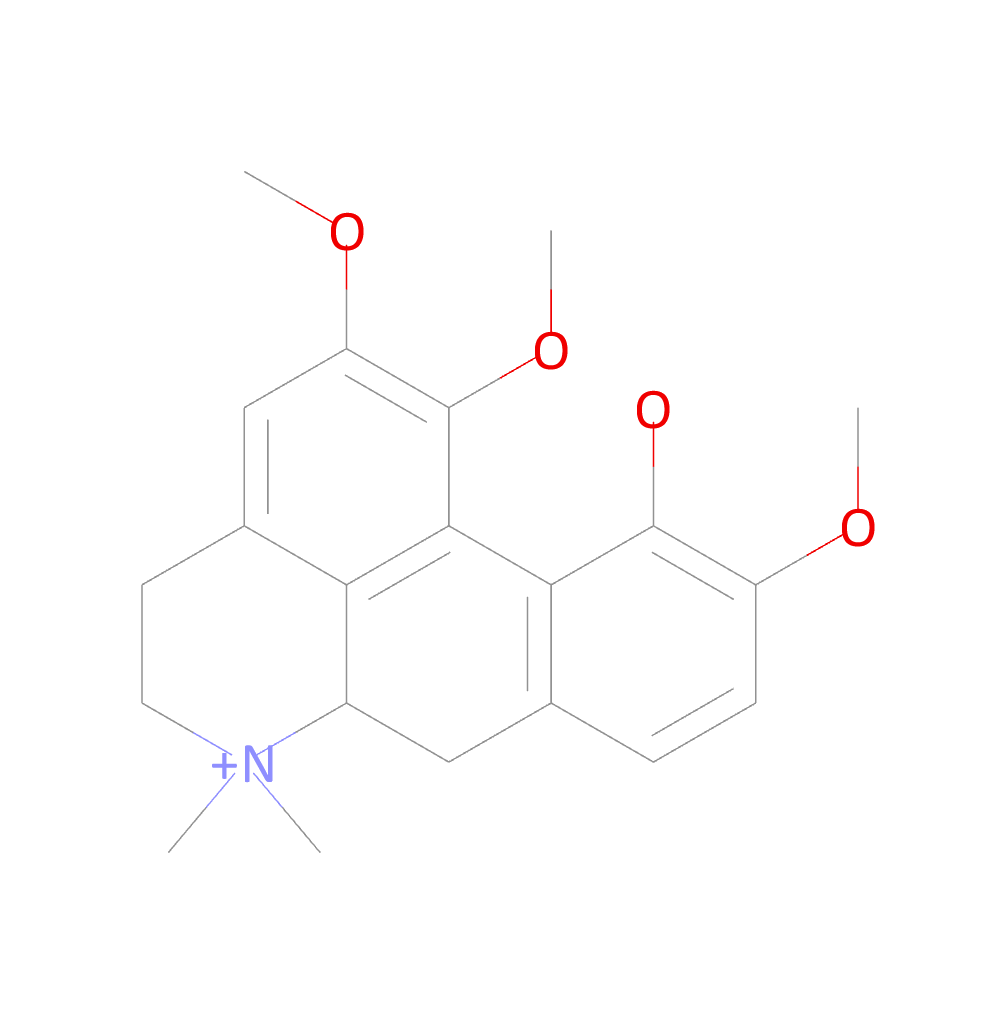 |
| 18 | Syringin | 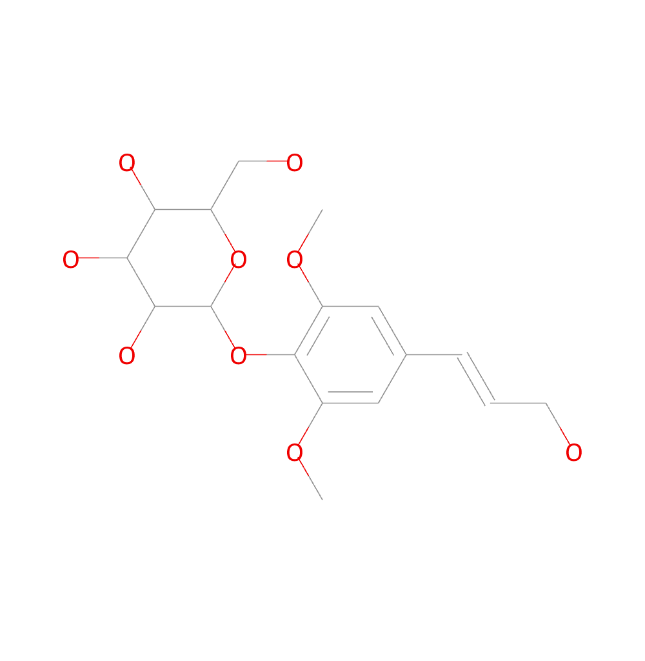 |
| 19 | Tinocordiside | 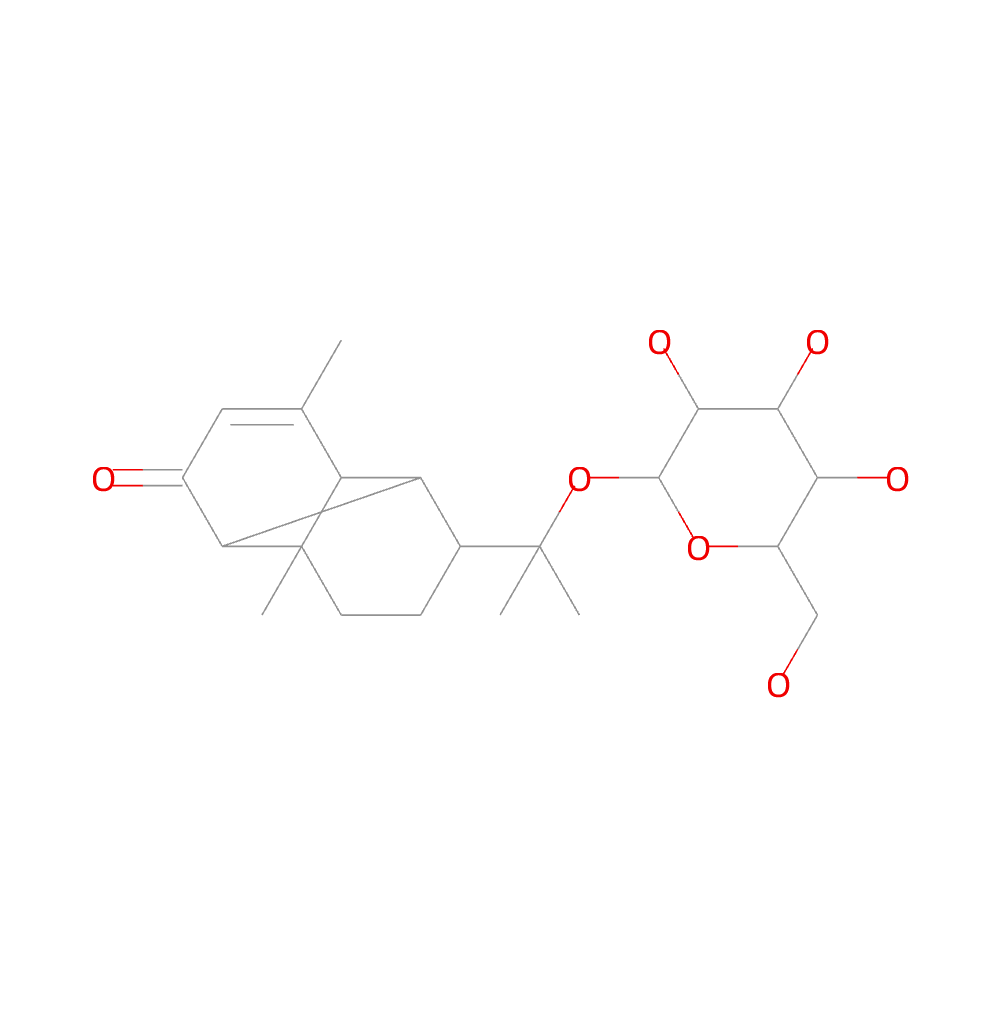 |
| 20 | Tinosporaside | 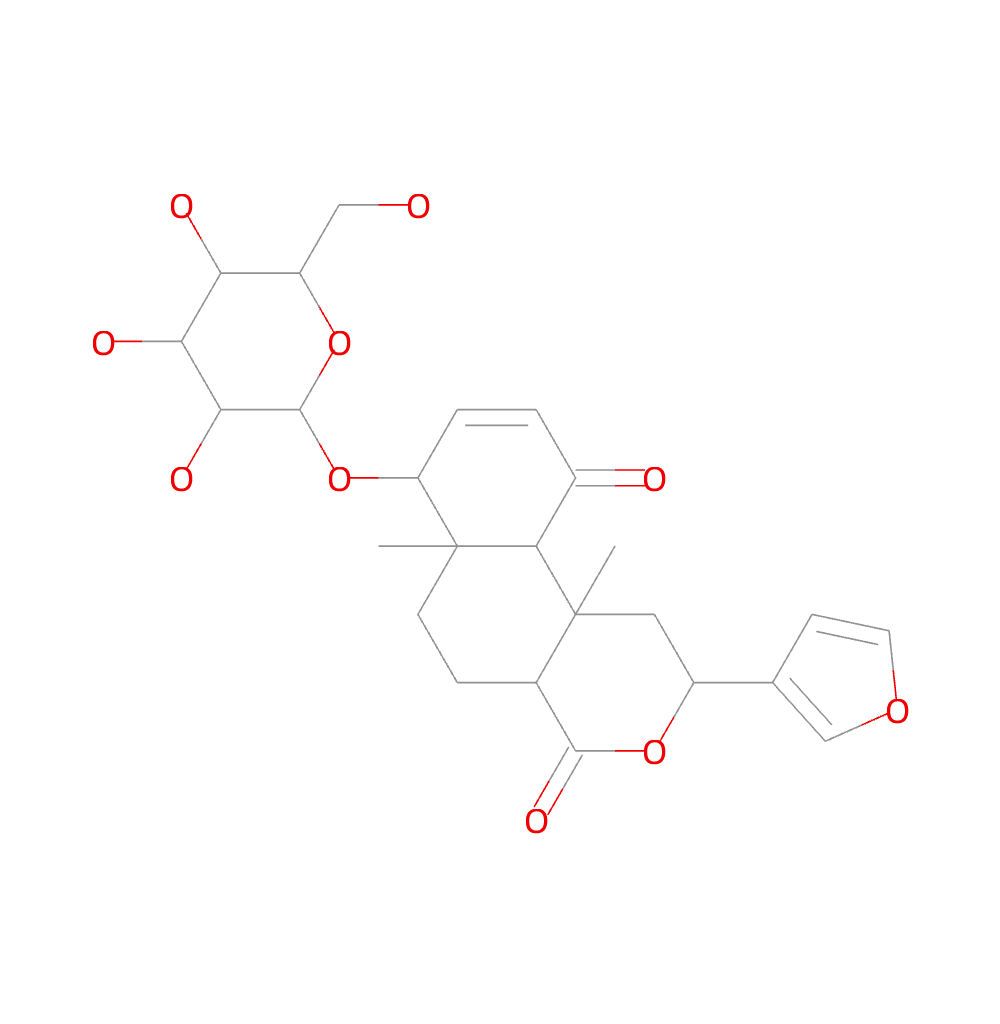 |
| 21 | Tinosporide | 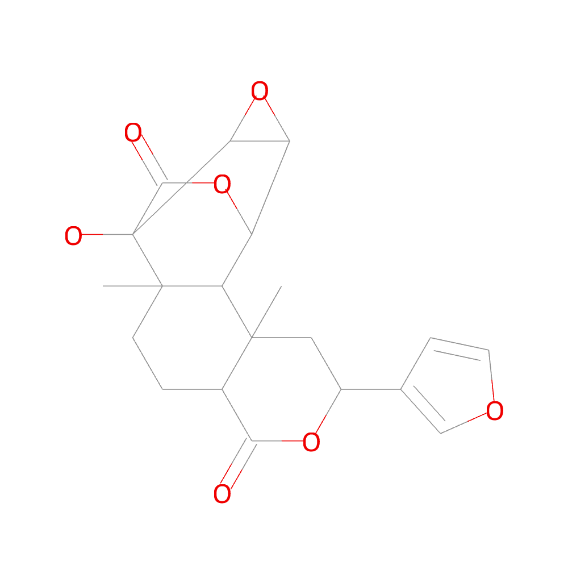 |
| 22 | 12-Deoxywithastramonolide | 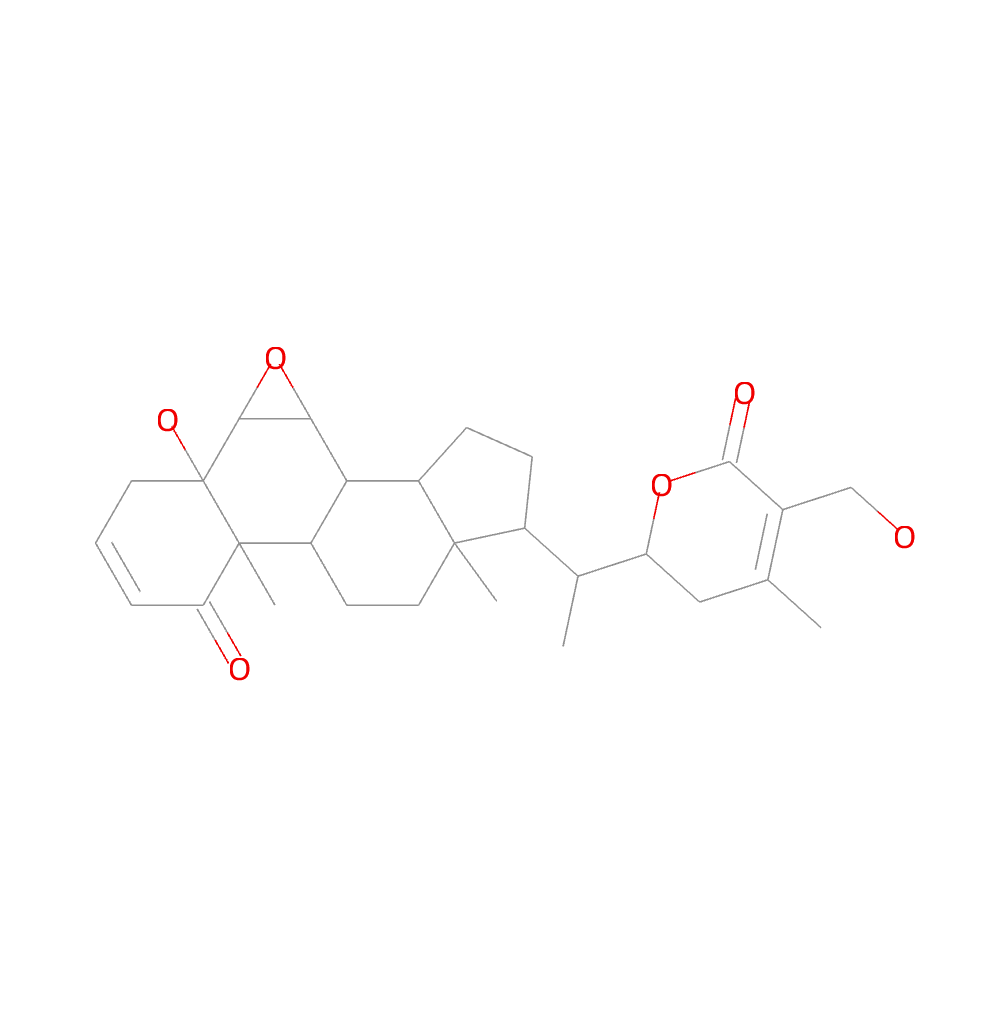 |
| 23 | 27-Hydroxywithanone | 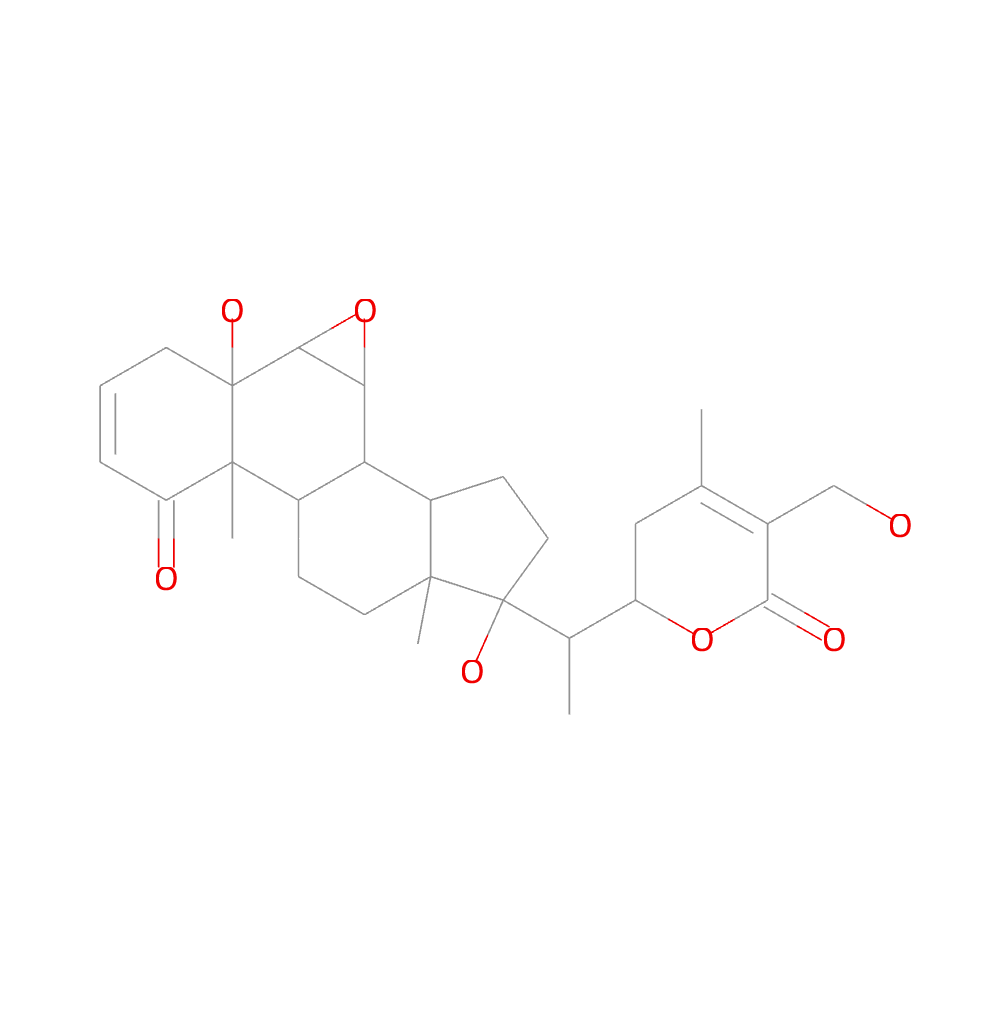 |
| 24 | Ashwagandhanolide | 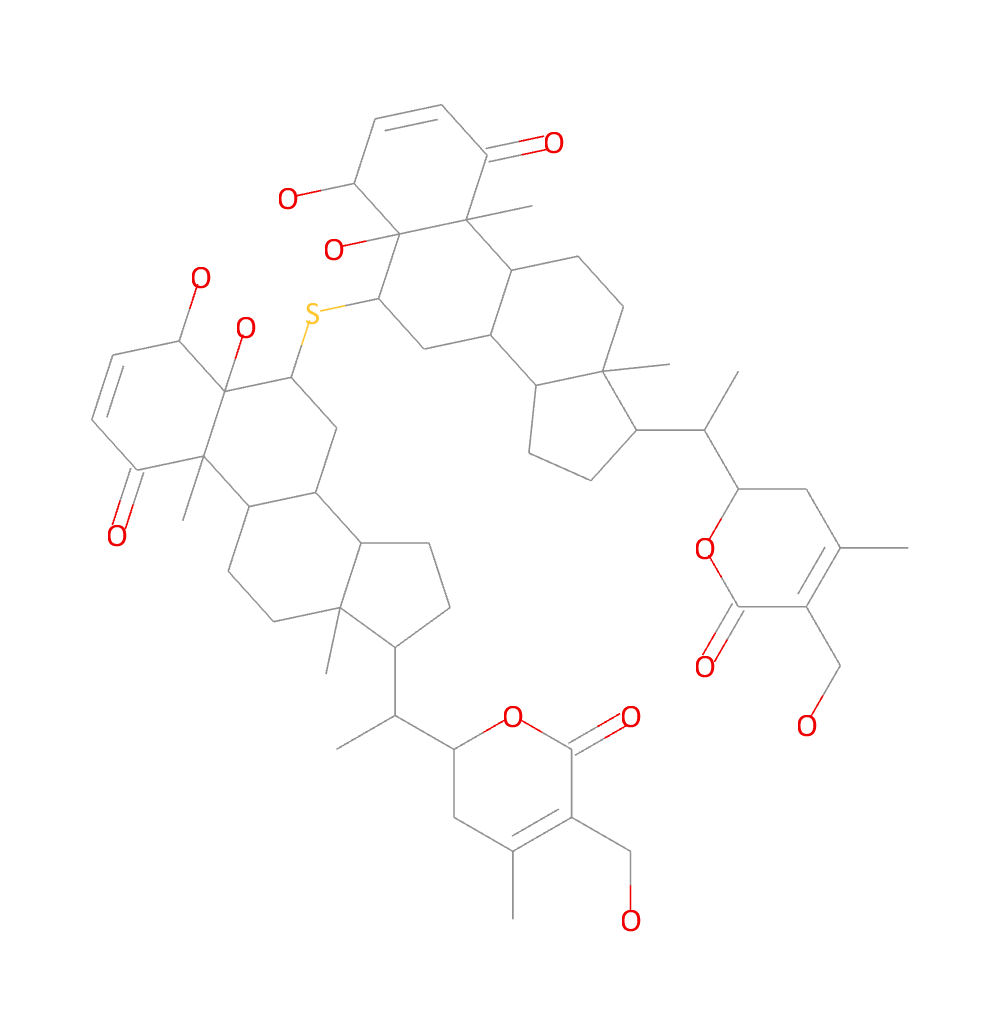 |
| 25 | Withacoagin | 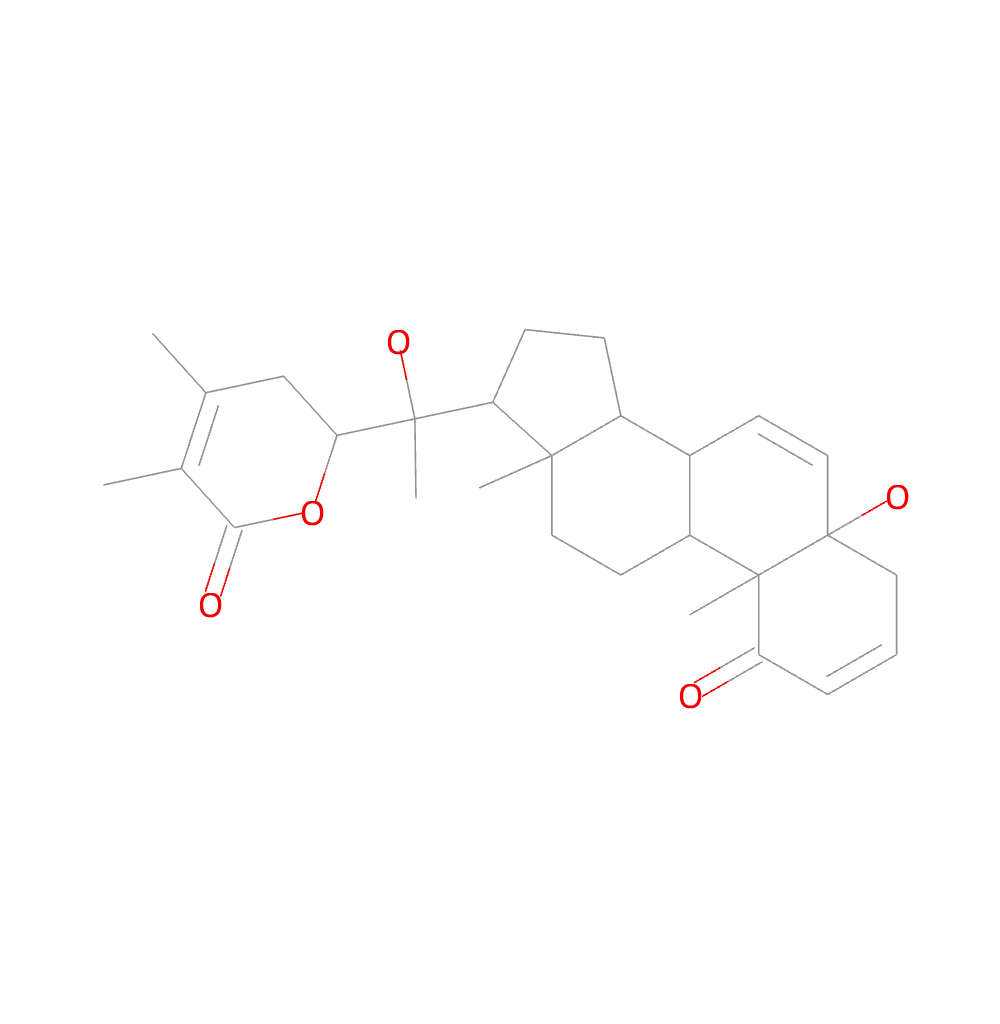 |
| 26 | Withaferin | 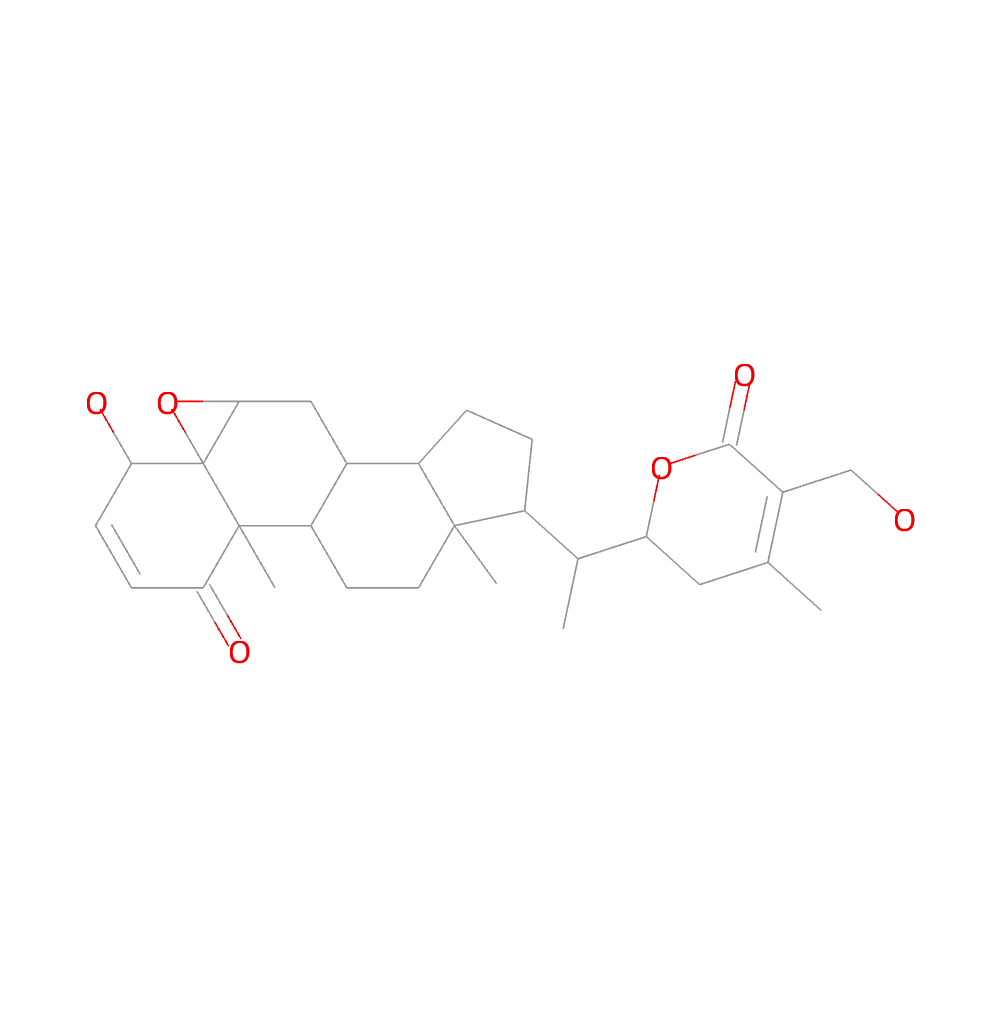 |
| 27 | WithanolideA | 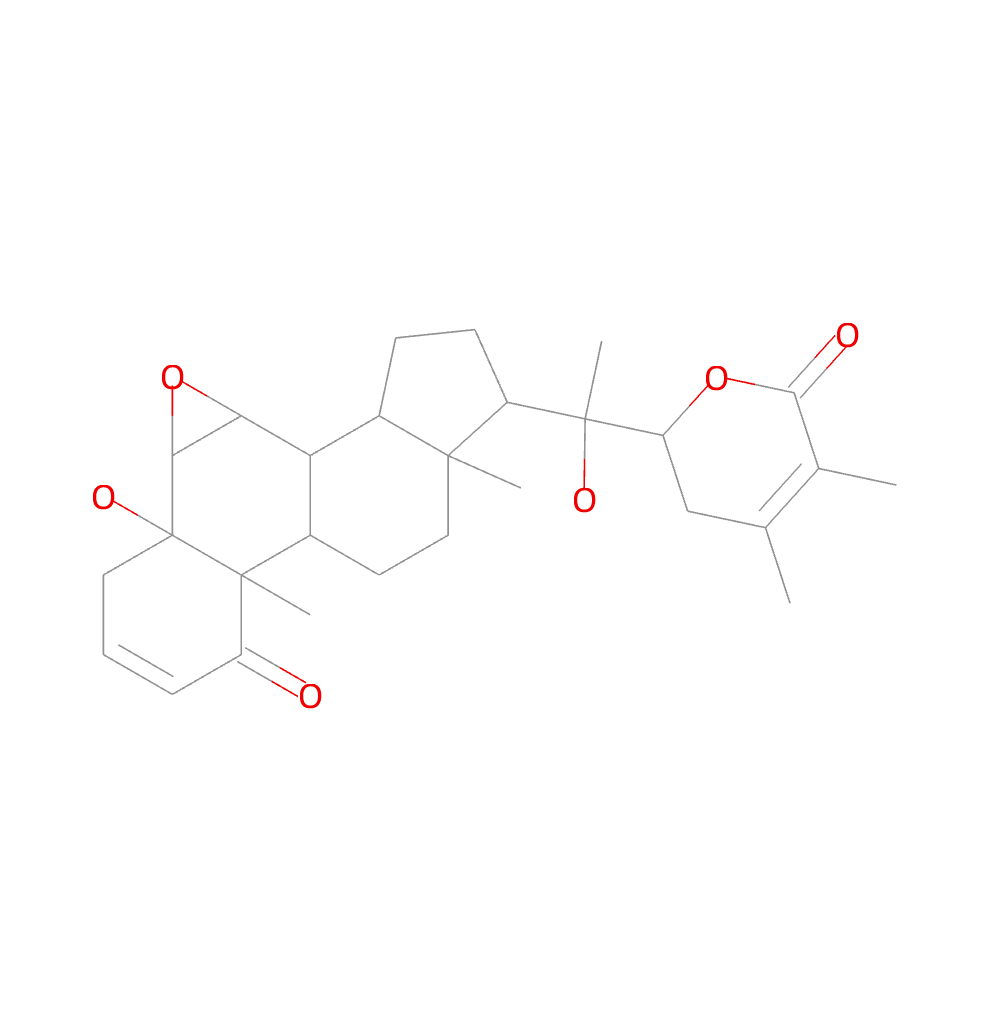 |
| 28 | WithanolideB | 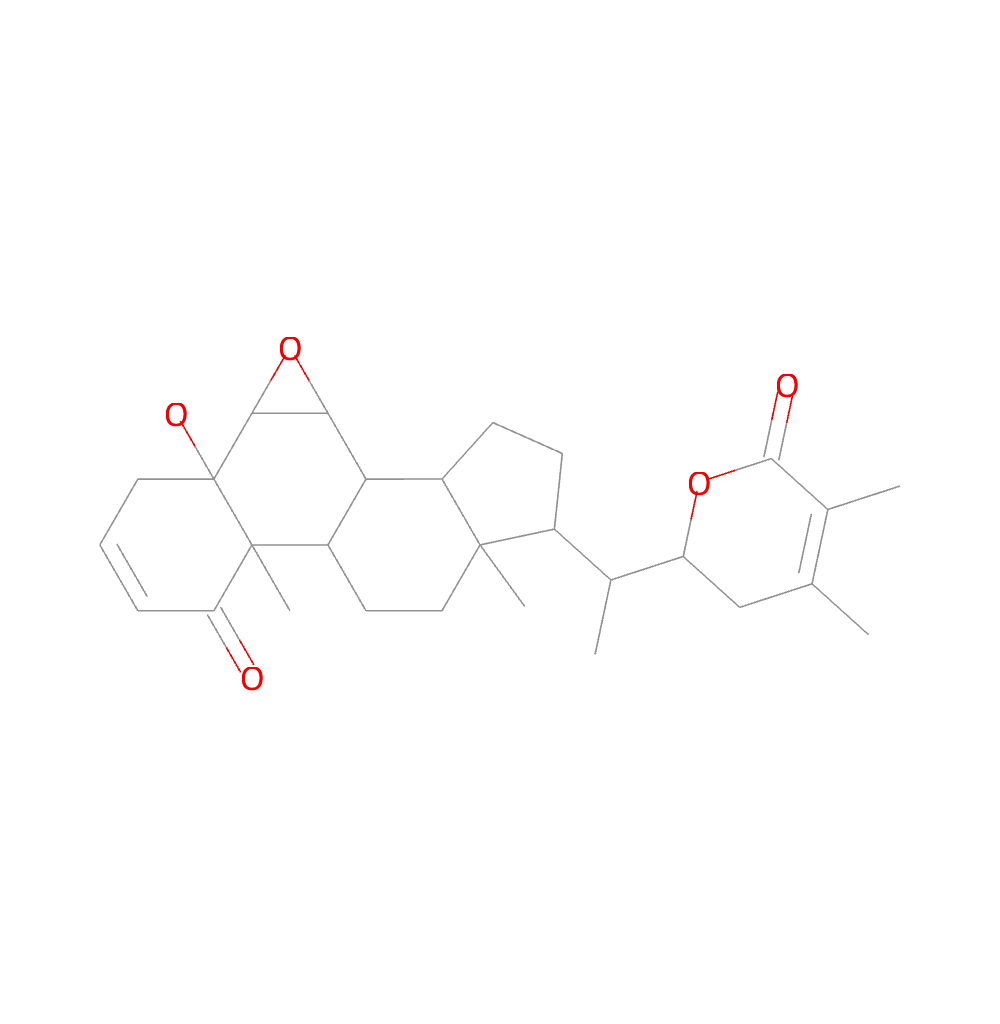 |
| 29 | Withanone | 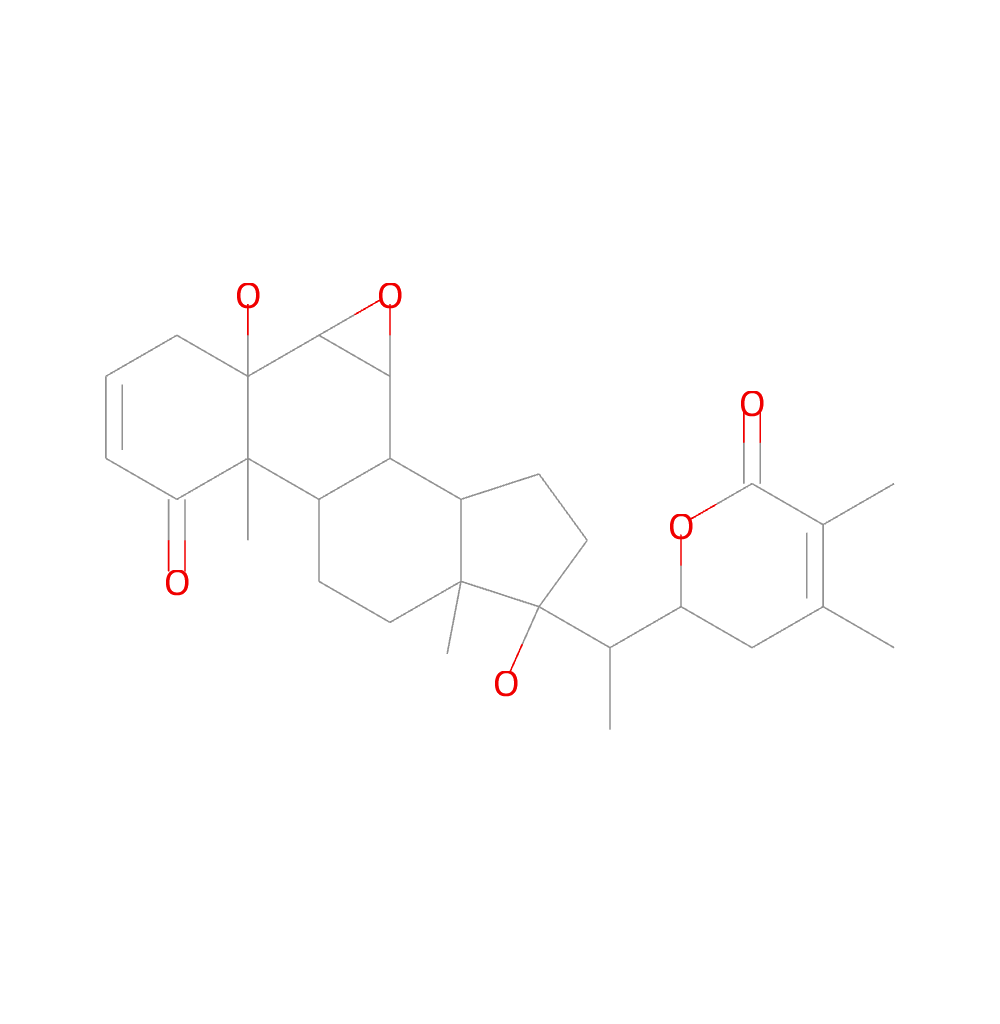 |
| 30 | WithanosideIV | 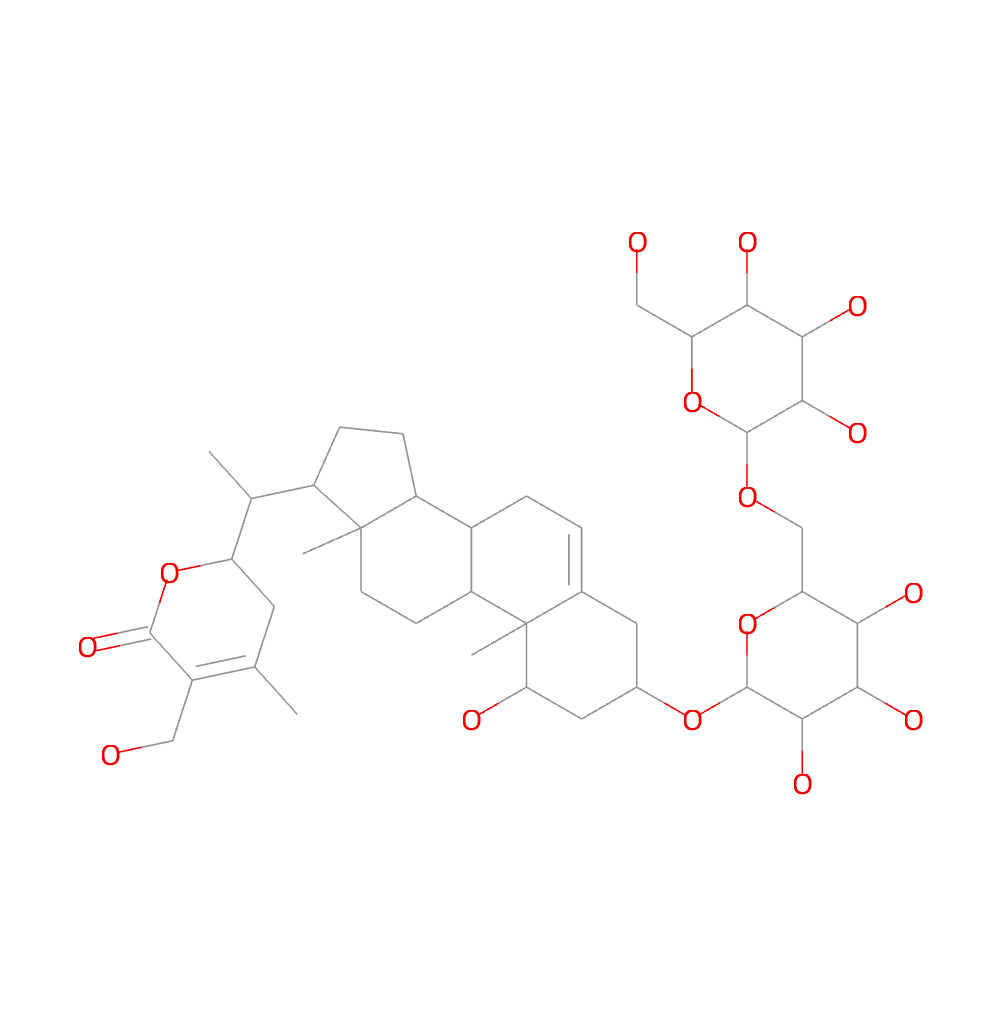 |
| 31 | WithanosideV | 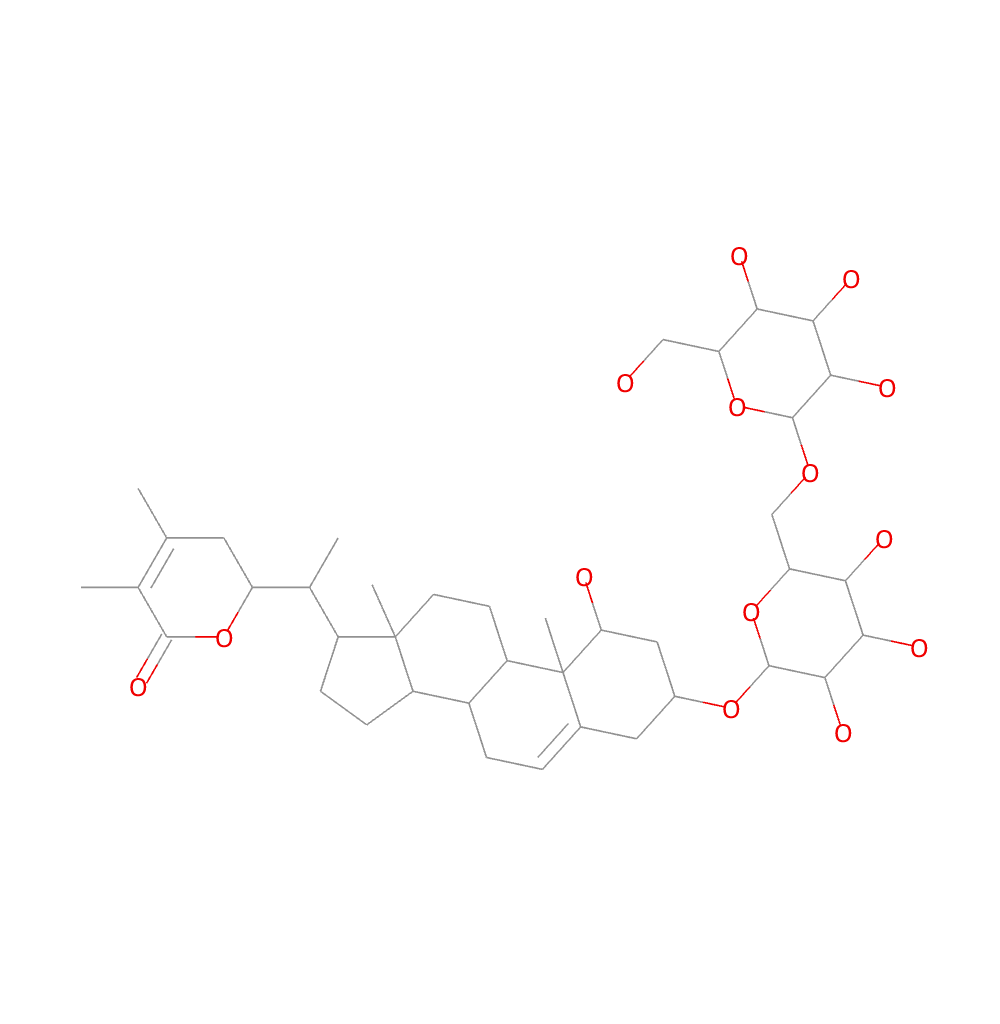 |

**S1 Fig. Structural characterization of top docked complexes after simulation**

**
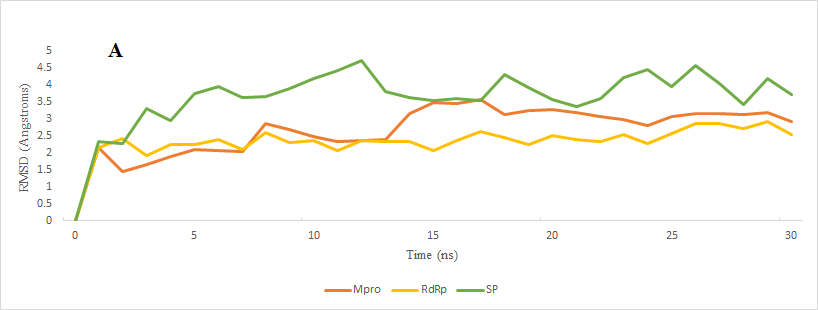

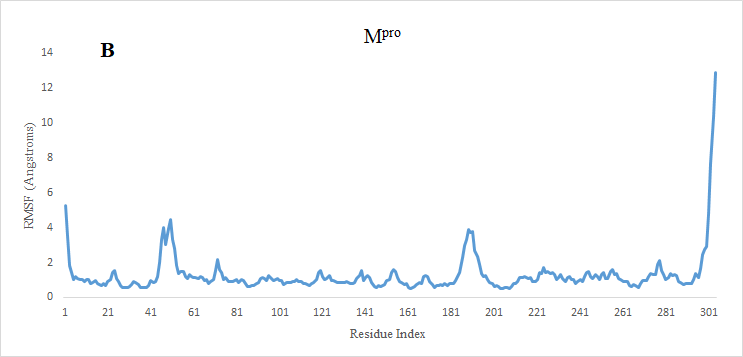
**

**
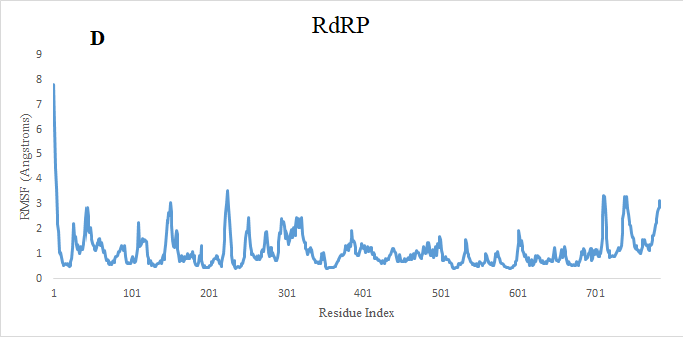

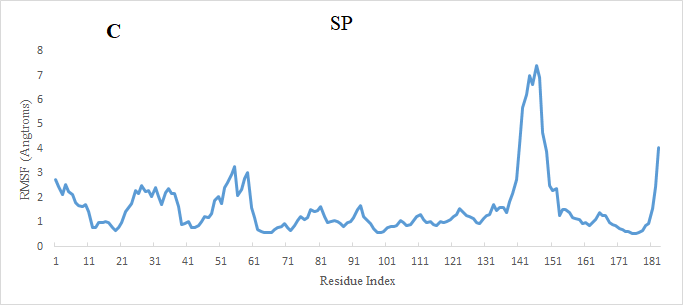
**

1. **RMSD plot of docked complexes. RMSF plots of B) Main Protease C) Spike Protein and D) RNA dependent RNA polymerase**


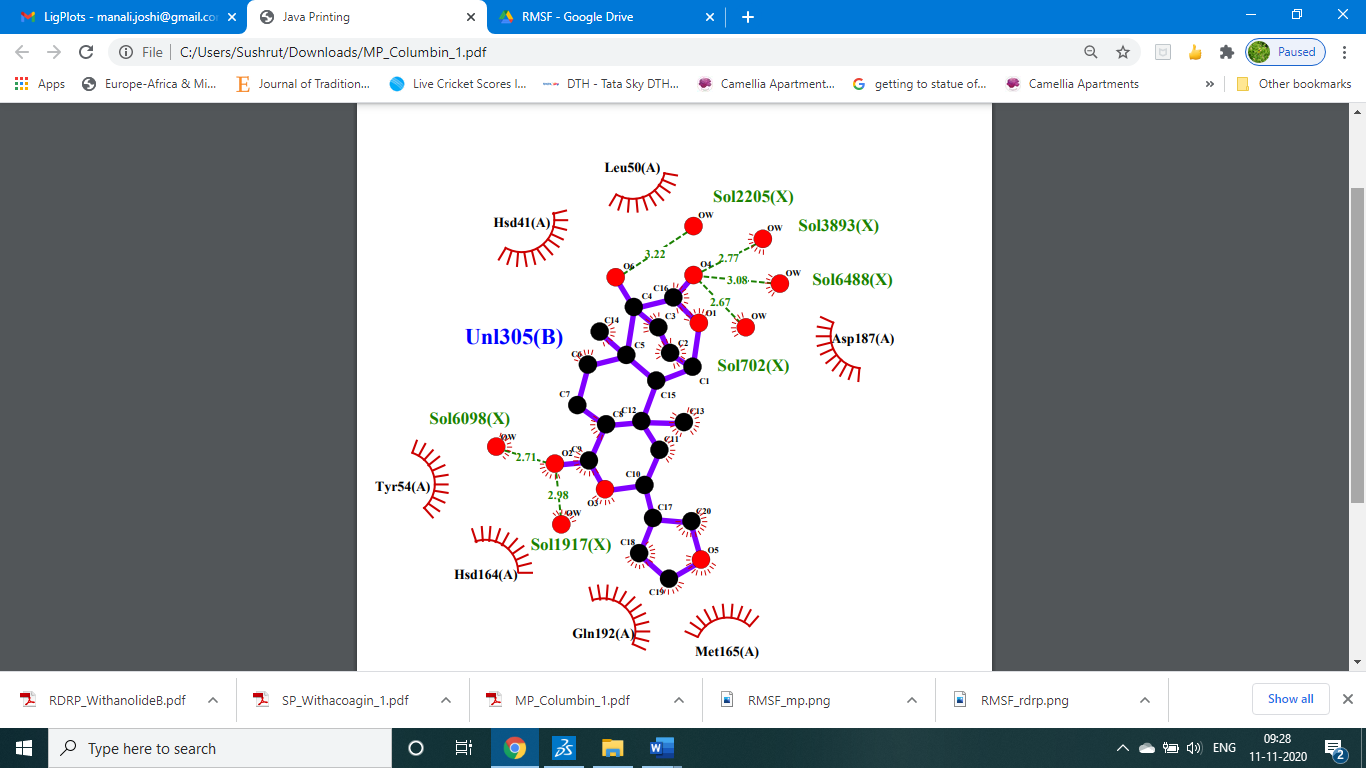

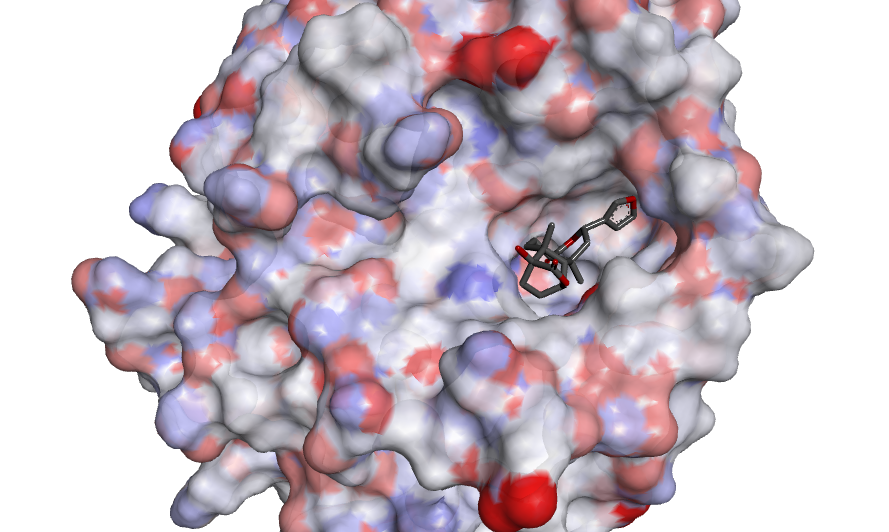
S2 Fig. Interaction of Columbin with M^pro^. In the left panel the protein is shown in a surface representation coloured by atom while the ligand is shown in stick representation. The right panel is a 2D interaction plot of the receptor with the inhibitor.


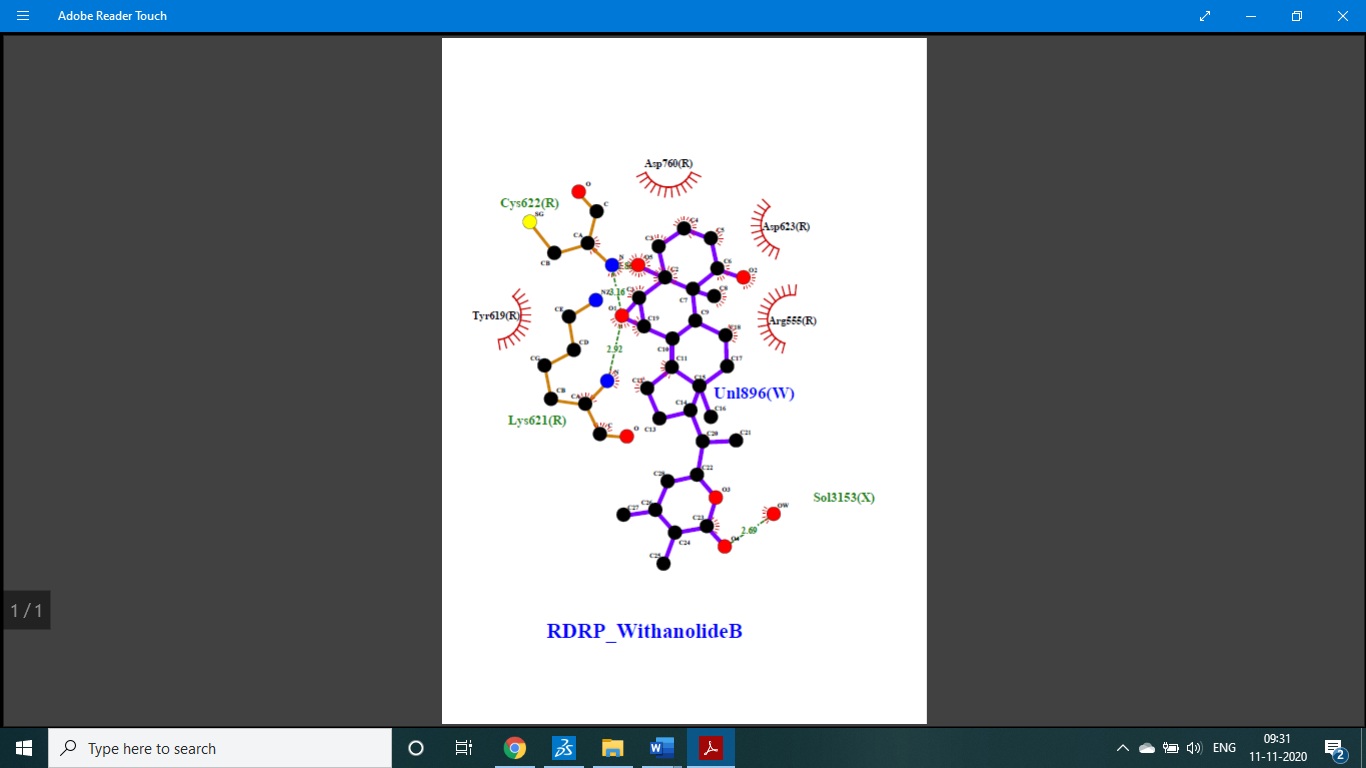
**S3 Fig. Interaction of Withanolide-B with RdRp.** In the left panel the protein is shown in a surface representation coloured by atom while the ligand is shown in stick representation. The right panel is a 2D interaction plot of the receptor with the inhibitor.

**
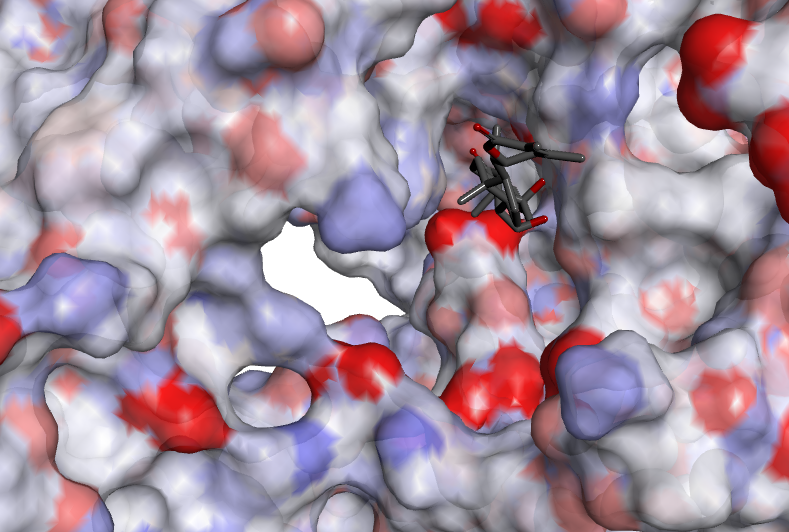
**


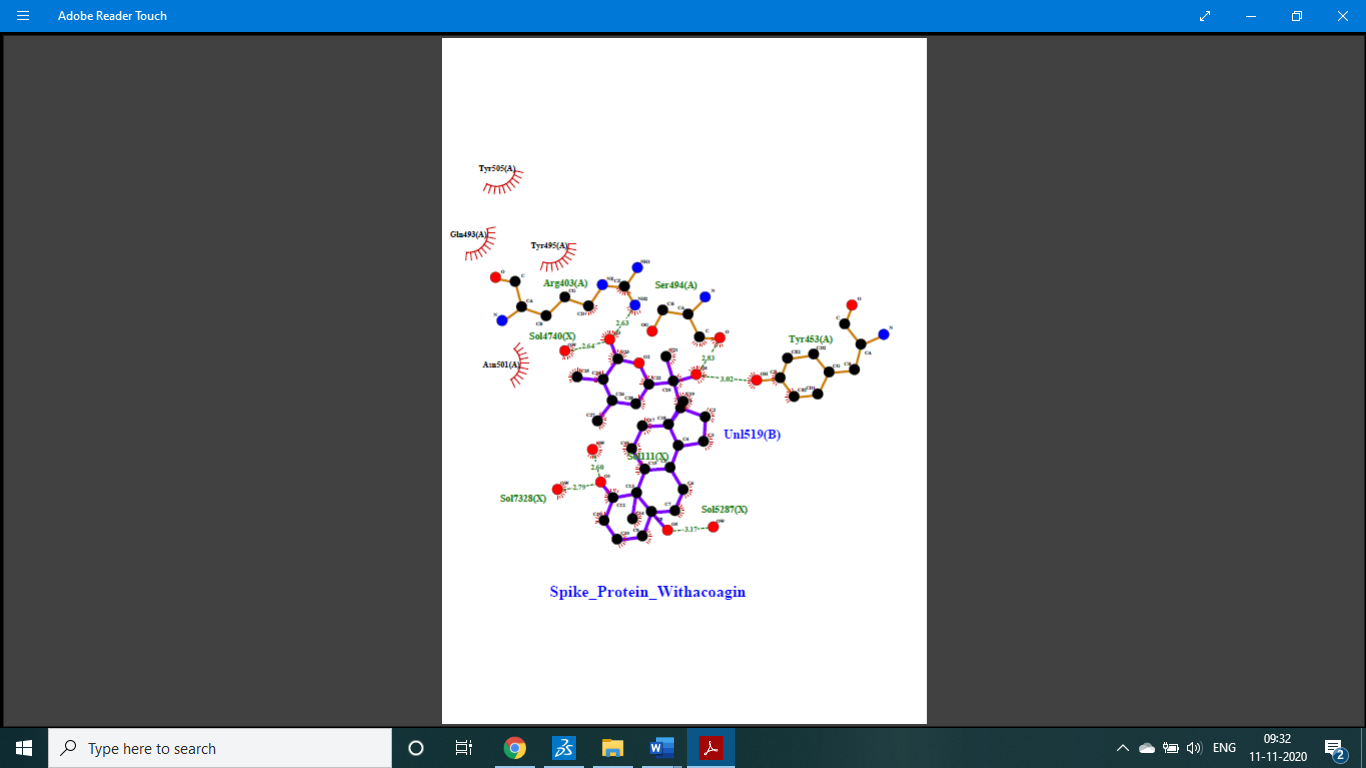
**S4 Fig. Interaction Withanolide-B with Spike protein.** In the left panel the protein is shown in a surface representation coloured by atom while the ligand is shown in stick representation. The right panel is a 2D interaction plot of the receptor with the inhibitor.


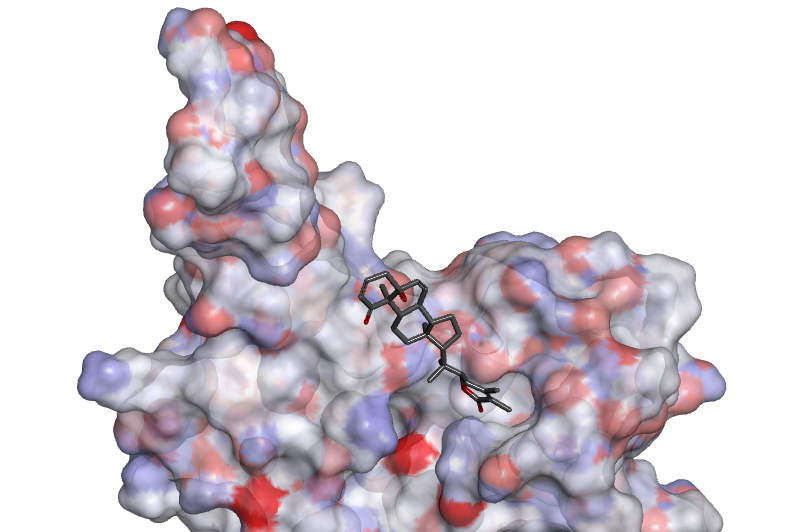


**S5 Fig. Bioavailability RADAR graphs for phytochemicals of AR, TC and WS.**

Bioavailability RADAR graph represents five important properties lipophilicity, size, polarity, solubility, saturation and flexibility for predicting whether test molecule is orally bioavailable or not. Pink area of graph represents optimal ranges for all properties and molecule having all 6 axes lying within pink area may be considered to be orally bioavailable. Optimum ranges for each axes represented are as follows.

1. Lipophilicity (LIPO): XLOGP3 between − 0.7 and + 5.0
2. Size: molecular weight between 150-500 g/mol
3. Polarity (POLAR): Topological Polar Surface Area (TPSA) between 20-130 A°
4. Solubility (INSOLU): log S not higher than 6
5. Saturation (INSATU): fraction of carbons in the sp3 hybridization not less than 0.25
6. Flexibility (FLEX): No more than 9 rotatable bond


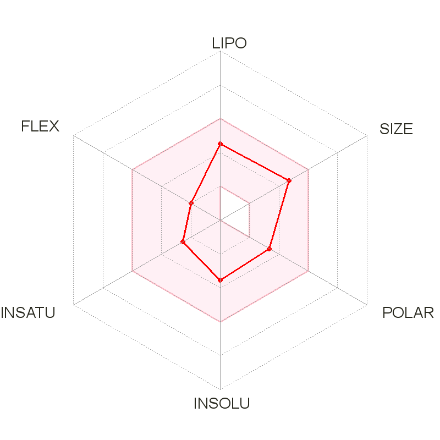

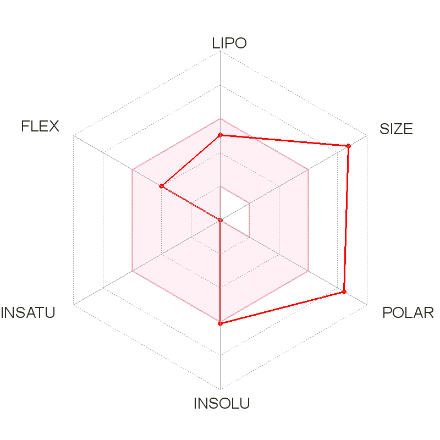

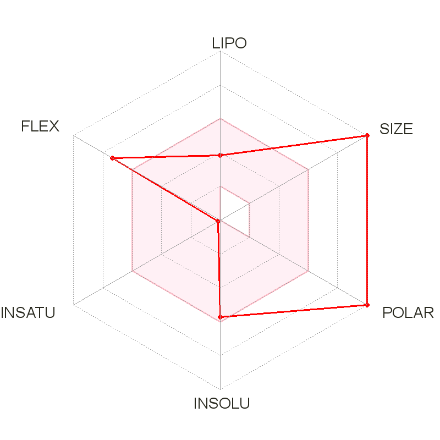

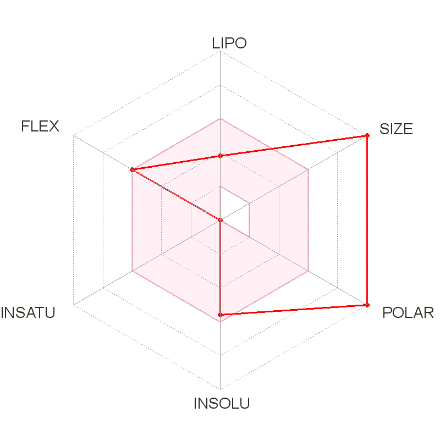

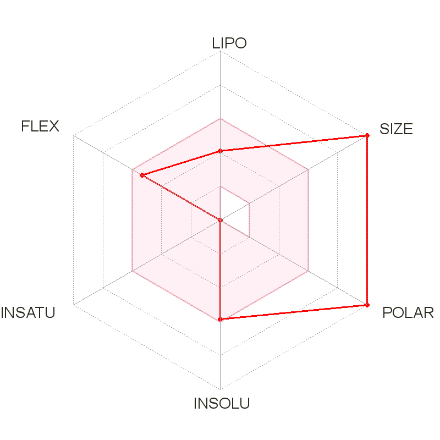

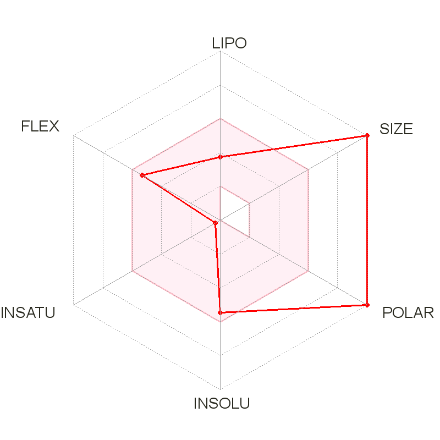

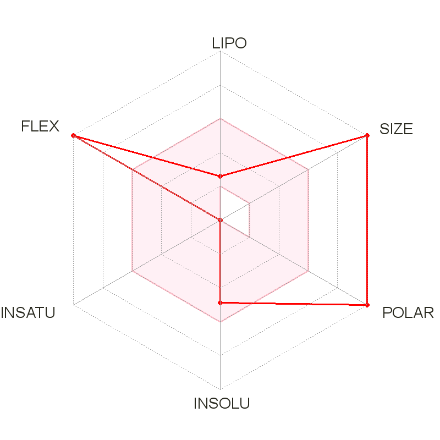

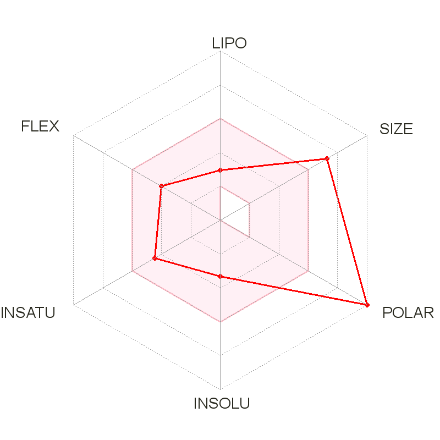

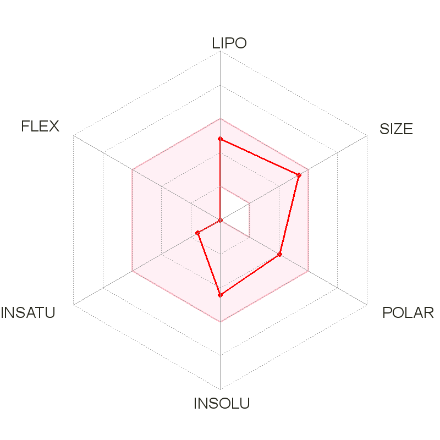


Asparagamin A

Asparanin A

Shatavarin X

Shatavarin IX

Shatavarin IV

Shatavarin VII

***Asparagus racemosus***

Shatavarin I

Rutin

Muzanzagenin

Shatavarin VI

Isoagatharesinol


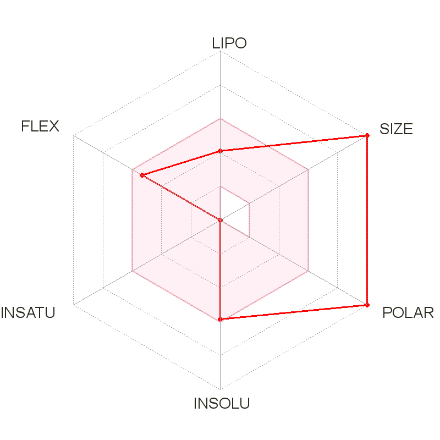

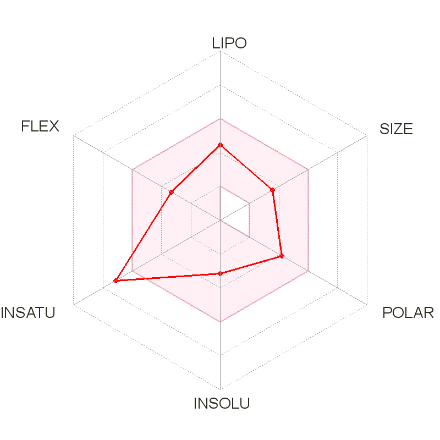


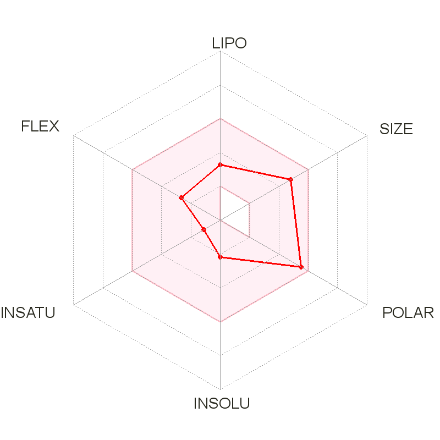

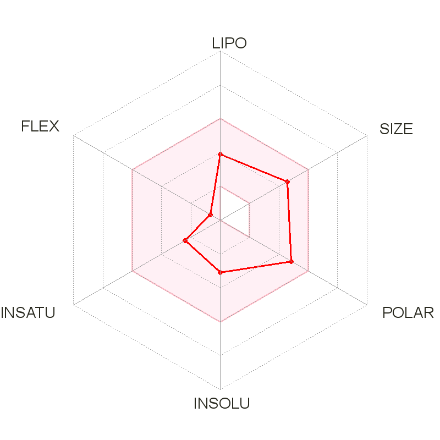

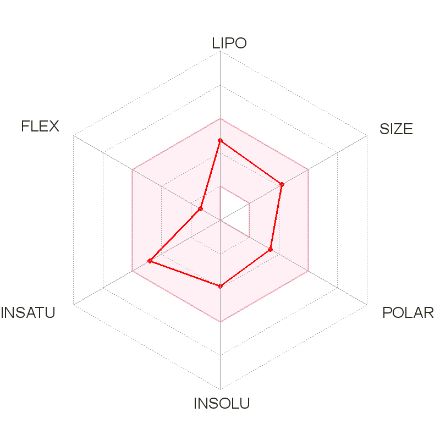

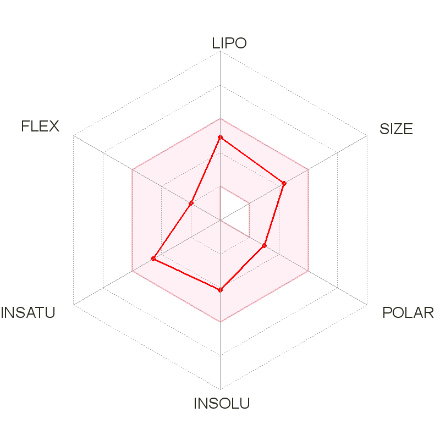

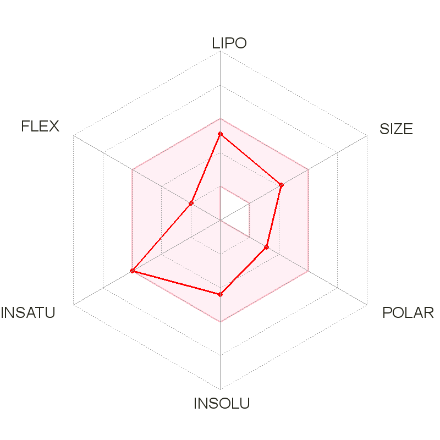

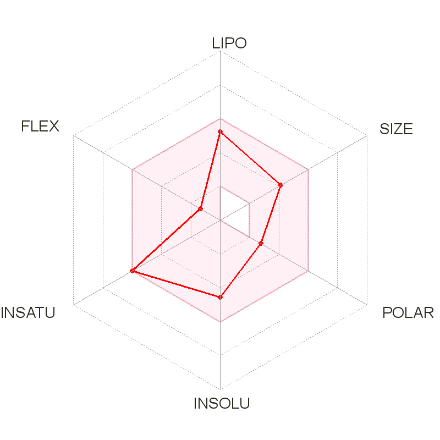


***Tinospora cordifolia***

Menisperine

Columbamine

Berberine

Tinocordiside

Tinosporide

Magnoflorine

20-β-Hydroxy Ecdysone

Columbin

Syringin


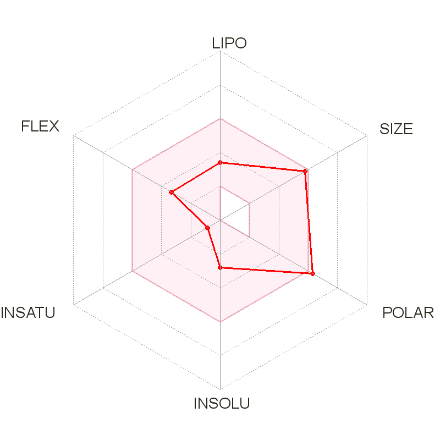

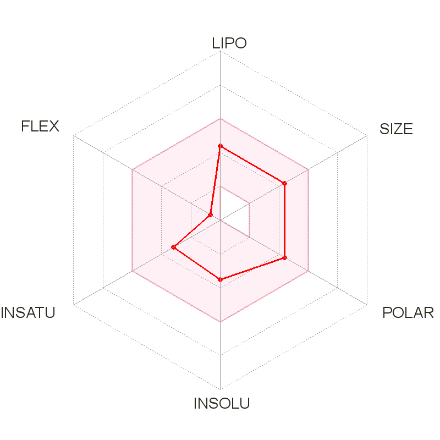

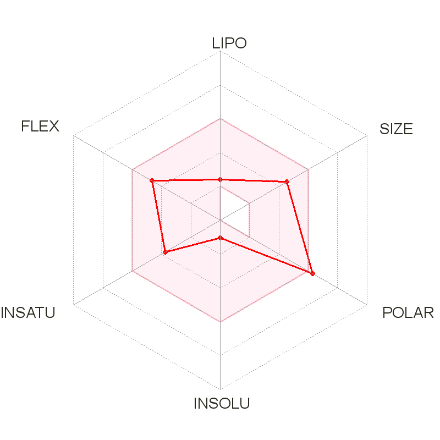


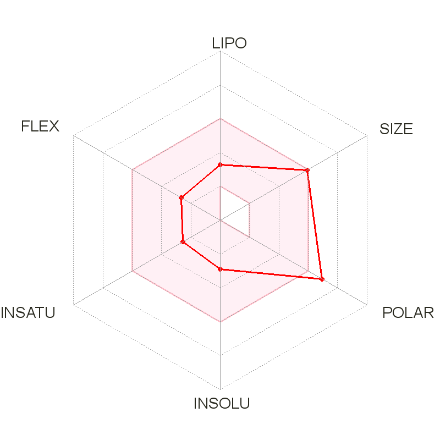


Tinosporaside

***Withania somnifera***

Withastramonolide-12-Deoxy

Withanolide B

Withacoagin


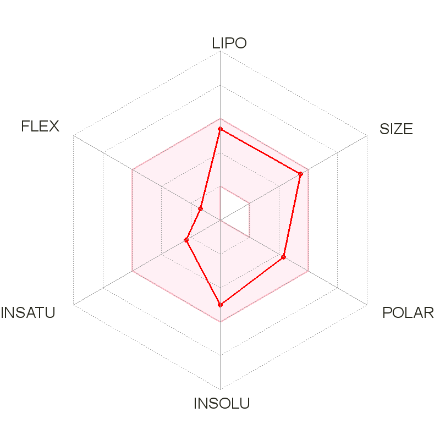


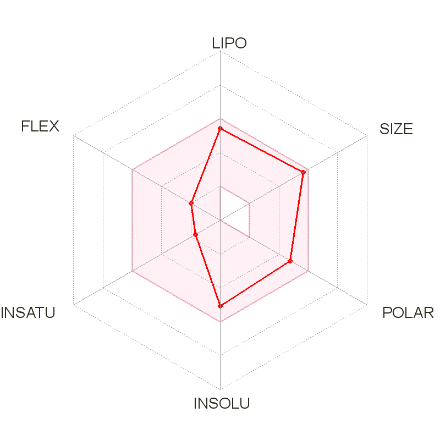

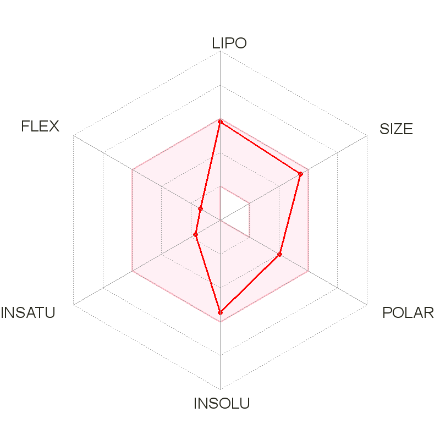


Withanone

Withanolide A

Withanoside IV


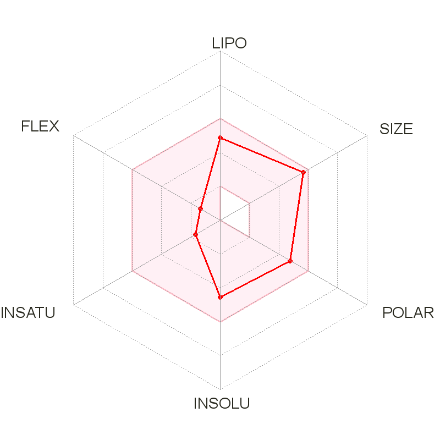

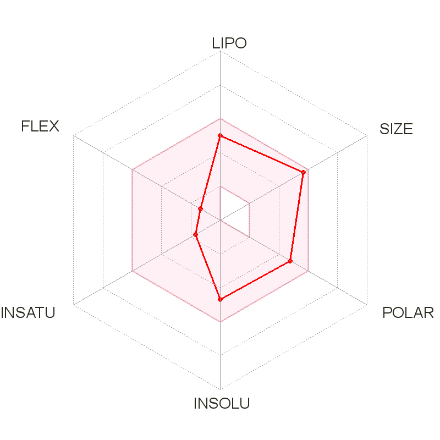

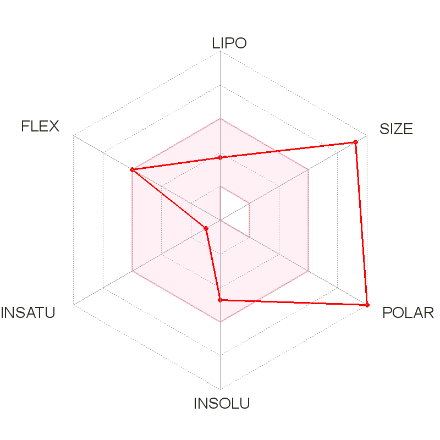


Withanoside V

27-hydroxy Withanone

Withaferin A


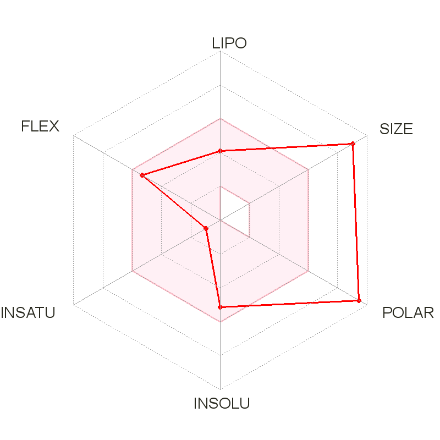

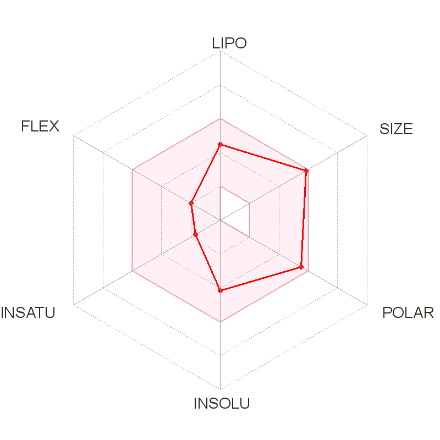

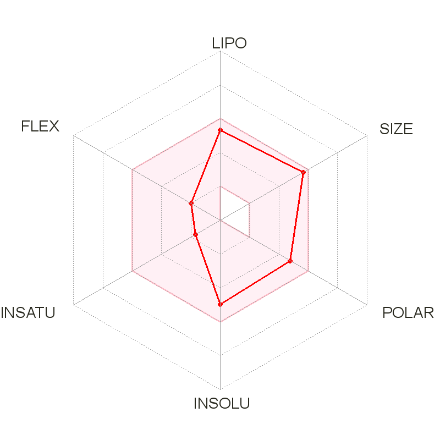


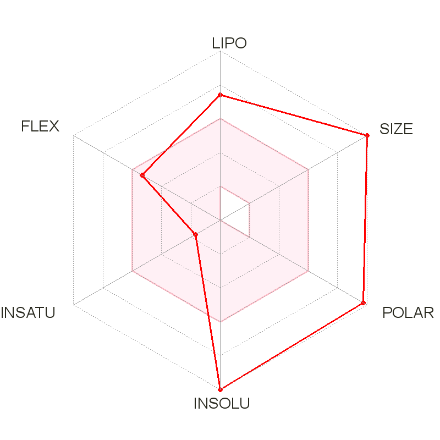


Ashwagandhanolide

**S4 Table. *In vitro* effect of AR, TC and WS extracts on major CYP isoforms.**

| **Herbs** | **Type of extract** | **Name of enzyme** | **IC_50_ value (µg/ml)** | **References** |
| --- | --- | --- | --- | --- |
| *Asparagus racemosus* | Aqueous | CYP3A4 | 656.391 | ^1^ |
| *Tinosporacordifolia* | Aqueous | CYP3A4 | 594.1 | ^2^ |
|  | Hydro alcoholic |  | 136.45 | ^3^ |
|  | Hydro alcoholic | CYP1A2 | 141.82 |  |
|  |  | CYP2C9 | 127.55 |  |
|  |  | CYP2D6 | 144.37 |  |
| *Withaniasomnifera* | Aqueous, Methanolic, Hydromethanolic and Ethanolic | CYP1A2 | >100 | ^2,4,5^ |
|  | Aqueous, Methanolic, Hydromethanolic and Ethanolic | CYP2C9 | >100 |  |
|  | Aqueous, Methanolic, Hydromethanolic and Ethanolic | CYP2D6 | 326.0, 184.0, >640 and 142.0 |  |
|  | Aqueous, Methanolic, Hydromethanolic and Ethanolic | CYP3A4 | >1000, >640, >640 and 170.6 |  |

Note: This table has been prepared from the published literature

1. Borse, S. P. & Kamble, B. B. Effects of Ayurvedic Rasayana botanicals on CYP3A4 isoenzyme system. *J. Integr. Med.***13**, 165–172 (2015).

2. Patil, D., Gautam, M., Gairola, S., Jadhav, S. & Patwardhan, B. Effect of botanical immunomodulators on human CYP3A4 inhibition: Implications for concurrent use as adjuvants in cancer therapy. *Integr. Cancer Ther.***13**, 167–175 (2014).

3. Bahadur, S. *et al.* Metabolism-mediated interaction potential of standardized extract of Tinospora cordifolia through rat and human liver microsomes. *Indian J. Pharmacol.***48**, 576–581 (2016).

4. Savai, J., Varghese, A., Pandita, N. & Chintamaneni, M. Investigation of CYP3A4 and CYP2D6 interactions of withania somnifera and centella asiatica in human liver microsomes. *Phyther. Res.***29**, 785–790 (2015).

5. Savai, J., Varghese, A., Pandita, N. & Chintamaneni, M. In vitro assessment of CYP1A2 and 2C9 inhibition potential of Withania somnifera and Centella asiatica in human liver microsomes. *Drug Metab. Pers. Ther.***30**, 137–141 (2015).

**S5 Table. Pharmacokinetic data of WHO solidarity trial drugs and commonly prescribed drugs for hypertension, asthma and T2DM.**

| Drug category | Name of drug | Bioavailability  (%) | PPB  (%) | Peak plasma concentration  (C_max_) | Mini. Effec.  Conc for SARS-nCOV2 | Clearance rate | t_1/2_ | Associated CYP enzyme | IC50 value for  CYP  enzyme |
| --- | --- | --- | --- | --- | --- | --- | --- | --- | --- |
| WHO solidarity trial drugs for COVID 19 | **Remdesivir**^1,2,3^**:**  (loading dose: 200 mg followed by 100 mg daily IV for 10 days) | 88 | Moderate | Loading dose: 5440 ±20.3ng/ml 2610±12.7 ng/mL (after 5-day dosing)  Active metabolite: 142 ±30.3 ng/mL | EC50 values below 1 uM (=approximately 0.6 ug/mL)  in vitro activity against SARS-CoV-2 with an EC50 at 48 hours of 0.77 µM in Vero E6 cells | NA | 1 hour  Metabolite: 24.5 hours | CYP2C8, CYP2D6 (S) and CYP3A4 (SINH) | NA |
|  | **Chloroquine**^1,4,5^**:** Adults with a bodyweight ≥ 50 kg: 500 mg twice daily for 7 days  (Adults with a bodyweight < 50 kg: 500 mg twice daily on days 1 and 2, followed by 500 mg once daily for days 3-7) | 80  *(S=66.6±3.3  R=42.7±2.1) | 50-60 | PO: 76± 14  IM: 57-480  IV:837-248  (ng/ml) | 15-30 ng/ml (for malaria)  EC50 value for chloroquine is 1.13 µM  against SARS-CoV-2 with an EC50 at 48 hours of 1.13 µM in Vero E6 cells | 0.35-1L/h/kg | 10-24 days | CYP3A4 and CYP2C8 (S)/ CYP2D6 (S INH) | CYP2D6 =  44 µmol/L |
|  | **Hydroxychloroquine**^145^**:**(800 mg x 2 loading dose followed by 400 mg x 2 every day for a total of 10 days) | 45±03 | 50 | 46 (34-79) | against SARS-CoV-2 with an EC50 of 0.72 µM in Vero E6 cells | 96 mL/min | 44  (26-63) days | CYP3A4 and CYP2C8 (S)/ CYP2D6 (INH) | NA |
|  | **Ritonavir/Lopinavir**^1,6,7,8,9,10,11^**:**(400/100 mg twice daily) for 14 days PO | 98±01 | 98-99 | 9.8 ± 3.7 μg/mL | 1000 ng/mL (for HIV)  (minimum intracellular and plasma concentrations at 8 and 4 μg/mL, respectively-KALEPHAR Study  2004)  Against SARS-CoV-1, EC50 17.1 ± 1 µM in Vero E6 cells  No in vitro data for LPV/r in SARS-CoV-2 exist | 5.98 ± 5.75 L/hr | Single dose: 2 to 3 hours Multiple-doses 4 to 6 hours | CYP3A4 (SINH) | Ritonavir-  0.14 µM  Lopinavir-  7.3 µM |
|  | **Ritonavir/lopinavir +**  **interferon-beta 1a**^12,13,14^**:**(0.25mg subcutaneous injection alternate day for 3 days) | 16 |  | 1.3 IU/ml | 1.51 ng/ml |  | 5-10 h | CYP3A4 (SINH) for Ritonavir/Lopinavir and CYP1A2 (INH) |  |
| Anti-hypertensive | **Propranolol**^15,16^**:** (10 mg, 20 mg, 40 mg, 60 mg, and 80 mg tablets for oral administration) | 50 | 90 | 26±1 ng/mL | NA | 810 mL/min (Hypertensive adults) | 3-6 hr | CYP2D6 (SINH) | Not found |
|  | **Metoprolol**^17,18^**:** (50 mg, 100mg tablets for oral administartion) | 50 | 11 | Not found | NA | 0.8 L/min | 3-7 hr | CYP2D6 (SINH) | Not found |
|  | **Telmisartan**^19,20^**:** (20mg,40mg and 80mg tablets for oral administration) | Dose dependent (e.g. for 40 mg dose bioavailability = 42%) | 99.5 | 17.9 ng/mL after single dose of 20mg | NA | >800 mL/min | 24 hr | CYP2C9 (INH) | 41.9 µM |
|  | **Losartan**^21^: (25mg,50mg and 100mg tablets for oral administration) | 33 | 98.6–98.8 for losartan  99.7 – for active metabolite E3174 | 0.29 µg/L for losartan  0.25 µg/L for E3174 | NA | 4.3 L/hr for losartan,  1.6 L/hr for E3174 | 2.1 hr for losartan,  6.4 hr for E3174 | CYP2C9 (SINH) | 39.5 µM |
| Anti-asthmatics | **Theophylline**^22,23,24^: (200mg,400mg and 800mg in 5% dextrose injection) | NA | 40 | NA | NA | 0.65 mL/kg/min [Adults (16-60 years), otherwise healthy non-smoking asthmatics] | 8 hr | CYP1A2 (SINH) | 120 µM |
| Anti-diabetic | **Glimepiride**^25,26^: (1mg,2mg and 4mg tablets for oral administration) | 107 | 99.5 | 102±48 ng/mL | NA | 47.8 mL/min. | 3.1±1.7 hours | CYP2C9 (S) | NA |
|  | **Pioglitazone**^27,28^**:** (15mg,30mg and 45mg tablets for oral administration) | 83 | 99 | 11.40±0.7 µg/ml | NA | 5-7 L/hr | 3-7 hr | CYP2C8 (SINH)/CYP3A4 (SINH) | 9.38 µM /12.3  µM |

Note: This is representative list for the drugs that are being used in associated comorbidities. (Here, S = Substrate, INH = Inhibitor, SINH = Substrate Inhibitor and NA = Not Applicable)

**References**:

1. McCreary, E. K. and J. M. P. Coronavirus Disease 2019 Treatment: A Review of Early and Emerging Options. *Open Forum Infect. Dis.* (2020).

2. Grein, J., et al. Compassionate Use of Remdesivir for Patients with Severe Covid-19. *N. Engl. J. Med.* (2020).

3. Summary on compassionate use Remdesivir Gilead International Nonproprietary Name : remdesivir. *Eur. Med. Agency***31**, 41 (2020).

4. Thummel, K.E., D.D. Shen, and N. I. *Design and optimization of dosage regimens: pharmacokinetic data, in Goodman and Gilman’s the pharmacological basis of therapeutics*. (McGraw Hil, 2018).

5. Projean, D. *et al.* In vitro metabolism of chloroquine: Identification of CYP2C8, CYP3A4, and CYP2D6 as the main isoforms catalyzing N-desethylchloroquine formation. *Drug Metab. Dispos.***31**, 748–754 (2003).

6. Breilh, D., et al. Virological, intracellular and plasma pharmacological parameters predicting response to lopinavir/ritonavir (KALEPHAR study). *Aids***18**, 1305–1310

7. Chandwani, A. and J. S. Lopinavir/ritonavir in the treatment of HIV-1 infection: a review. *Ther. Clin. Risk Manag.***4**, 1023 (2008).

8. Jackson, A., et al. Pharmacokinetics of plasma lopinavir/ritonavir following the administration of 400/100 mg, 200/150 mg and 200/50 mg twice daily in HIV-negative volunteers. *J. Antimicrob. Chemother.***66**, 635–640 (2011).

9. Lopez-Cortes, L.F., et al. Lopinavir plasma concentrations and virological outcome with lopinavir-ritonavir monotherapy in HIV-1-infected patients. *Antimicrob. Agents Chemother.***57**, 3746–3751 (2013).

10. August JT, Murad F, Anders MW, Coyle JT, L. A. *Drug-Drug interactions: scientific and regulatory perspectives*. (Academic Press, 1997).

11. Weemhoff, J. L. *et al.* Apparent mechanism-based inhibition of human CYP3A in-vitro by lopinavir. *J. Pharm. Pharmacol.***55**, 381–386 (2003).

12. Salmon, P., et al. Pharmacokinetics and pharmacodynamics of recombinant human interferon-β in healthy male volunteers. *J. Interf. cytokine Res.***16**, 759–764 (1996).

13. Hu, X., et al. COMPARE: Pharmacokinetic profiles of subcutaneous peginterferon beta‐1a and subcutaneous interferon beta‐1a over 2 weeks in healthy subjects. *Br. J. Clin. Pharmacol.***82**, 380–388 (2016).

14. Delaporte, E. and K. W. R. Cytochrome P4501A1 and cytochrome P4501A2 are downregulated at both transcriptional and post-transcriptional levels by conditions resulting in interferon-α/β induction. *Life Sci.***60**, 787–796 (1997).

15. Accessdata.fda. Inderal (Propranolol hydrochloride tablets). Available at: https://www.accessdata.fda.gov/drugsatfda_docs/label/2011/016418s080,016762s017,017683s008lbl.pdf.

16. Routledge, P. . & Shand, D. . Indwelling Cannulae 9.9 %. 74–90 (1977).

17. Morris J, D. A. Metoprolol. *InStatPearls [Internet]***StatPearls**, (2018).

18. Bahar, M. A., Kamp, J., Borgsteede, S. D., Hak, E. & Wilffert, B. The impact of CYP2D6 mediated drug–drug interaction: a systematic review on a combination of metoprolol and paroxetine/fluoxetine. *Br. J. Clin. Pharmacol.***84**, 2704–2715 (2018).

19. Accessdata.fda. MICARDIS® (telmisartan) Tablets. Available at: https://www.accessdata.fda.gov/drugsatfda_docs/label/2011/020850s032lbl.pdf.

20. Stangier, J., Su, C. A. P. F. & Roth, W. Pharmacokinetics of orally and intravenously administered telmisartan in healthy young and elderly volunteers and in hypertensive patients. *J. Int. Med. Res.***28**, 149–167 (2000).

21. Sica, D. A., Gehr, T. W. B. & Ghosh, S. Clinical pharmacokinetics of losartan. *Clin. Pharmacokinet.***44**, 797–814 (2005).

22. Haley, T. J. Metabolism and pharmacokinetics of theophylline in human neonates, children, and adults. *Drug Metab. Rev.***14**, 295–335 (1983).

23. Ha, H., Chen, J., Freiburghaus, A. & Follath, F. Metabolism of theophylline by cDNA‐expressed human cytochromes P‐450. *Br. J. Clin. Pharmacol.***39**, 321–326 (1995).

24. THEOPHYLLINE 200, 400 AND 800 mg in 5% Dextrose Injection, USP. Available at: https://www.accessdata.fda.gov/drugsatfda_docs/label/2008/019211s040lbl.pdf.

25. Accessdata.fda. AMARYL (glimepiride) tablets. (1995). Available at: https://www.accessdata.fda.gov/drugsatfda_docs/label/2013/020496s027lbl.pdf.

26. Badian, M., Lehr, K. H., Malerczyk, V., Korn, A. & Waldhäusl, W. Absolute Bioavailability of Glimepiride (AMARYL®) after Oral Administration. *Drug Metabol. Drug Interact.***11**, 331–340 (1994).

27. Accessdata.fda. ACTOS^TM^ (Pioglitazone Hydrochloride) Tablets. (1999). Available at: https://www.accessdata.fda.gov/drugsatfda_docs/label/1999/21073lbl.pdf.

28. Hanefeld M. Pharmacokinetics and clinical efficacy of pioglitazone. *Int. J. Clin. Pract.***121**, 19–25 (2001).
